# Impacts on Breastfeeding Practices of At-Scale Strategies That Combine Intensive Interpersonal Counseling, Mass Media, and Community Mobilization: Results of Cluster-Randomized Program Evaluations in Bangladesh and Viet Nam

**DOI:** 10.1371/journal.pmed.1002159

**Published:** 2016-10-25

**Authors:** Purnima Menon, Phuong Hong Nguyen, Kuntal Kumar Saha, Adiba Khaled, Andrew Kennedy, Lan Mai Tran, Tina Sanghvi, Nemat Hajeebhoy, Jean Baker, Silvia Alayon, Kaosar Afsana, Raisul Haque, Edward A. Frongillo, Marie T. Ruel, Rahul Rawat

**Affiliations:** 1 Poverty, Health and Nutrition Division, International Food Policy Research Institute, Washington, District of Columbia, United States of America; 2 Alive & Thrive, FHI360, Washington, District of Columbia, United States of America; 3 Save the Children, Washington, District of Columbia, United States of America; 4 BRAC, Dhaka, Bangladesh; 5 University of South Carolina, Columbia, South Carolina, United States of America; Institute for Global Health, UNITED KINGDOM

## Abstract

**Background:**

Despite recommendations supporting optimal breastfeeding, the number of women practicing exclusive breastfeeding (EBF) remains low, and few interventions have demonstrated implementation and impact at scale. Alive & Thrive was implemented over a period of 6 y (2009–2014) and aimed to improve breastfeeding practices through intensified interpersonal counseling (IPC), mass media (MM), and community mobilization (CM) intervention components delivered at scale in the context of policy advocacy (PA) in Bangladesh and Viet Nam. In Bangladesh, IPC was delivered through a large non-governmental health program; in Viet Nam, it was integrated into government health facilities. This study evaluated the population-level impact of intensified IPC, MM, CM, and PA (intensive) compared to standard nutrition counseling and less intensive MM, CM, and PA (non-intensive) on breastfeeding practices in these two countries.

**Methods and Findings:**

A cluster-randomized evaluation design was employed in each country. For the evaluation sample, 20 sub-districts in Bangladesh and 40 communes in Viet Nam were randomized to either the intensive or the non-intensive group. Cross-sectional surveys (*n* ~ 500 children 0–5.9 mo old per group per country) were implemented at baseline (June 7–August 29, 2010, in Viet Nam; April 28–June 26, 2010, in Bangladesh) and endline (June 16–August 30, 2014, in Viet Nam; April 20–June 23, 2014, in Bangladesh). Difference-in-differences estimates (DDEs) of impact were calculated, adjusting for clustering. In Bangladesh, improvements were significantly greater in the intensive compared to the non-intensive group for the proportion of women who reported practicing EBF in the previous 24 h (DDE 36.2 percentage points [pp], 95% CI 21.0–51.5, *p <* 0.001; prevalence in intensive group rose from 48.5% to 87.6%) and engaging in early initiation of breastfeeding (EIBF) (16.7 pp, 95% CI 2.8–30.6, *p =* 0.021; 63.7% to 94.2%). In Viet Nam, EBF increases were greater in the intensive group (27.9 pp, 95% CI 17.7–38.1, *p <* 0.001; 18.9% to 57.8%); EIBF declined (60.0% to 53.2%) in the intensive group, but less than in the non-intensive group (57.4% to 40.6%; DDE 10.0 pp, 95% CI −1.3 to 21.4, *p =* 0.072). Our impact estimates may underestimate the full potential of such a multipronged intervention because the evaluation lacked a “pure control” area with no MM or national/provincial PA.

**Conclusions:**

At-scale interventions combining intensive IPC with MM, CM, and PA had greater positive impacts on breastfeeding practices in Bangladesh and Viet Nam than standard counseling with less intensive MM, CM, and PA. To our knowledge, this study is the first to document implementation and impacts of breastfeeding promotion at scale using rigorous evaluation designs. Strategies to design and deliver similar programs could improve breastfeeding practices in other contexts.

**Trial registration:**

ClinicalTrials.gov NCT01678716 (Bangladesh) and NCT01676623 (Viet Nam)

## Abstract 2

Phuong Nguyen and colleagues present a cluster-randomized program evaluation of strategies combining intensive interpersonal counseling, mass media, and community mobilization to improve breastfeeding practices.

## Author Summary

**Why Was This Study Done?:**

- The benefits of exclusive breastfeeding are well documented, but only about a third of infants are exclusively breastfed during their first 6 months of life, well short of the 50% target endorsed by the World Health Assembly.
- Past efforts to promote and support breastfeeding were rarely implemented at scale.
- Previous studies on strategies to support breastfeeding have been limited to efficacy research; the few studies of programmatic approaches did not use a rigorous evaluation design.

**What Did the Researchers Do and Find?:**

- We conducted two cluster-randomized impact evaluations of at-scale programs, in Bangladesh and Viet Nam (the Alive & Thrive initiative), to compare the population-level impacts on breastfeeding practices of two intervention packages: (1) an intensive package consisting of intensified interpersonal communication on breastfeeding, a mass media campaign, community mobilization, and policy advocacy to create a supportive environment for optimal breastfeeding practices and (2) a non-intensive package consisting of standard nutrition counseling on breastfeeding and a less intensive mass media campaign, community mobilization, and policy advocacy.
- Surveys were conducted among households with children 0–5.9 mo of age at two points in time, before the interventions started (2010) and four years later (2014).
- In both countries, we found significantly positive population-level impacts on breastfeeding practices, including higher rates of early initiation of breastfeeding and exclusive breastfeeding, and lower use of prelacteal feeding and bottle feeding, in areas that received the intensive package compared to areas that received the non-intensive package.

**What Do These Findings Mean?:**

- These findings provide much-needed evidence on what works to improve breastfeeding at scale.
- Combining interpersonal counseling of mothers on optimal breastfeeding practices with a mass media campaign is more effective than a mass media campaign alone, suggesting that using multiple platforms and interventions to improve breastfeeding practices leads to greater improvements in practices than using one strategy alone.
- The well-documented approach used by Alive & Thrive offers several implementation tools that can be used and adapted to inform the design of contextually relevant interventions to improve breastfeeding, a critical human development intervention.

## Introduction

Globally, only 38% of infants are exclusively breastfed during their first 6 mo of life [1,2]. One of the six Global Nutrition Targets 2025 endorsed by the World Health Assembly is to “increase the rate of exclusive breastfeeding in the first 6 months up to at least 50%” [3]. Despite the well-documented benefits of exclusive breastfeeding (EBF) [4,5], and longstanding inclusion of interventions to improve EBF in child survival [6] and nutrition [4] strategies, tracking of global child survival goals shows slow progress in achieving adequate EBF levels in most developed and developing countries [2,7]. Past EBF promotion approaches, although often successful [8–10], have generally failed to achieve and maintain scale [11–13]. Achieving the ambitious World Health Assembly target for EBF will require a greater focus on effective delivery platforms [11] with multipronged approaches to implement successful programs at scale [10], including approaches that tightly link policy advocacy, investments in human resource development, data-driven implementation, research, and evaluation [12].

Suboptimal breastfeeding practices, including non-exclusive breastfeeding, contribute to 11.6% of mortality in children under 5 y of age [1]. Breastfeeding also prevents illnesses and provides essential nutrients for optimal child growth and development during the first 2 y [1,14,15], conveys several benefits for the mother [16], and may even have long-term effects on IQ and income [17]. Currently, the World Health Organization recommends initiation of breastfeeding within 1 h of birth, EBF until 6 mo of age, and continued breastfeeding until 2 y of age or beyond.

Systematic reviews have documented the impact on breastfeeding practices of various approaches, including those based on the use of individual- and group-based peer counseling and contact with lay counselors or trained personnel [8,9]. The studies reviewed, however, are generally implemented at small scale and under relatively controlled conditions. The reviews highlight the need to rigorously test the effectiveness of these and similar strategies when implemented at large scale. Although one study reports on the results of at-scale programs [18], the adequacy design used [19] does not provide irrevocable evidence that shifts in practices were attributable to the intervention strategies promoted by the program. A new review [10] provides evidence that combinations of interventions delivered through different platforms are more effective than single interventions, and recommends incorporation of combined strategies using complex adaptive systems for scaling up. However, to our knowledge, there are no studies, that combine at-scale programming with a rigorous evaluation using a randomized study design. Therefore, public health evidence on whether the magnitude of impacts on breastfeeding practices seen in efficacy studies and synthesized in systematic reviews can be achieved when programs operate at scale under real-life conditions is lacking [11,12].

This paper reports on findings from cluster-randomized impact evaluations of two at-scale programs, in Bangladesh and Viet Nam, that provided intensified interpersonal counseling (IPC) to pregnant women and mothers of children up to 2 y of age, combined with mass media (MM), community mobilization (CM), and policy advocacy (PA) intervention components, to improve breastfeeding practices [20]. The two program models that were evaluated reached large scale through distinct delivery platforms. In Bangladesh, IPC and CM were delivered by the community-based health platform of a large non-governmental organization (BRAC) through its network of field officers, community-based frontline workers (FLWs), and volunteers. In Viet Nam, IPC and CM were delivered through a social franchising approach integrated within the facility-based government health system [21]. In both countries, a nationwide MM campaign was implemented, as well as PA, to create a supportive environment for optimal infant feeding practices. Both implementation models used data-driven approaches that were focused on impact and delivery at scale and adapted to implementation conditions and context, thus fulfilling several elements recently identified as critical to scaling up impact on nutrition [22], and specific elements central to scaling up breastfeeding in particular [12].

## Methods

### Ethical Approval

Approval for the study was obtained from the institutional review board at the International Food Policy Research Institute (IRB #00007490), the Bangladesh Medical Research Council (IRB #BMRC/NREC/2007-2010/99), and the Vietnam Union of Science and Technology Associations (IRB #0904). All mothers of study children were provided with detailed information about the study verbally and in writing at recruitment. Verbal informed consent was obtained from mothers in Bangladesh; written informed consent was obtained in Viet Nam.

### Evaluation Design

A cluster-randomized, non-blinded impact evaluation design was employed in each country to compare the impact of two Alive & Thrive (A&T) intervention packages, i.e., an intensive package consisting of intensified IPC, MM, CM, and PA compared to a non-intensive package consisting of standard nutrition counseling on breastfeeding along with less-intensive MM, CM, and PA on the primary and secondary outcomes of interest. Table 1 describes the interventions and the differences between the intensive and non-intensive packages. Cross-sectional household surveys were conducted at baseline (2010) and 4 y later (2014) in the same communities, among households with children under 6 mo of age. The questionnaires administered at each survey round were the same, with the exception of a detailed set of questions to measure program exposure included at endline. Within a large rollout of the intensive intervention package in both countries, 20 sub-districts (upazilas) in Bangladesh and 40 communes in Viet Nam were randomized to either the intensive or non-intensive intervention. The technical rationale for the impact and process evaluation approach used for A&T is available elsewhere [23,24]. The process evaluation used a variety of methods, tailored to implementation timelines, and data were collected in 2011, 2012, and 2013 in both countries. In both countries, the evaluation was restricted to a smaller geographic area than the full coverage area of the program.

**Table 1:** Description of differences in interventions between intensive and non-intensive intervention groups in Bangladesh and Viet Nam.

Intervention Component	Bangladesh		Viet Nam	
	Non-intensive	Intensive	Non-intensive	Intensive
Interpersonal counseling/standard nutrition education	Standard care and nutrition counseling by BRAC health volunteers, called Shasthya Sebika (SS), who are assigned 250–300 households in their community; they are expected to visit households monthly, provide health products mainly related to maternal and child health, give messages for ten key health topics including BF and immunization, refer for child illnesses, help identify pregnancies and link women to ANC, and be present at or soon after a delivery. BRAC health workers, called Shasthya Kormi (SK) (one per 2,500–3,000 households), provide ANC and PNC to mothers who choose to use BRAC’s services and motivate mothers to deliver at a health facility.	Standard care (as described in non-intensive), plus added intensified counseling: additional emphasis on early initiation of BF, no prelacteals, and EBF during ANC and PNC services provided by the SK; facilitation of initiation of BF within 1 h of birth, incentivized by SS; incentivized counseling on IYCF by SS during monthly door-to-door visits to all households with children under 2 y of age; counseling by full-time dedicated IYCF worker, called Pushti Kormi (one per 2,000–2,500 households), to address difficulties, complete volunteer records of home visits, train mothers at home on complementary feeding at 6 mo, and ensure the following schedule of contacts with mothers during the first 2 y: monthly from 0 to 8 mo, every other month to 12 mo, and one more visit each between 15–18 and 23–24 mo; additional emphasis given to IYCF at community meetings facilitated by BRAC’s ANC/PNC provider in the community with pregnant and recently delivered women. More information: http://aliveandthrive.org/resources/manual-implementation-of-community-based-interventions-for-infant-and-young-child-feeding-program-in-bangladesh/; http://aliveandthrive.org/wp-content/uploads/2014/11/BRAC-Final-report-8.28.2014.pdf.	Standard government health services provided at health centers including: provision of at least three antenatal visits; weighing children under 5 y twice a year; food demonstrations; basic nutrition counseling on child feeding; routine immunization; national vitamin A supplementation and micronutrient days to raise awareness of micronutrients and encourage consumption of micronutrient-rich foods/supplements.	Standard government health services (same as non-intensive)., plus added intensive structured package of eight BF counseling sessions (either group or individual) at social franchise set up at government health facilities addressing the following: preparation for EBF through three counseling sessions during the third trimester of pregnancy; personalized support for initiation of BF at the time of delivery (one contact); support and management of EBF through four counseling sessions during first 4 mo of infancy. Counseling sessions included leaflets, mother-and-child booklets, and distribution of promotional items to mothers who attended counseling sessions (face cloths, raincoats). More information: http://aliveandthrive.org/wp-content/uploads/2014/11/Overview-of-the-Social-Franchise-Model.pdf.
Mass media	**TV and radio spots:** Two BF-focused TV commercials aired via the four most-watched (by rural mothers) national channels and programs 6–12 times a day on 3 d of the week: (1) “Early Initiation” (http://aliveandthrive.org/resources/tv-spot-early-initiation-of-breastfeeding-bangladesh/); (2) “Insufficient Milk” (http://aliveandthrive.org/resources/tv-spot-perception-of-insufficient-milk-bangladesh/). Radio stories based on the TV commercials aired on four radio stations and news/sports programs targeted to male audiences. . . .	**TV spots:** Same as non-intensive. Added screening of same TV spots plus a cartoon film on BF (Meena) via community-based video shows in areas where TV reach was lower (“media dark” strategies), accompanied by community dialogue and quiz shows.	**TV spots:** Three BF-focused TV commercials aired via 18 national and provincial channels: (1) “No Water” (http://aliveandthrive.org/resources/tv-spot-no-water-viet-nam/); (2) “Breastmilk Only” (http://aliveandthrive.org/resources/tv-spot-breastmilk-only-viet-nam/); (3) “Promotion of Mat Troi Be Tho Franchises” (http://aliveandthrive.org/resources/tv-spot-promotion-of-mat-troi-be-tho-franchises-viet-nam/). **Out of home:** Sign boards promoting EBF and/or use of the counseling facility in 15 provinces. LCD TV screens showing the A&T TV spots in supermarkets and hospitals. **Digital:** Website and fan page for the social franchise model (Mat Troi Be Tho).Online digital posters on other popular websites. Mobile phone application.	**TV spots:** Same as non-intensive. **Out of home:** Same as non-intensive, with added loudspeaker announcements (five loudspeaker scripts). **Digital:** Same as non-intensive. **Other:** Posters on BF at health centers.
Community mobilization	Basic community mobilization through local meetings on topics such as family planning, pregnancy registration, and ANC.	Local meetings organized by BRAC managers to introduce the A&T program and activities of FLWs—targeted to school teachers, adolescents, religious leaders, village doctors, district hospital/clinic doctors, community elites—on the topic of nutrition and IYCF; fathers meetings added, with emphasis on handwashing linked to complementary feeding. Village theater shows for the general public to generate discussion about the importance of nutrition and IYCF and the role of the FLWs.	Village health workers mobilize communities to attend routine health services.	Village health workers visit homes to deliver invitation cards to the counseling sessions.
Policy advocacy	**National:** Technical support to a national IYCF alliance. Engagement with technical stakeholders via one-to-one and group workshops to share data and results on IYCF research. Journalist outreach and engagement to increase coverage in numbers of articles/TV talk shows and the depth of treatment of nutrition and IYCF.	**National:** Same as non-intensive.	**National:** Technical and financial support to National Nutrition Strategy and IYCF Action Plan; integration of IYCF into provincial nutrition plans; strengthening of the national Code on Marketing of Breast Milk Substitutes (Decree 21); strengthening of the national maternity leave policy, health insurance, insurance law, and workplace support programs. Media outreach.	**National:** Same as non-intensive. **Provincial:** Technical and financial support for developing annual provincial plans for nutrition.

ANC, antenatal care; BF, breastfeeding; EBF, exclusive breastfeeding; FLW, frontline worker; IYCF, infant and young child feeding; PNC, postnatal care; SK, Shasthya Kormi; SS, Shasthya Sebika.

In both countries, the evaluation was conducted in a subset of the total number of geographic areas chosen for programming. In Bangladesh, the program areas and the subset of evaluation areas were both rural. In Viet Nam, the program areas included both urban and rural areas, but the subset of evaluation areas were rural. At the outset of the evaluation, given the challenges of generalizability of a combined urban-rural evaluation model in Viet Nam, a decision was made to focus on rural areas only for the evaluation. By selecting rural areas only, we tried to ensure that the pool of areas included in the randomized allocation were somewhat homogenous.

The implementation teams were distinct from the evaluation teams. In Bangladesh, the implementation team was BRAC and FHI360, and the data collection team came from DATA, a survey research firm that had limited interactions with the implementation team. In Viet Nam, the implementation team was Save the Children and the government health system, and the data collection team came from the Institute of Social and Medical Studies. International Food Policy Research Institute researchers in charge of the evaluation connected and shared the information between the implementation and evaluation teams.

The study was registered at ClinicalTrials.gov in August 2012, about 2 y after the start of the program evaluation (April 2010). The sole reason for the delay in registering the trial was our understanding, at the time the study was initiated, that a program evaluation did not warrant registration, given the distinct differences between program evaluations and standard clinical trials. However, as discussions grew in the program evaluation community for preregistration of impact evaluations [25], a decision was made to register this program evaluation in a trial registry to ensure transparency and public availability of this information. No changes were made to the planned evaluation design or the planned analyses in the period between the design of the evaluation in 2010 and the registration of the evaluation in at ClinicalTrials.gov in 2012.

### Interventions

In Bangladesh, BRAC delivered intensified IPC and CM in 50 rural sub-districts in five of six divisions (later subdivided into seven) through its existing countrywide Essential Health Care program that already included standard counseling (S1 Fig) [26]. For standard nutrition counseling (available in both non-intensive and intensive areas), BRAC FLWs, called Shasthya Kormi, and health volunteers, called Shasthya Sebika, conducted routine home visits and provided standard information on infant and young child feeding (IYCF) practices, including breastfeeding. In intensive areas, IPC was based on multiple age-targeted IYCF-focused visits to households with pregnant women and mothers of children up to 2 y of age by the FLW and volunteer, as well as home visits by a nutrition-focused FLW, called Pushti Kormi, who was an additional human resource to provide more skilled support for breastfeeding and complementary feeding. In these areas, Shasthya Kormi and Pushti Kormi conducted monthly home visits and introduced age-appropriate IYCF practices, coached mothers as they tried out the practices, and engaged other family members to support the behaviors (see Table 1 for more details). These workers were supervised using observation checklists, and their workload was monitored. Cash incentives (US$6–US$8/mo) were given to the volunteers in the intensive areas for intervention delivery performance, which included ensuring high coverage, carrying out age-appropriate counseling at home visits, and collecting maternal reports of practicing the recommended behaviors. Households were unaware of this performance incentive. In intensive areas, CM included sensitization of community leaders to IYCF, and community theater shows focused on IYCF. The MM component consisted of the national broadcast of seven TV spots that targeted mothers, family members, health workers, and local doctors with messages on various aspects of IYCF; two of the spots focused on breastfeeding. Buys of media airtime were designed for multiple airings during the country’s most watched programs. In intensive areas that had limited electricity and TV access, supplemental activities were conducted to air the TV spots, and other IYCF films produced by the project, through local video screenings. PA included workshops to share data, engagement of journalists to broaden reporting on IYCF in the media, creation of an IYCF alliance and other such activities, which aimed at creating additional countrywide awareness of policies and programs to support breastfeeding.

In Viet Nam, standard nutrition counseling (available in both non-intensive and intensive areas) consisted of messages and information on IYCF delivered as part of routine child health care contacts at the government health facilities. Intensive areas received A&T program activities, which were implemented in 15 of 63 provinces (S2 Fig). Save the Children worked with the government of Viet Nam to establish a total of 781 government health facilities at the province, district, and commune levels that used a social franchising model, called Mat Troi Be Tho (MTBT), to deliver facility-based individual and group counseling. All facilities were required to meet minimum criteria including a standardized counseling room, trained staff, and availability of job aids and client materials. The program aimed to deliver 9 to 15 counseling contacts to each mother-child pair from the last trimester of pregnancy through the child’s first 2 y of life, including eight breastfeeding-focused contacts in the first 6 mo of life. Referrals, CM, promotional print materials, and TV advertising were used to generate demand for preventive IYCF counseling services, a concept new to most families. Training and supervision, incentives to the health facilities, and tools to collect data were applied to improve the quality of services. The MM component consisted of a nationally broadcast campaign using TV and the digital space (internet and mobile phone applications); three of four TV spots focused on breastfeeding. In intensive areas, the MM campaign also included additional out-of-home advertising through billboards and LCD screens. PA at the national and provincial levels targeted the extension of paid maternity leave to 6 mo, strengthening of the code of marketing of breast milk substitutes, and improving provincial planning for IYCF and nutrition actions.

**Fig 1:**
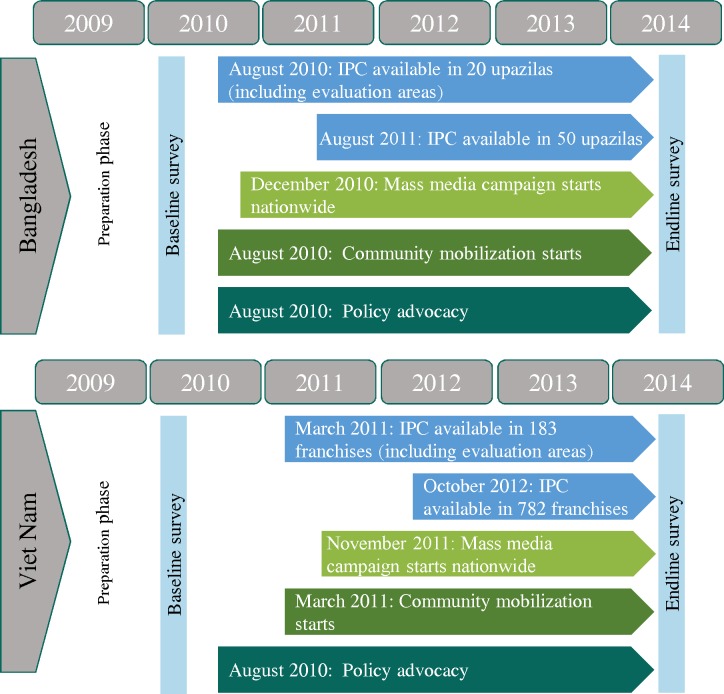
Rollout of interventions. IPC, interpersonal counseling.

The major components of the interventions started at the same time in all intervention areas in both Bangladesh and Viet Nam (Fig 1). In Bangladesh, intensified IPC began in 22 sub-districts (upazilas) by August 2010 and in another 28 sub-districts by August 2011. MM was launched in December 2010 and intensified to reach national coverage by February 2011. CM was operational in August 2010. In Viet Nam, IPC was initiated in March 2011, MM in November 2011, and CM in March 2011. PA activities commenced in August 2010 in both Bangladesh and Viet Nam. With the endline survey in 2014, the total duration of implementation of the full intensive package of interventions was about 3 y in both countries. All components continued uninterrupted throughout the implementation period once they had started. See Box 1 for information on A&T’s approach to operating at scale and Box 2 on the development of the MM campaign.

**Box 1. Delivering Infant and Young Child Feeding Interventions at Scale: The Alive & Thrive Approach**

The A&T programs in Bangladesh and Viet Nam operated from the outset with an “at scale mindset,” requiring the building of partnerships across diverse stakeholders. To reach large numbers of mothers with sufficient support for improving IYCF practices required the utilization of program platforms with existing high coverage such as the national primary health care system in Viet Nam and BRAC’s nationwide community-based networks of FLWs and volunteers in Bangladesh. Behavior change on a large scale required the strategic use of multiple harmonized communication channels with proven high reach and working with various types of opinion leaders. Mothers and family members needed to buy into benefits of the recommended practices that were of salience to themselves and their families. Mothers needed to feel confident about the feasibility of making changes, and families and mothers needed to perceive that social norms were shifting. The achievement of scale itself was intended to help shift the perception of social norms related to recommended feeding practices.

The A&T initiative was expected to demonstrate that improvement in IYCF could be achieved on a large scale in diverse contexts. Each country program was guided by formative research that narrowed the program focus to key leverage points for changing behaviors among mothers in each context, from among the many potential factors that influence IYCF practices. Streamlining was key for both countries and allowed us to set ambitious targets for the millions of pregnant women and mothers of children under 2 y to be reached with intensified IPC and for influential family/community members to be reached through multiple MM channels, advocacy, and CM. In 2013, at the height of implementation, approximately 17 million children under 2 y of age lived in the 50 of 493 sub-districts in Bangladesh where BRAC provided intensified IPC. Between December 2011 and February 2014, BRAC conducted almost 2.2 million home visits to mothers of children 0–5.9 mo. This required training more than 11,000 FLWs and supervisors in BRAC alone. Training was also provided to government staff and other stakeholders as part of support given to a national IYCF alliance formed for this purpose. Evidence of improved practices from early monitoring results, and an increasingly streamlined operational model with ready-made tools, facilitated other stakeholders’ joining the national IYCF initiative. The Viet Nam program worked with the government health system to establish an innovative social franchise model, MTBT, to deliver IYCF counseling at health facilities. Approximately 800,000 children under 2 y of age lived in 15 of the country’s 63 provinces where franchises were first established; between January 2012 and December 2014 there were 1.1 million counseling contacts with mothers of infants 0–5.9 mo of age at these franchises. Reaching this number of contacts required overcoming caregivers’ lack of experience in seeking preventive counseling and required community outreach and TV advertising to generate demand.

These large numbers of contacts were further enhanced with the addition of national MM campaigns to reach an estimated 8.5 million mothers of children below 2 y during more than 3 y in Bangladesh, and over 2 million mothers in Viet Nam. In addition, the media campaigns reached fathers, grandmothers, community leaders, physicians, village doctors, non-governmental organization workers, government FLWs, and policy makers. In each country, national and provincial or regional advocacy led to greater awareness of IYCF practices in communities and an improved enabling environment that supported investments in scaled-up programs and helped push the boundaries of scale even further.

For further reading, see the report “Scaling up in Agriculture, Rural Development, and Nutrition” [27] and the *Food and Nutrition Bulletin* 2013 supplement “Designing Large-Scale Programs to Improve Infant and Young Child Feeding in Asia and Africa” [28].

**Box 2. Development of the Alive & Thrive National Mass Media Campaigns**

A&T used proven processes to design, produce, and conduct its MM campaigns, following eight steps: (1) situation analysis, (2) formative research and a media audit, (3) media habit studies, (4) development of a communication strategy, (5) concept testing, leading to creative development of prototype materials, (6) material pretests followed by revisions, (7) material production, media planning, and media buys, and (8) campaign implementation and ongoing monitoring, leading to mid-course adjustments. The process ensured that A&T identified feasible behaviors for mothers to adopt, narrowing the focus to critical leverage points for changing behaviors, emotionally compelling storylines and audiovisuals, and strategic placement in the media to reach each audience segment repeatedly over a sustained period of time. In both Bangladesh and Viet Nam, media airs were timed to reach multiple audiences with frequent, repeated exposure with effective materials.

A&T’s MM campaigns were designed to influence the adoption of optimal breastfeeding behaviors by

1. greatly increasing the number of mothers repeatedly exposed to the program’s messages;
2. reaching additional audiences nationwide—fathers, grandmothers, physicians, village doctors and pharmacists, government and non-governmental organization FLWs—who influence and support mothers’ behaviors;
3. presenting compelling and positive stories that operate at an emotional level to help increase mothers’ self-efficacy and to shift the perception of social norms;
4. reinforcing FLWs’ and volunteers’ commitment to IYCF and reminding them of key messages;
5. lending credibility to community volunteers’ and health workers’ messages about breastfeeding by delivering them through “doctors” in TV stories with endorsements by the Ministry of Health and international agencies (in Viet Nam);
6. consistently delivering the program’s tested messages in a standardized way across thousands of communities and diverse audience segments;
7. reaching scale rapidly and preparing the ground for better uptake of messages delivered through direct interpersonal contacts that took several months to scale up;
8. provoking conversations about IYCF in communities, doctor offices, and families, sometimes for the first time, through exposure to deeply meaningful stories such as babies asking to be breastfed, a child saving his father’s life, and a new mother asserting her right to breastfeed her newborn immediately after delivery;
9. preventing a decline in motivation and knowledge/skills of FLWs following their training by acting as reminders over the years;
10. building community demand for the counseling services provided by health workers and community volunteers.

For further reading, see a description of A&T’s MM campaigns online [29].

### Primary and Secondary Outcomes

The primary outcome of the analysis presented in this paper is EBF in the previous 24 h, defined as the proportion of mothers of infants 0–5.9 mo of age who fed only breast milk (based on a previous-day recall of all foods and liquids) [30]. A key secondary outcome is early initiation of breastfeeding (EIBF), defined as the proportion of infants who were reported by mothers to have been put to the breast within 1 h of birth. Other related breastfeeding behaviors were also measured. Women with obstetric complications or cesarean delivery were included in the counts in determining EIBF. We categorized breastfeeding as exclusive, predominant (infant given water, water-based drinks, fruit juice, or ritual fluids in addition to breast milk), partial (infant given liquids and non-liquids such as milk, non-milk-based products, and other foods in addition to breast milk), and no breastfeeding [31], and also report EBF for the age bands 0–0.9 and 1–5.9 mo. We assessed prelacteal feeding based on maternal recall of foods consumed by the infant immediately after birth and during the first 3 d of life. Intervention exposure was measured by maternal recall of FLW home visits in the last 6 mo, recall of attendance at a CM event in the last 1 y, recall of ever having seen an A&T-promoted TV spot, and recall of specific breastfeeding messages contained within the TV spot. To help address the challenge of recall-based self-reported measures of breastfeeding, we collected information on infant diarrhea (defined as three or more loose stools in a 24-h period [32]).

### Sample Size Estimations

Sample size calculations were carried out to determine the sample size needed to detect differences in the primary outcome, i.e., EBF, between the two intervention groups at endline, considering an alpha of 0.05, power of 0.80, intra-class correlation (ICC) of 0.01 (estimated from previous national or sub-national surveys), and estimated baseline prevalence of EBF of 43% in Bangladesh and 30% in Viet Nam. We hypothesized that the intensive intervention would increase the primary outcome indicator, and therefore a one-sided test was used. For cost and logistical reasons, 20 clusters (sub-districts) were selected to be randomized in Bangladesh, and 40 clusters (communes) in Viet Nam. In Bangladesh we estimated that a sample size of 49 infants aged 0–5.9 mo per cluster—for a total sample of 980 (490 per group)—was sufficient to detect a difference at endline in the practice of EBF of 10 percentage points (pp) or larger. In Viet Nam, we estimated that a sample size of 23 infants per cluster—for a total sample of 920 (460 per group)—was sufficient to detect a minimum difference of 9 pp. Prior to conducting the endline survey, we re-verified sample size estimates based on the original sample size, the observed baseline EBF prevalence in our sample, and the ICC for EBF from the baseline survey (ICC of 0.04 for Viet Nam, and 0.1 for Bangladesh). No changes in sample sizes were made at the endline survey, based on these parameters and anticipated effect sizes based on the baseline survey.

### Randomization and Blinding

In Bangladesh, 100 sub-districts, across five divisions, were selected by BRAC as possible A&T intensive areas based on high poverty, stunting levels in excess of 30% among children < 5 y of age, and non-inclusion of the sub-district in the government National Nutrition Program. This list was narrowed to 78 based on geographic proximity (i.e., within the same agro-ecological and/or administrative zone, also called a division in Bangladesh), size, and other operational aspects to ensure homogeneity across the sample. The first phase of implementation took place in 50 of these sub-districts. Within each of five divisions, four sub-districts were randomly selected for inclusion in the evaluation sample using a computer program, for a total of 20 sub-districts. Sub-districts within each division were then randomly assigned, using a computer program, to either the intensive (10 sub-districts) or non-intensive (10 sub-districts) intervention. The randomization process was carried out in the presence of BRAC and A&T staff and the program evaluators in BRAC’s headquarters in Dhaka.

In Viet Nam, 15 provinces were selected for program implementation based on stunting levels, absence of other large organizations working in nutrition, population density, and representation of the different ecological regions covered by the initiative. Four rural provinces, representing four distinct ecological zones, were then selected for inclusion in the evaluation sample, and within these provinces, ten rural districts; two to six communes per district were selected for the evaluation sample based on the presence of a health center that met the eligibility criteria for the A&T franchise model; this ensured homogeneity across the sample. Communes were randomly assigned to either the intensive (20 communes) or non-intensive (20 communes) intervention. The randomization process was carried out using a simple public lottery system in the presence of local, district, and provincial health authorities as well as the program evaluators.

In both countries, households within the intensive and non-intensive areas were not explicitly made aware of the results of the randomization. Additionally, there was no blinding of the intervention at the level of service delivery.

### Sampling and Data Collection

In Bangladesh, within each sub-district, five unions and two villages within each union were randomly selected, to yield a total of 200 villages. Each village had an average size of 250 households. Within each village, a household census was conducted at baseline and endline to list mothers and infants and infants’ date of birth. A list of all households with infants less 6 mo of age was then drawn up. In Viet Nam, communes were sampled, and household listings were obtained from the health authorities, which maintain all birth records. In both countries, from these census lists that identified all households with children < 6 mo of age, households were selected for the survey using systematic sampling beginning with a random seed start point, to yield the desired sample size per cluster. Household visits for the survey were conducted only after sampling the desired number of households per village/commune. Desired sample sizes were 50 households per village in Bangladesh and 25 households per commune in Viet Nam. In Viet Nam, when there were insufficient infants within a commune due to small population sizes, we oversampled infants from another commune within the same district and intervention group.

A household questionnaire was administered to mothers of children under 6 mo of age to collect data on primary and secondary outcomes, along with data on several other household, maternal, and child characteristics (S1 Table). The interview was conducted in the local language, and almost all interviewees agreed to participate in the surveys. The refusal rate among mothers selected for inclusion through the random selection process and contacted was 0.9% for Bangladesh and 0.6% for Viet Nam. We excluded only mothers who had a clear mental disability, defined as inability to answer basic questions about their name or willingness to be interviewed that would prevent them from understanding and answering the questions.

### Statistical Analysis

Baseline differences between the two interventions were tested using ordinary least squares regression models (continuous variables) or logit regression models (categorical variables) with random effects, accounting for child age, sex, and geographic clustering [33]. We derived difference-in-differences estimates (DDEs) of impact using fixed-effects regression models that assessed differences between the two study groups over time [34]. The difference-in-differences method relies on comparing the difference in changes (i.e., differences) between baseline and follow-up in the intervention and control groups. Even though there are other methods of attributing the impact of interventions, the difference-in-differences method with randomization allows a best estimate of the impact attributable to the program by taking into account the changes in outcomes that may occur among the non-beneficiary population as a result of non-program factors such as secular trends, other types of programs, or climate-related or other types of shocks that may have independent positive or negative impacts on the study’s main outcomes. Difference-in-differences impact analyses have been widely used in health and nutrition impact evaluations [34].

We present intent-to-treat DDEs adjusting for geographic clustering, infant age, and gender. Using a subset of five items adapted from Reynolds [35] that were collected in the endline survey, we conducted a robustness analysis for EBF to test for social desirability bias, i.e., tendency of respondents to respond so as to be viewed favorably by others. Data analysis was performed using STATA 13; a statistical analysis plan was developed prior to endline data collection and discussed with the funder and program implementers. Although the prespecified plans assumed a one-sided test, we conducted analyses using two-sided tests to allow for the possibility of uncovering unexpected effects.

## Results

### Trial Flow

**Fig 2:**
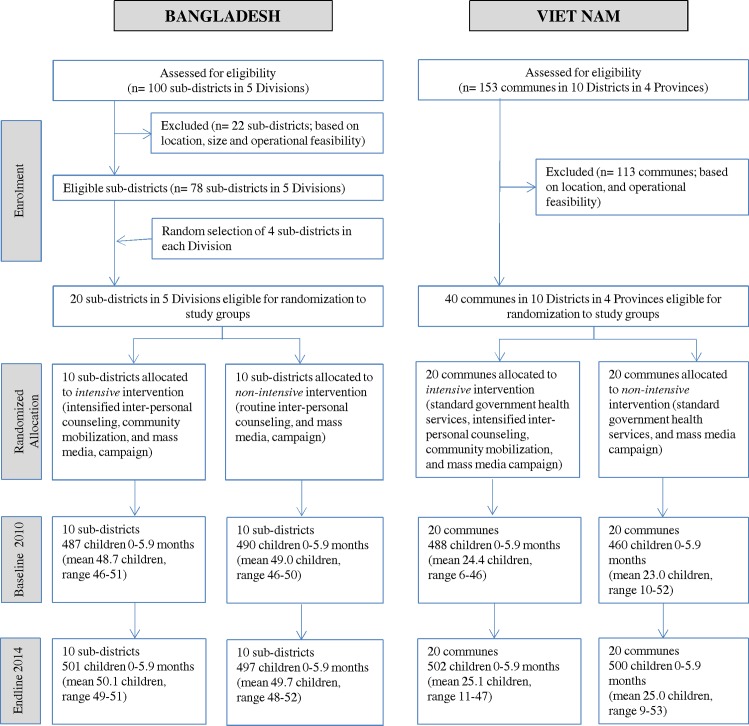
CONSORT diagram.

No evaluation clusters were lost to follow-up (Fig 2); none crossed from non-intensive to intensive during implementation. In Bangladesh, cluster size varied little across clusters or over time; in Viet Nam, there was greater variability, reflecting commune population size.

### Sample Characteristics

Desired sample sizes were achieved at baseline (June 7–August 29, 2010, in Viet Nam; April 28–June 26, 2010, in Bangladesh) and endline (June 16–August 30, 2014, in Viet Nam; April 20–June 23, 2014, in Bangladesh) in both countries. In Bangladesh, 977 households were surveyed at baseline, and 998 households at endline. In Viet Nam, achieved sample sizes were 948 at baseline and 1,002 at endline.

The two intervention groups were well-balanced for characteristics potentially associated with intervention effects in both countries (e.g., infant age and sex, household economic status, and maternal education and nutrition). In Bangladesh, there were small differences at baseline in maternal age, maternal employment, and household headship, and a 10.0-pp difference in ownership of agricultural land (Table 2). In Viet Nam, a higher mean level maternal mental stress and slightly higher mean level of mild food insecurity were seen in the non-intensive group at baseline.

**Table 2:** Selected characteristics of the study sample in Bangladesh and Viet Nam at baseline and endline.

Category	Characteristics	Bangladesh						Viet Nam					
		Baseline			Endline			Baseline			Endline		
		Intensive (*n =* 487)	Non-intensive (*n =* 490)	*p*-Value	Intensive (*n =* 501)	Non-intensive (*n =* 497)	*p*-Value	Intensive (*n =* 488)	Non-intensive (*n =* 460)	*p*-Value	Intensive (*n =* 502)	Non-intensive (*n =* 500)	*p*-Value
**Maternal characteristics**	**Mean age, years**	25.59	24.54	0.056	24.77	24.19	0.068	27.16	27.14	0.722	28.01	27.70	0.192
	**Women’s occupation, percent**												
	Housewife	96.10	94.69	0.455	75.25	85.11	0.009	13.93	10.00	0.922	11.75	14.00	0.476
	Farmer	—	—	—	0.80	0.20	0.110	51.64	51.52	0.423	38.65	33.60	0.498
	Self-employed	0.62	0.82	0.723	19.76	10.06	0.005	14.34	14.35	0.807	14.54	16.80	0.394
	Salary employee	1.44	0.20	0.061	1.80	1.41	0.595	19.06	23.04	0.157	33.86	34.40	0.144
	Others	1.85	4.29	0.126	2.40	3.22	0.366	1.02	1.09	NA	1.20	1.20	NA
	**Work outside the home, percent**	2.26	1.22	0.359	1.8	1.8	0.988	20.90	23.26	0.381	13.15	11.20	0.346
	**Education, percent**												
	No schooling	24.44	21.43	0.263	13.17	11.87	0.818	2.41	1.23	NA	0.60	0.20	NA
	Primary school	29.57	27.14	0.452	30.34	31.19	0.142	10.50	12.35	0.783	8.57	8.40	0.752
	Secondary school	39.63	42.45	0.359	45.31	40.64	0.020	52.52	49.18	0.802	40.44	37.60	0.394
	High school	5.75	7.96	0.102	8.98	15.49	0.103	19.69	23.46	0.342	30.08	29.60	0.989
	College or higher	0.62	1.02	NA	2.20	0.80	NA	14.88	13.79	0.927	20.32	24.20	0.416
	**Mean body mass index, kg/m** ^**2**^	20.48	20.59	0.779	21.30	21.34	0.933	20.45	20.36	0.956	21.25	21.17	0.842
	**Underweight** ^**1**^ **, percent**	24.90	26.34	0.623	19.68	19.43	0.940	20.08	22.44	0.676	12.95	11.40	0.355
	**Mean mental stress score**	6.62	6.11	0.582	4.34	5.85	0.002	4.23	5.33	0.168	3.78	4.09	0.083
	**Mental stress score ≥ 7, percent**	43.53	41.63	0.684	21.96	35.21	0.005	26.64	31.52	0.096	16.93	19.60	0.192
**Child characteristics**	**Mean age, months**	3.39	3.30	0.286	2.80	2.78	0.832	3.55	3.53	0.997	3.38	3.39	0.935
	**Female, percent**	50.10	50.20	0.959	54.29	51.51	0.277	45.70	48.91	0.322	46.81	44.00	0.371
**Household characteristics**	**Female household head, percent**	13.35	7.35	0.024	13.17	9.26	0.041	16.60	20.49	0.127	17.73	20.60	0.248
	**Household food security** ^**2**^ **, percent**												
	Food secure	68.79	69.80	0.880	85.83	80.48	0.081	70.08	65.14	0.151	81.08	80.80	0.855
	Mildly food insecure	8.01	6.33	0.925	4.59	5.23	0.600	12.09	15.90	0.035	7.97	9.20	0.422
	Moderately food insecure	11.09	11.22	0.495	5.19	6.44	0.118	14.75	14.16	0.682	8.96	7.60	0.556
	Severely food insecure	12.11	12.65	0.873	4.39	7.85	0.099	3.07	4.79	NA	1.99	2.40	NA
	**Household assets**												
	Ownership of house, percent	94.87	93.88	0.651	96.21	95.17	0.354	39.34	38.91	0.892	80.08	79.20	0.730
	Ownership of agricultural land, percent	49.28	39.80	0.013	50.30	45.27	0.148	71.72	73.04	0.649	71.91	72.40	0.863
	Ownership of garden, percent	27.52	33.06	0.442	28.14	36.22	0.089	66.05	61.66	0.160	73.90	73.20	0.800
	Mean total number of durable goods	69.70	70.20	0.969	73.89	71.25	0.657	18.58	17.95	0.404	21.78	22.27	0.557
	Mean total number of livestock	7.44	6.19	0.110	6.41	5.60	0.062	19.74	16.30	0.353	28.12	24.67	0.446
	Mean socioeconomic index^3^	0.05	0.15	0.522	0.09	0.10	0.896	−0.27	−0.27	0.811	0.20	0.30	0.397

*p*-Values obtained from model adjusted for clustering effect at commune and district level. “NA” indicates *p*-values not available due to too few numbers in the cells.

^1^Body mass index < 18.5 kg/m^2^.

^2^Household food security was measured using FANTA/USAID’s Household Food Insecurity Access Scale.

^3^A socioeconomic index was constructed using principal components analysis with variables on ownerships and assets. It is a standardized score with mean = 0 and standard deviation = 1.

### Impact of Intervention on EBF and Other Breastfeeding Practices

**Fig 3:**
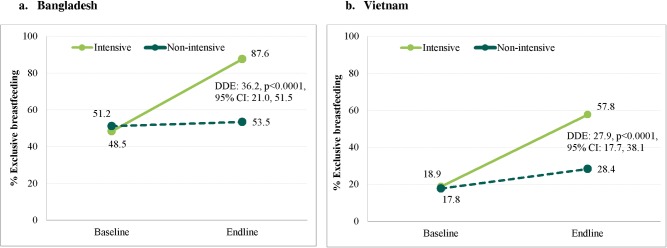
Prevalence of exclusive breastfeeding practices by program and survey round in Bangladesh and Viet Nam. Difference-in-differences estimate (DDE) between baseline and endline, adjusted for clustering at commune and district level, child’s age, and gender for (A) Bangladesh and (B) Viet Nam. DDE, difference-in-differences estimate.

DDEs (Fig 3) show that there were greater increases between baseline and endline in the proportion of women practicing EBF in the intensive group than in the non-intensive group, both in Bangladesh (36.2 pp, 95% CI 21.01–51.46, *p <* 0.001) and Viet Nam (27.9 pp, 95% CI 17.74–38.07, *p <* 0.001) (Fig 3). The proportion of women practicing EBF in the intensive group increased from 48.5% to 87.6% in Bangladesh, and from 18.9% to 57.8% in Viet Nam. There was no significant increase in the proportion of women practicing EBF in the non-intensive group over time in Bangladesh, but an increase of 10.0 pp in Viet Nam.

**Fig 4:**
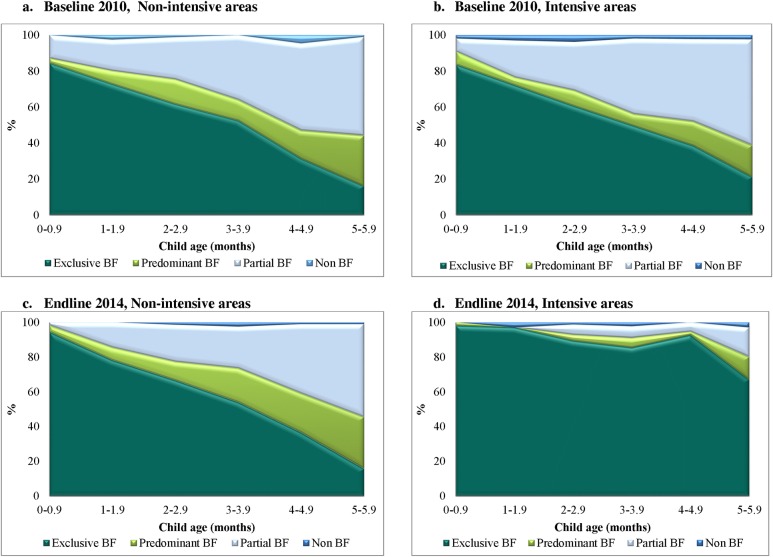
Patterns of breastfeeding (exclusive, predominant, partial, and non-breastfeeding), by child’s age, intervention program, and survey round in Bangladesh. (A) Baseline, 2010, non-intensive areas. (B) Baseline, 2010, intensive areas. (C) Endline, 2014, non-intensive areas. (D) Endline, 2014, intensive areas. BF, breastfeeding.

**Fig 5:**
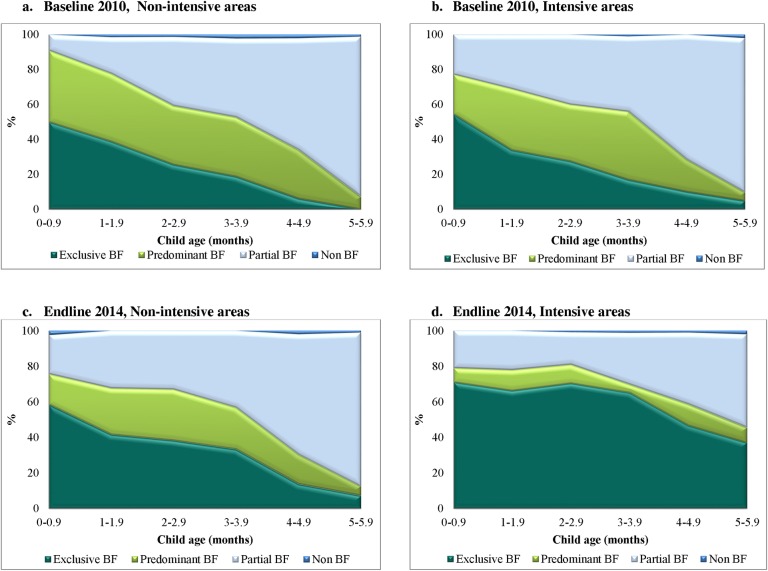
Patterns of breastfeeding (exclusive, predominant, partial, and non-breastfeeding), by child’s age, intervention program, and survey round in Viet Nam. (A) Baseline, 2010, non-intensive areas. (B) Baseline, 2010, intensive areas. (C) Endline, 2014, non-intensive areas. (D) Endline, 2014, intensive areas. BF, breastfeeding.

In the intensive group in Bangladesh, effects on EBF were primarily achieved through replacement of partial breastfeeding with EBF (Fig 4). In Viet Nam, effects on EBF were primarily achieved through replacement of predominant breastfeeding with EBF (Fig 5). In Bangladesh, DDEs were also statistically significant for EIBF (16.7 pp, 95% CI 2.78–30.57, *p* = 0.021) and feeding of prelacteals during the first 3 d after birth (−49.3 pp, 95% CI −65.60 to −32.92, *p <* 0.001); in Viet Nam, DDEs were statistically significant for feeding of prelacteals during the first 3 d after birth (−18.8 pp, 95% CI −30.86 to −6.15, *p* = 0.003), formula use during the first 3 d after birth (−22.4 pp, 95% CI −33.53 to −10.67, *p <* 0.001), and bottle feeding in the first 6 mo (−13.0 pp, 95% CI −20.26 to −5.54, *p <* 0.001) (Table 3).

**Table 3:** Main results for exclusive breastfeeding and other breastfeeding behaviors.

IYCF Indicator	Baseline Survey 2010			Endline Survey 2014			DDE, Percentage Points (95% CI)			
	Intensive Percent	Non-intensive Percent	*p-*Value	Intensive Percent	Non-intensive Percent	*p-*Value	Pure DDE^1^	*p*-Value	Adjusted DDE^2^	*p-*Value
**Bangladesh**	*n =* 487	*n =* 490		*n =* 501	*n =* 497					
EIBF (within 1 h of birth)	63.66	62.65	0.820	94.21	76.66	<0.001	16.50 (2.68, 30.42)	0.022	16.70 (2.78, 30.57)	0.021
EBF among children <6 mo	48.46	51.22	0.495	87.62	53.52	<0.001	36.90 (21.93, 51.80)	<0.001	36.20 (21.01, 51.46)	<0.001
EBF among children 0–0.9 mo	84.21	84.00	0.979	98.18	94.55	0.308	3.43 (−13.77, 20.62)	0.681	4.00 (−13.98, 21.98)	0.647
EBF among children 1–5.9 mo	45.43	47.50	0.537	86.32	48.42	<0.001	39.97 (24.46, 55.48)	<0.001	40.17 (24.98, 55.36)	<0.001
Predominant breastfeeding among children <6 mo	14.99	10.00	0.106	4.79	17.10	<0.001	−17.30 (−25.18, −9.42)	<0.001	−17.1 (−25.11, −9.18)	<0.001
Fed any prelacteals immediately after birth	53.18	36.12	0.004	3.79	23.54	<0.001	−36.80 (−48.95, −24.66)	<0.001	−36.90 (−49.01, −24.79)	<0.001
Fed any prelacteals during the first 3 d	51.33	23.88	0.001	9.78	31.59	<0.001	−49.20 (−65.56, −32.98)	<0.001	−49.30 (−65.60, −32.92)	<0.001
Fed water during the first 3 d	6.37	5.10	0.615	0.60	3.82	<0.001	−4.50 (−11.32, 2.35)	0.187	−4.50 (−11.33, 2.36)	0.186
Bottle feeding in the first 6 mo	13.96	14.69	0.874	3.81	14.55	<0.001	−10.1 (−20.23, 0.22)	0.053	−9.90 (−20.23, 0.37)	0.058
**Viet Nam**	*n =* 488	*n =* 460		*n =* 502	*n =* 500					
EIBF (within 1 h of birth)	60.04	57.39	0.408	53.18	40.60	0.001	9.90 (−0.34, 20.21)	0.058	10.00 (−1.25, 21.40)	0.072
EBF among children <6 mo	18.85	17.82	0.680	57.76	28.40	<0.001	28.30 (17.72, 38.96)	<0.001	27.90 (17.74, 38.07)	<0.001
EBF among children 0–0.9 mo	54.55	50.00	0.765	71.05	58.54	0.320	7.97 (−28.20, 44.10)	0.658	6.74 (−29.08, 42.56)	0.705
EBF among children 1–5.9 mo	17.17	16.21	0.700	56.68	25.71	<0.001	30.02 (19.88, 40.15)	<0.001	29.82 (19.79, 39.86)	<0.001
Predominant breastfeeding among children <6 mo	24.79	28.04	0.257	9.36	19.40	<0.001	−6.80 (−13.23, −0.35)	0.039	−6.90 (−13.37, −0.84)	0.033
Fed any prelacteals immediately after birth	54.62	59.28	0.158	49.20	67.40	<0.001	−13.50 (−24.47, −2.62)	0.016	−13.60 (−25.03, −2.10)	0.017
Fed any prelacteals during the first 3 d	48.05	55.29	0.026	46.22	72.20	<0.001	−18.70 (−30.47, −7.02)	0.002	−18.80 (−30.86, −6.15)	0.003
Fed water during the first 3 d	24.59	26.80	0.438	6.77	18.60	<0.001	−9.60 (−20.21, 0.97)	0.074	−9.70 (−21.25, 0.28)	0.078
Fed formula during the first 3 d	31.35	32.46	0.715	41.04	64.60	<0.001	−22.50 (−32.94, −11.97)	<0.001	−22.40 (−33.53, −10.67)	<0.001
Bottle feeding in the first 6 mo	28.89	30.43	0.604	17.72	32.60	<0.001	−13.30 (−20.29, −6.37)	<0.001	−13.00 (−20.26, −5.54)	0.001

^1^DDE between baseline and endline adjusted for clustering effect at commune and district level only. The intra-cluster correlations for Bangladesh were 0.1018 and 0.0755 for EIBF and EBF, respectively, and for Viet Nam were 0.1009 and 0.0739 for these two outcomes, respectively.

^2^DDE between baseline and endline adjusted for clustering effect at commune and district level, child’s age, and gender.

DDE, difference-in-differences estimate; EBF, exclusive breastfeeding; EIBF, early initiation of breastfeeding; IYCF, infant and young child feeding.

EBF in children under 1 mo of age did not differentially improve by intervention group in either country. Impacts in both countries were due to the differential improvements in EBF in favor of the intensive group among children 1–5.9 mo (Table 3).

### Intervention Exposure

In the 6 mo preceding the survey, mothers in Bangladesh reported receiving 6.6 visits by the IYCF worker (Pushti Kormi) and health volunteer (Shasthya Kormi) trained to deliver nutrition-related IPC as part of A&T, while mothers in Viet Nam visited the health facilities for counseling an average of two times (Table 4). Fidelity in terms of contacts with FLWs and volunteers was high in Bangladesh (85%–98%, depending on the type of FLW or volunteer), whereas use of the health facility in Viet Nam was lower (<50% reported visiting the A&T franchise facility [MTBT] in intensive areas). In both countries, the breastfeeding-related knowledge of FLWs and volunteers was higher in intensive compared to non-intensive areas. Attendance at a CM session ranged from 12% to 22% in the intensive group in Bangladesh. Maternal exposure to the TV spots was 61%–64% in Bangladesh, and did not differ between groups. Similarly, exposure to TV spots was 70% in Viet Nam, with no statistically significant differences between groups. As expected, awareness and exposure to the social franchise brand was significantly higher among individuals in the intensive than in the non-intensive group.

**Table 4:** Intervention exposure and use of program services at endline.

Country	Program Participation and Use of Services	Subcategory	Percent or Mean (Standard Deviation)		*p*-Value
			Intensive	Non-Intensive	
**Bangladesh**	**Interpersonal counseling**				
	Mother was visited at least once by the health volunteer (SS) (unaided recall)		85.83	25.96	<0.001
	Mother was visited at least once by the IYCF worker (PK) (unaided recall)		84.63	—	—
	Mother was visited at least once by the health volunteer (SS) (aided recall)		98.23	58.17	<0.001
	Mother was visited at least once by the IYCF worker (PK) (aided recall)		98.44	—	—
	Number of times visited by SS in the last 6 mo	All age groups	3.72 (2.77)	0.60 (1.57)	<0.001
		Children 0–1.9 mo	3.05 (2.56)	0.84 (1.95)	<0.001
		Children 2–3.9 mo	3.68 (2.84)	0.60 (1.52)	<0.001
		Children 4–5.9 mo	4.20 (2.76)	0.44 (1.27)	<0.001
	Number of times visited by PK in the last 6 mo	All age groups	2.87 (2.02)	—	—
		Children 0–1.9 mo	2.16 (2.05)	—	—
		Children 2–3.9 mo	2.60 (1.83)	—	—
		Children 4–5.9 mo	3.56 (1.95)	—	—
	**Social mobilization**				
	Attended a popular theater show on IYCF in the last 1 y		12.18	0.40	<0.001
	Number of popular theater shows attended in the last 1 y		0.16 (0.46)	0.00 (0.06)	<0.001
	Attended a video show on IYCF in the last 1 y		21.96	1.01	<0.001
	Number of video shows attended in the last 1 y		0.30 (0.97)	0.02 (0.37)	0.001
	**Mass media**				
	Ever seen TV spot 1: “Early Initiation”		64.07	60.56	0.454
	Ever seen TV spot 2: “Insufficient Milk”		61.48	54.53	0.165
	Ever seen either TV spot 1 or TV spot 2		66.87	64.39	0.590
	Ever seen both TV spot 1 and TV spot 2		58.68	50.70	0.116
	Recalled at least one message from TV spot 1		91.28	87.71	0.078
	Recalled at least two messages TV spot 1		80.06	71.43	0.010
	Recalled at least one message from TV spot 2		90.26	86.35	0.173
	Recalled at least two messages from TV spot 2		82.14	73.80	0.035
	**FLW performance**				
	BF knowledge score for SS (out of 9)		7.68 (1.01)	6.17 (1.22)	<0.001
	BF knowledge score for PK (out of 9)		7.76 (1.03)	—	—
**Viet Nam**	**Interpersonal counseling**				
	Ever seen MTBT logo		91.24	61.00	<0.001
	Ever heard the name of MTBT		77.29	28.20	<0.001
	Ever seen an invitation card		49.40	3.80	<0.001
	Ever received an invitation card to go to MTBT		39.04	1.00	<0.001
	Have been to MTBT		47.61	1.20	<0.001
	Number of times visited MTBT in the last 6 mo	All age groups	1.74 (1.38)	0.50 (0.55)	0.030
		Children 0–1.9 mo	1.56 (1.39)		
		Children 2–3.9 mo	1.82 (1.38)		
		Children 4–5.9 mo	1.73 (1.39)		
	**Mass media**				
	Ever seen TV spot “No Water”		70.92	67.40	0.071
	Recalled at least one message		88.76	86.94	0.568
	Recalled at least two messages		66.57	61.42	0.149
	Recalled specific messages	Exclusive breastfeeding for children <6 mo	73.03	74.48	0.803
		Breastmilk has enough water	43.26	37.98	0.056
		No water for children <6 mo	33.15	29.08	0.164
		Breastmilk has enough nutrients	48.88	43.92	0.156
		No formula for children <6 mo	16.88	10.98	0.005
	**FLW performance**				
	Breastfeeding knowledge (out of 9)		8.33 (0.63)	7.18 (1.47)	<0.001
	Communication skills (out of 22)		15.96 (2.71)	9.52 (3.22)	<0.001
	Technical skills (out of 11)		4.74 (1.51)	2.77 (1.09)	<0.001

FLW, frontline worker; IYCF, infant and young child feeding; MTBT, Mat Troi Be Tho; PK, Pushti Kormi; SS, Shasthya Sebika.

### Accuracy of Main Outcome Measures

We measured social desirability in both countries to assess, and account for, potential biases in our main impact estimates for reported breastfeeding practices (S1 Text). We found evidence of a social desirability bias for EBF in Viet Nam, but not in Bangladesh (S3 Fig). There was no evidence of this bias for other breastfeeding outcomes (S4 Fig). In Viet Nam, as social desirability scores for EBF increased, reported EBF increased in both groups, but more so in the intensive group. After adjusting for this differential increase, the impact estimate for EBF in Viet Nam remained strong and statistically significant (DDE 15.2 pp, *p* = 0.008). In addition, based on the premise that diarrhea could potentially be lower in the EBF group, we cross-checked EBF self-reports and infant diarrhea and found that the prevalence of diarrhea was indeed lower among EBF infants than among non-EBF infants (3.2% versus 5.0% in Bangladesh, *p =* 0.044, and 3.7% versus 9.7% in Viet Nam, *p <* 0.001).

## Discussion

An at-scale behavior change program that focused on integrating intensified IPC, MM, and CM (the A&T intensive intervention) had a greater impact on breastfeeding practices, especially EBF than MM with standard nutrition counseling (the A&T non-intensive intervention), within the context of national advocacy to create a supportive environment for optimal feeding practices. In Bangladesh and Viet Nam, the proportion of women who reported practicing EBF in the previous 24 h among children <6 mo was 36 pp and 28 pp higher, respectively, in the intensive compared to the non-intensive intervention areas. In Bangladesh, where intensified IPC was delivered through repeated home visits at critical ages combined with CM, the proportion of women practicing EBF at endline was 88% in the intensive group (from a baseline prevalence of 49%). In Viet Nam, the proportion of women practicing EBF reached 58% (from a low 19% at baseline) through a facility-based delivery model for IPC that was built on principles of social franchising and helped strengthen the health system’s capacity to deliver counseling. These impacts were seen despite positive changes in household economic conditions, maternal education, and occupation that in many settings in Asia are associated with lower rates of EBF [36].

The higher EBF prevalence in the intensive compared to the non-intensive group at endline in Bangladesh and Viet Nam is comparable to results from systematic reviews of breastfeeding promotion interventions [8,9]. By contrast with previous literature, the magnitude of the effect we report was achieved in a large at-scale program. In Bangladesh the intensive approach was implemented in 50 of 493 rural sub-districts, 20 of which were in the evaluation. Based on program monitoring, BRAC reported conducting 2.2 million home visits focused on the promotion of EBF to mothers of infants 0–5.9 mo old between December 2011 and February 2014. In Viet Nam, a total of 781 A&T franchises in 15 of 63 provinces (four of which were in the evaluation) were operational at endline, and there were 1.1 million counseling contacts with mothers of infants 0–5.9 mo of age at health facilities between January 2012 and December 2014. The Bangladesh results are generalizable to other program models that rely on incentivized community volunteers and/or skilled FLWs conducting home-based or community IPC and CM, and suggest that intensifying and strengthening contacts and linking them with MM could have significant benefits [37,38]. The results for Viet Nam are applicable to other countries where primary health care utilization is high, MM reach is almost universal, and facility-based platforms can be used to deliver preventive and curative health care. In Viet Nam, the four provinces selected for evaluation were representative of the A&T program areas in terms of geographic regions (north, south, and central). Compared to the rest of the country and parts of the program areas, however, they were more rural and had a higher level of stunting. Similarly, in Bangladesh, the program was operational primarily in rural areas. Therefore, the results are primarily generalizable to rural areas served by government health services (in countries like Viet Nam) and rural areas in countries like Bangladesh.

The A&T model for improving breastfeeding at scale was initiated in 2008, and developed in 2009–2010, based on prior experience [18]. Much of the evidence on the impacts of breastfeeding [1], and on elements necessary to assure scale up of breastfeeding programs using multiple platforms and data-driven coordinated efforts [12], was available only after the initial development of the A&T model. This rigorous evaluation of a complex intervention now deeply strengthens the programming evidence base for improving breastfeeding, and supports the data-driven and adaptive approach that has been recommended but not always put into practice. The A&T model, thus, provides guideposts for developing a practical and well-coordinated approach that links PA, strengthening of systems for implementation at scale, and the use of data from formative research, monitoring, and evaluation.

The plausibility of our findings are strengthened by the findings from a process evaluation [21,39,40] on service delivery and intervention exposure. Interventions were implemented largely as designed in both countries [21,40,41], but demand-side constraints to use of facilities were a challenge in Viet Nam [39]. In Bangladesh, intensified IPC was delivered well by FLWs, who were incentivized and monitored to cover all targeted households; achieved coverage was very high. In Viet Nam, high-quality counseling was established in the intensive group compared to the non-intensive group [21]; since reach was dependent on mothers visiting the health facilities, and subject to demand-side constraints, the model achieved lower coverage [39] than the outreach-based model in Bangladesh. MM reached a substantial proportion of the population in both settings, and we saw evidence of shifts in some breastfeeding practices in the non-intensive group as well. It is likely the MM played multiple roles and worked synergistically with the IPC, but our evaluation design does not tease these apart.

A challenge in assessing impact on feeding practices is the use of recall-based self-reported measures. Our study was subject to this challenge, particularly because the intervention included a MM component that provided regular reminders of recommended behaviors. Although we did not collect data on lactational amenorrhea, we did collect information on infant diarrhea (reported by the mothers). We compared differences in diarrhea prevalence between groups, finding, as expected, that there was lower prevalence of diarrhea among EBF infants compared to non-EBF infants. We also assessed the role of social desirability in relation to the main impact indicators [35]. In Bangladesh, the results were unaffected by respondents’ desire for social approval. In Viet Nam, it appears that respondent desire for social approval had some influence on reporting (S1 Text), which was differential between intervention groups, but strong and significant intervention effects were still demonstrated after accounting for this reporting bias.

In both countries, the evaluation was restricted to a significantly smaller geographic area than the true coverage of the program. In order to assess the possibility that there was a differential quality of service delivery due to the non-blinded nature of the intervention, we randomly assessed service delivery in a subset of intensive areas that were not included in the impact evaluation. We found no evidence of differential quality of service delivery in either country, further supporting the external validity of the evaluation findings to the at-scale program in both countries.

From the perspective of understanding the scaling up of a successful breastfeeding program, and the special efforts made by this initiative to ensure delivery of a comprehensive set of interventions at large scale, an analysis focused on the scaling up of this program identified the presence of several critical elements for scaling up [22]. Specifically, the program appears to have successfully included multiple elements deemed necessary for successful scale-up, including the following: (1) a vision for impact on breastfeeding; (2) finding the right combination of interventions and operational contexts in the two countries; (3) having access to adequate, stable, and flexible financing to stay adaptive and goal-focused; (4) actively engaging champions and alliances via PA; (5) using multiple pathways to scaling up—expanding and strengthening the capacities (increasing FLWs in Bangladesh and establishing counseling rooms within facilities in Viet Nam); and (6) including adequate learning through the use of data and learning. Much of what occurred in the context of this program also reflects elements of the complex adaptive health care systems framework [42,43]. Looking forward, assessments of the sustainability of these actions at scale will be needed to understand the extent to which these investments led to a sustained legacy focus on infant feeding in the context of the BRAC program in Bangladesh and the health system in Viet Nam.

Given that the A&T intervention was a large-scale intervention involving IPC, MM, and CM, there was some potential for between-group contamination. In Bangladesh, there was no between-group contamination because the sub-districts were administratively separated for implementation, and we verified the lack of contamination through routine interactions with the program implementation team. In Viet Nam, since the communes were situated within districts that were in turn located inside the provinces chosen for the overall A&T program, there was potential for some between-group contamination. We assessed this contamination informally in 2013 and ascertained that some content-related overlap had occurred in refresher training of health workers. Given that between-group contamination is likely to have dampened intervention effects, overall intervention effects may have been even higher than those seen.

This study was not designed to assess the impact of MM alone on breastfeeding practices, because the media campaign was implemented nationwide. Similarly, our design did not allow us to isolate the contributions of the nationwide PA activities, which aimed to create an enabling environment for implementation and scale-up of IYCF programs in the country. Our impact estimates may thus underestimate the full potential of such a multipronged intervention because the evaluation lacks a “pure control” area with no MM or national/provincial PA. Lastly, the EBF indicator itself (EBF in the previous 24 h), though currently recommended by WHO, has limitations. Most notably, it is reflective only of the previous day’s practice and does not inform about usual or continued breastfeeding practices over time. A study of the dynamics of EBF in Peru showed that mothers switched back and forth between exclusive and predominant breastfeeding during the first 6 mo of the child’s life [44]. The possibility of this practice is also supported by smaller scale qualitative research in our process evaluations [45,46]. Although there is some evidence that the EBF indicator is accurate [47], further research is needed to develop stronger and more reliable indicators of EBF, beyond the 24-h recall-based indicator that is currently used.

## Conclusion

Evidence has been mounting on the positive impacts of breastfeeding on a range of maternal and child health outcomes in diverse settings [16,17], and yet progress on improving breastfeeding practices is held back globally [2], possibly because policies and interventions to date have not embraced full-scale efforts to deliver integrated, tailored, and well-coordinated strategies [12]. In this global health context, this study fills an important gap in the public health literature [11] on the impact of delivering a critically important child survival, nutrition, and human development intervention at scale. Using rigorous cluster-randomized evaluation designs, it shows that comprehensive behavior change strategies implemented at scale, under real-life conditions, and delivered through outreach-based (Bangladesh) and facility-based (Viet Nam) platforms have strong and significant impacts on breastfeeding practices. Strategies that combine intensive IPC with MM campaigns, CM, and advocacy are more effective than standard counseling with less-intensive accompanying strategies. Our study demonstrates the impacts of recommended complex adaptive systems-based approaches to programming for scaling up combined interventions to improve breastfeeding in low- and middle-income countries [10]. We conclude that investments in combined, coordinated, and data-driven strategies, especially those that intensify counseling and are supported by MM, CM, and PA, are recommended for replication in similar contexts and for sustained implementation in Bangladesh and Viet Nam.

## Supporting Information

S1 Checklist CONSORT 2010 checklist of information to include when reporting a cluster-randomized trial. (DOCX)

S2 Checklist STROBE Statement—checklist of items that should be included in reports of observational studies. (DOC)

S1 Data Data for Bangladesh. (DTA)

S2 Data Data for Viet Nam. (DTA)

S1 Fig Program scale in Bangladesh. (JPG)

S2 Fig Program scale in Viet Nam. (JPG)

S3 Fig Social desirability scores and exclusive breastfeeding, by program group. (TIF)

S4 Fig Social desirability scores and early initiation of breastfeeding, by program group. (TIF)

S5 Fig CONSORT diagram. (PDF)

S1 IRB Institutional review board approval from the Bangladesh Medical Research Council. (PDF)

S2 IRB Institutional review board approval from the Vietnam Union of Science and Technology Associations. (PDF)

S3 IRB Institutional review board approval from the International Food Policy Research Institute. (PDF)

S1 Table Key components of the Alive & Thrive impact evaluation surveys. (DOCX)

S1 Text Assessment of social desirability bias. (DOCX)

S2 Text Survey protocol for impact evaluation and statistical analysis—Bangladesh. (DOCX)

S3 Text Survey protocol for impact evaluation and statistical analysis—Viet Nam. (DOCX)

S4 Text Measurement, learning, and evaluation impact analysis plan. (DOCX)

## Abbreviations

A&T: Alive & Thrive
CM: community mobilization
DDE: difference-in-differences estimate
EBF: exclusive breastfeeding
EIBF: early initiation of breastfeeding
FLW: frontline worker
ICC: intra-class correlation
IPC: interpersonal counseling
IYCF: infant and young child feeding
MM: mass media
MTBT: Mat Troi Be Tho
PA: policy advocacy
pp: percentage points
