# Supplementary figures and images for: Impacts on Breastfeeding Practices of At-Scale Strategies That Combine Intensive Interpersonal Counseling, Mass Media, and Community Mobilization: Results of Cluster-Randomized Program Evaluations in Bangladesh and Viet Nam
Source: PLoS Med. 2016 Oct 25;13(10):e1002159. doi: 10.1371/journal.pmed.1002159 (PMC5079648; doi:10.1371/journal.pmed.1002159)

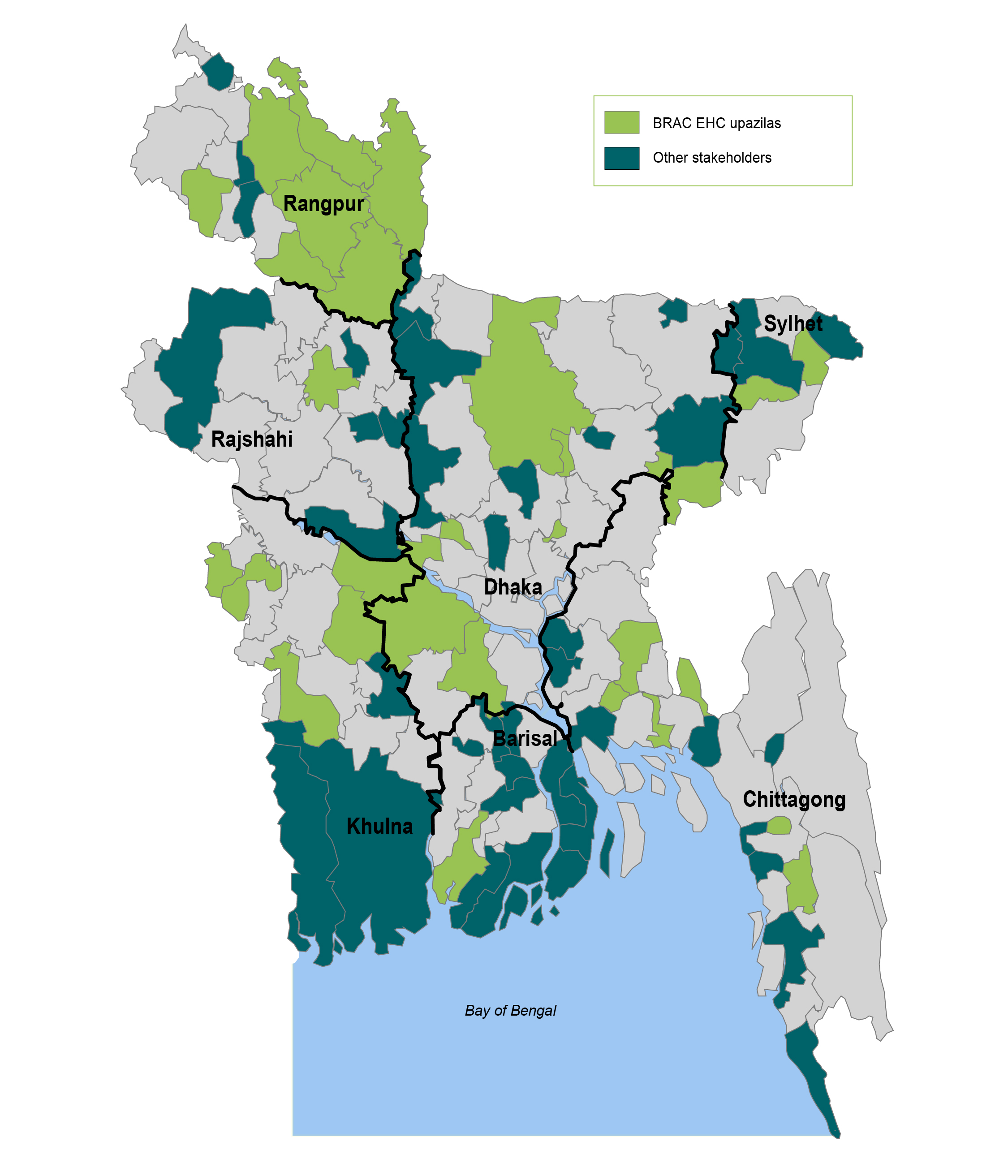

Supplement: S1 Fig — (JPG) [file pmed.1002159.s005.jpg]

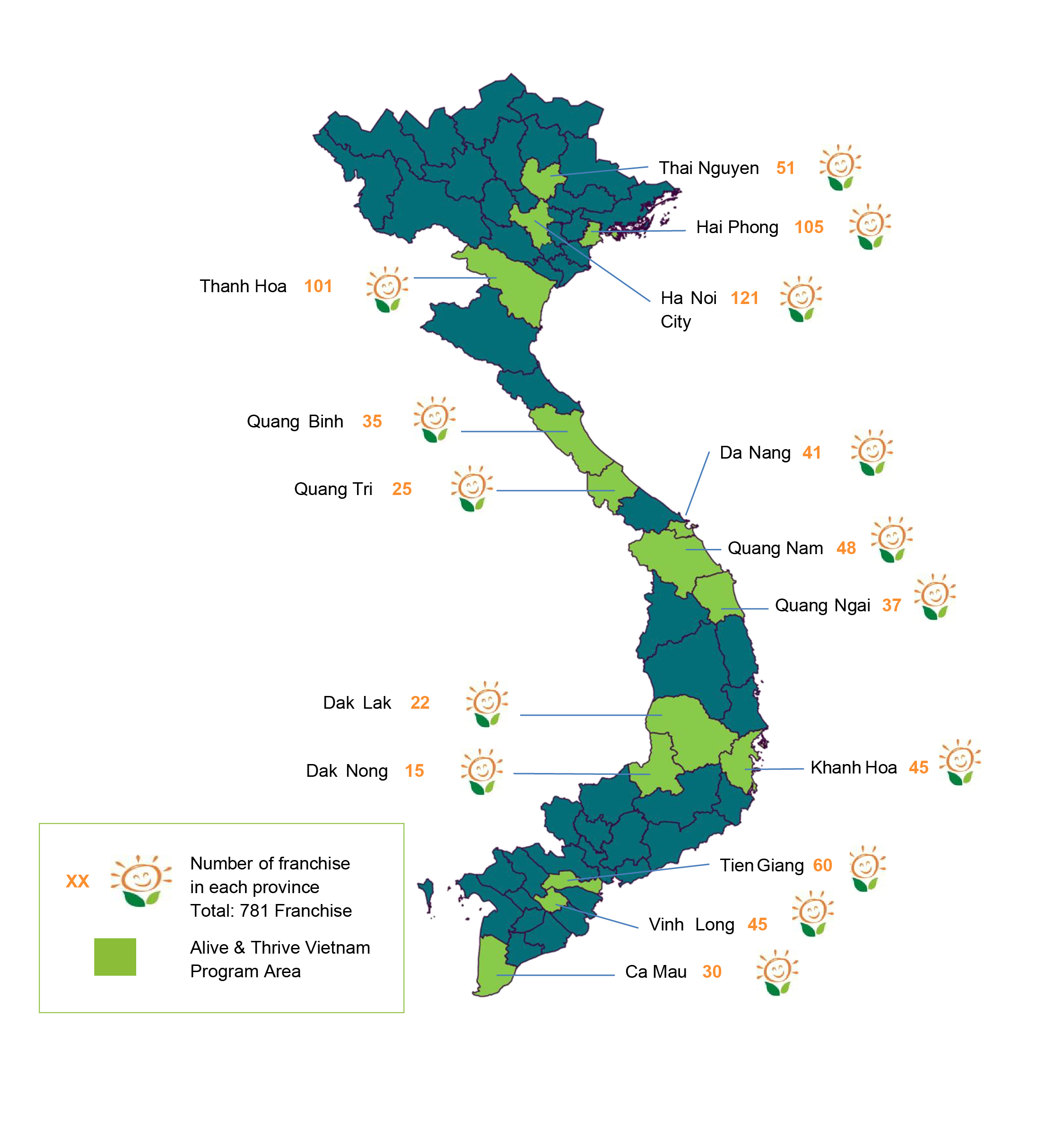

Supplement: S2 Fig — (JPG) [file pmed.1002159.s006.jpg]

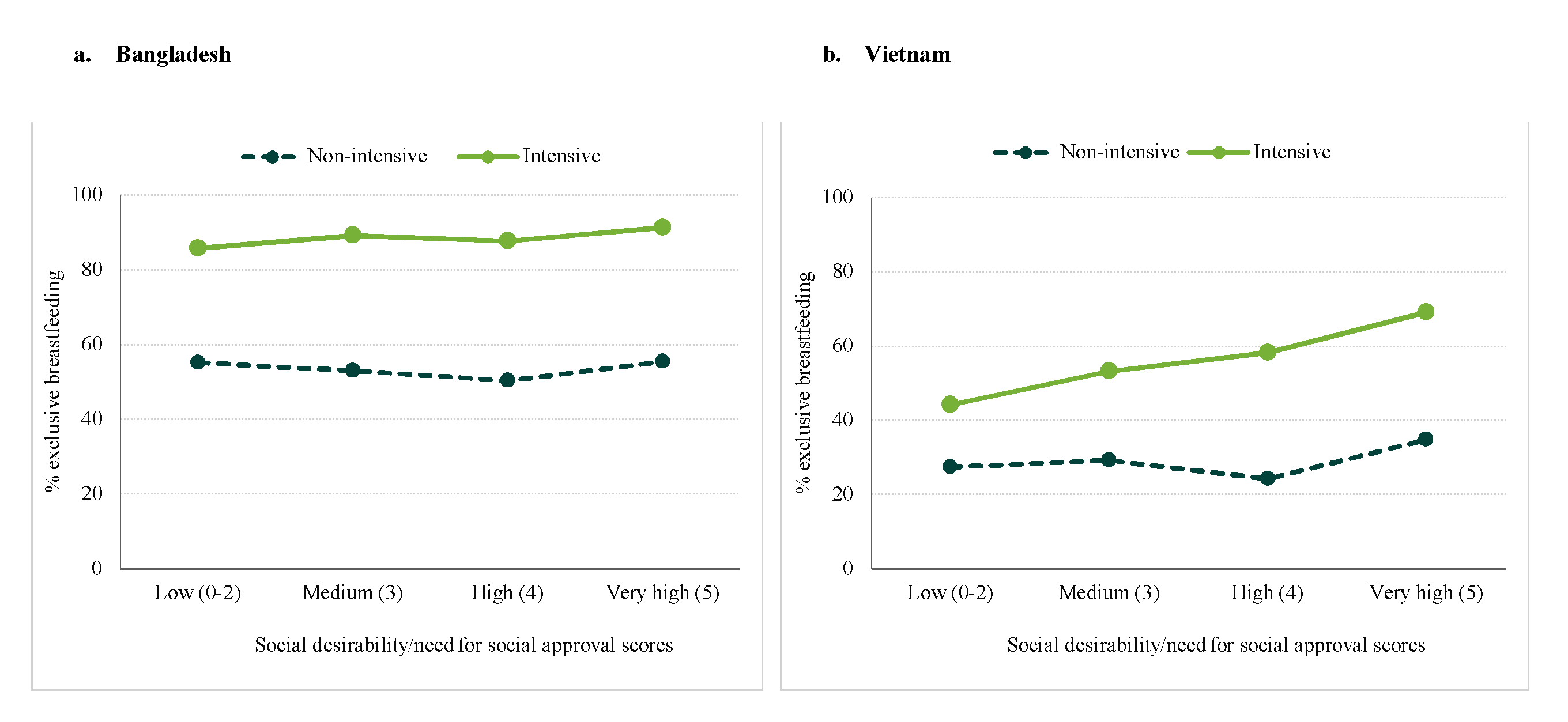

Supplement: S3 Fig — (TIF) [file pmed.1002159.s007.tif]

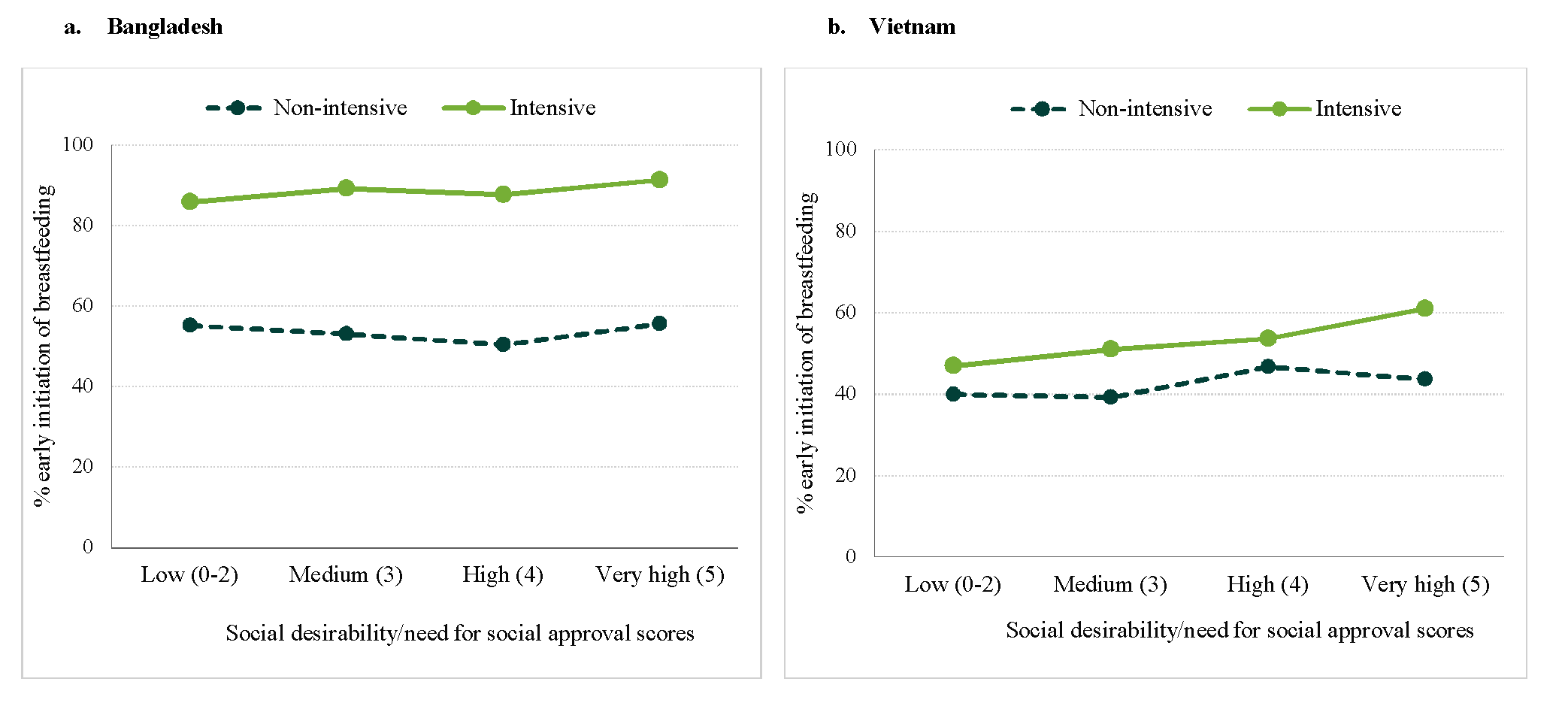

Supplement: S4 Fig — (TIF) [file pmed.1002159.s008.tif]

# Consort diagram

## BANGLADESH

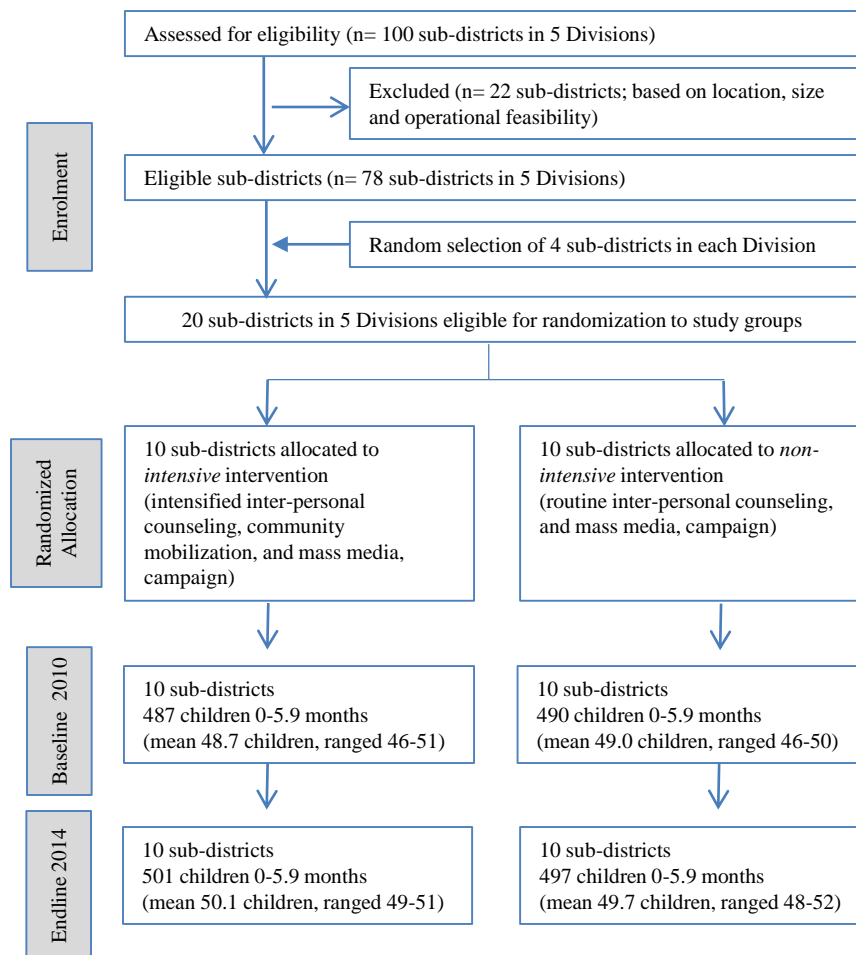

## VIETNAM

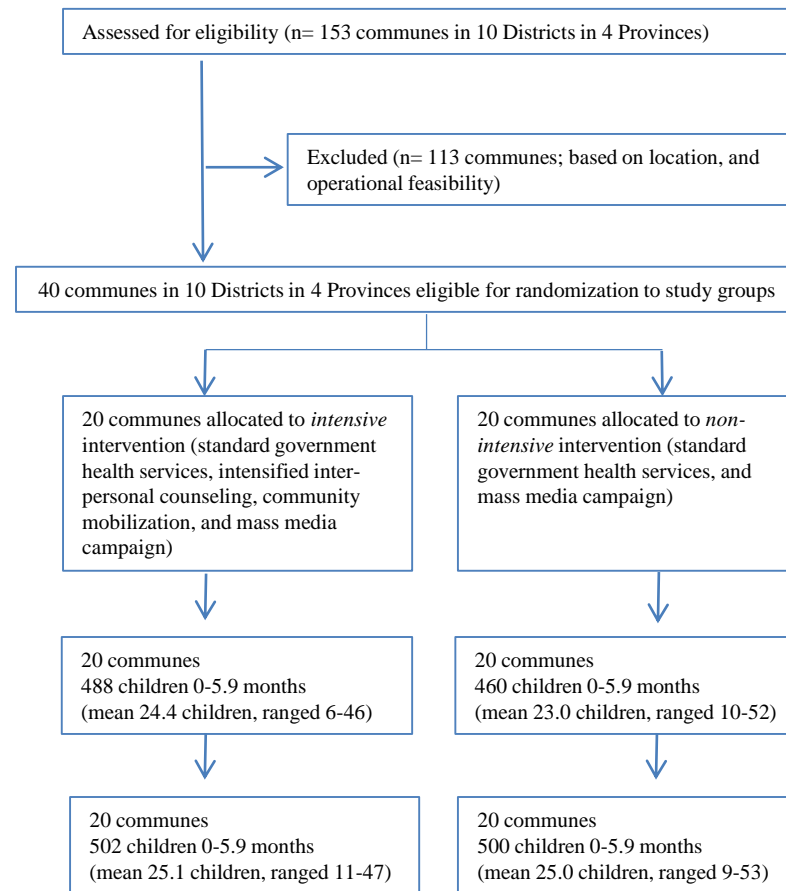

Supplement: S5 Fig — (PDF) [file pmed.1002159.s009.pdf]
