# Supplementary material for: Impacts on Breastfeeding Practices of At-Scale Strategies That Combine Intensive Interpersonal Counseling, Mass Media, and Community Mobilization: Results of Cluster-Randomized Program Evaluations in Bangladesh and Viet Nam
Source: PLoS Med. 2016 Oct 25;13(10):e1002159. doi: 10.1371/journal.pmed.1002159 (PMC5079648; doi:10.1371/journal.pmed.1002159)
Supplement: S1 IRB — (PDF) [file pmed.1002159.s010.pdf]

বাংলাদেশ চিকিৎসা গবেষণা পরিষদ
Bangladesh Medical Research Council
BMRC

Ref: BMRC/NREC/2007-2010/ 99 Date: 03-02-10

National Research Ethics Committee

Purnima Menon
International Food Policy Research Institute (IFPRI)
Washington, DC
USA.

Subject: Ethical Clearance

With reference to your application on the above subject, this is to inform you that your Research Proposal entitled **"Impact evaluation of behavior change communication and micronutrient supplementation interventions on infant and young child feeding (IYCF) practices and on childhood stunting and anaemia"** has been reviewed and approved by the National Research Ethics Committee (NREC).

You are requested to please note the following ethical guidelines as mentioned at page 2 (overleaf) of this memo-

(Prof. Harun-Ar-Rashid)
MD, MSc, MPH, PhD, FRCP Edin
Director

THE ETHICAL GUIDELINES TO BE FOLLOWED
BY THE PRINCIPAL/ CO-INVESTIGATORS

- ❒ The rights and welfare of individual volunteers are adequately protected.
- ❒ The methods to secure informed consent are fully appropriate and adequately safeguard the rights of the subjects (in the case of minors, consent is obtained from parents or guardians).
- ❒ The Investigator(s) assume the responsibility of notifying the National Research Ethics Committee (NREC) if there is any change in the methodology of the protocol involving a risk to the individual volunteers.
- ❒ To immediately report to the NREC if any evidence of unexpected or adverse reaction is noted in the subjects under study.
- ❒ This approval is subject to P.I.'s reading and accepting the BMRC ethical principles and guidelines currently in operation.
