# Supplementary material for: Impacts on Breastfeeding Practices of At-Scale Strategies That Combine Intensive Interpersonal Counseling, Mass Media, and Community Mobilization: Results of Cluster-Randomized Program Evaluations in Bangladesh and Viet Nam
Source: PLoS Med. 2016 Oct 25;13(10):e1002159. doi: 10.1371/journal.pmed.1002159 (PMC5079648; doi:10.1371/journal.pmed.1002159)
Supplement: S2 IRB — (PDF) [file pmed.1002159.s011.pdf]

TRANSLATION/BẢN DICH

VIETNAM UNION OF SCIENCE AND TECHNOLOGY ASSOCIATION (VUSTA)
CENTER FOR CREATIVE INITIATIVES IN HEALTH AND POPULATION (CCIHP)

OPPONENT COUNCIL IN BIOMEDICAL RESEARCH
No.: **0904**/HDPB-CCIHP
Re: Approving ethical issues in biomedical research

SOCIALIST REPUBLIC OF VIETNAM
Independence – Freedom – Happiness

Hanoi, 09 April 2010

APPROVAL OF OPPONENT COUNCIL IN BIOMEDICAL RESEARCH
CENTER FOR CREATIVE INITIATIVES IN HEALTH AND POPULATION

- Based on Decision No. 1504QD/CCIHP-2009 dated 15 April 2009 of Director of Center for Creative Initiatives in Health and Population on the establishment of Opponent Council in Biomedical Research (hereinafter referred as Opponent Council – OC) to approve ethical issues in biomedical research of themes/ projects;
- Based on regulations on organization and operation of Opponent Council of Center for Creative Initiatives in Health and Population;
- Based on meeting minutes of Opponent Council on 02 April 2010 (attached minutes);

Therefore, Opponent Council of Center for Creative Initiatives in Health and Population approves (permits) ethical issues in the research of topics/ projects:

- Name of the project: Impact evaluation of Alive and Thrive Franchise Interventions on Infant and Young Child Feeding (IYCF) Practices and on Childhood Stunting of the Rural Areas in Vietnam.
- Project director: Nguyen Duc Minh;
- Sponsor unit: Institute of Social and Medical Studies (ISMS)
- Place of research development: in 4 provinces: Dak Lak, Quang Ngai, Thanh Hoa and Vinh Long;
- Period of research: From 15 March 2010 to 15 August 2010

Approval (permit) date: April 9th, 2010

PRESIDENT OF THE COUNCIL	CENTER FOR CREATIVE INITIATIVES IN HEALTH AND POPULATION	SECRETARY OF THE COUNCIL
(Signed)	*(Sealed)*	*(Signed)*
**Vu Song Ha**		**Nguyen Thi Vinh**

IRB-CCIHP IORG number 0005789, FWA number 00014297

CERTIFICATION OF JUSTICE DIVISION OF CAU GIAY DISTRICT, HANOI CITY

This is certified that Mr. **Do Cong Khanh**, holding ID Card No.121476785, issued on March 09, 2007 by Public Security of Bac Giang Province, has appeared before me and subscribed his signature at the Justice Division of Cau Giay District.
Notarized No.: 117 Volume No.04/ SCT-CK
April 13th, 2010

CHIEF OF JUSTICE DIVISION

I, **Do Cong Khanh**, holding the ID Card No.121476785, issued on March 09, 2007 by Public Security of Bac Giang Province, do hereby undertake this is true and exact translation from the Vietnamese version attached.

CHỨNG THỰC CỦA PHÒNG TƯ PHÁP QUẬN CẦU GIẤY, THÀNH PHỐ HÀ NỘI

Chứng thực ông **Đỗ Công Khanh,** CMTND số 121476785, cấp ngày 09/03/2007 tại Công An tỉnh Bắc Giang, đã ký trước mặt tôi tại Phòng Tư pháp quận Cầu Giấy, Tp Hà Nội.
Số chứng thực: 117 Quyển số: 04 SCT/CK
Ngày 13 tháng 04 năm 2010

TRƯỞNG PHÒNG TƯ PHÁP

PHÒNG TƯ PHÁP

Nguyễn Thị Đức Hạnh

Tôi, **Đỗ Công Khanh,** CMTND số121476785 cấp ngày 09/03/2007 tại Công An tỉnh Bắc Giang, cam đoan đã dịch chính xác, phù hợp với nội dung văn bản đính kèm từ tiếng Việt sang tiếng Anh.

Ngày 13 tháng 04 năm 2010
Người dịch

Đỗ Công Khanh

LIÊN HIỆP CÁC HỘI KH & KT VIỆT NAM
TRUNG TÂM SÁNG KIẾN SỨC KHỎE VÀ DÂN SỐ

HỘI ĐỒNG PHẢN BIỆN TRONG NCYSH
Số: 0904 /HĐPB-CCIHP
V/v chấp thuận các vấn đề đạo đức NCYSH

CỘNG HÒA XÃ HỘI CHỦ NGHĨA VIỆT NAM
Độc lập – Tự do – Hạnh phúc

Hà Nội, ngày 09 tháng 4 năm 2010

CHẤP THUẬN CỦA HỘI ĐỒNG PHẢN BIỆN TRONG NGHIÊN CỨU Y SINH HỌC

TRUNG TÂM SÁNG KIẾN SỨC KHỎE VÀ DÂN SỐ

- Căn cứ Quyết định số 1504 QĐ/CCIHP-2009 ngày 15 tháng 4 năm 2009 của Giám đốc Trung tâm Sáng kiến Sức khỏe và Dân số về việc thành lập Hội đồng Phản biện trong nghiên cứu y sinh học (Gọi tắt là Hội đồng Phản biện – HĐPB) xét duyệt các vấn đề đạo đức trong nghiên cứu y sinh học của các đề tài/dự án.

- Căn cứ vào Qui chế Tổ chức và Hoạt động của Hội đồng Phản biện của Trung tâm Sáng kiến Sức khỏe và Dân số;

- Trên cơ sở biên bản họp của HĐPB ngày 2 tháng 4 năm 2010 (có biên bản kèm theo).

Nay, HĐPB của Trung tâm Sáng kiến Sức khỏe và Dân số chấp thuận (cho phép) về các khía cạnh đạo đức trong nghiên cứu đối với đề tài / dự án:

- Tên đề tài: Đánh giá hiệu quả các dịch vụ can thiệp của Dự án Alive & Thrive đối với thực hành nuôi dưỡng trẻ em và trẻ nhỏ (IYCF) và phòng chống thấp còi ở trẻ em nông thôn Việt Nam
- Chủ nhiệm đề tài: Nguyễn Đức Minh
- Đơn vị chủ trì: Viện nghiên cứu Y-Xã hội học (ISMS)
- Địa điểm triển khai nghiên cứu: Bốn tỉnh: Đăk Lăk, Quảng Ngãi, Thanh Hoá và Vĩnh Long.
- Thời gian nghiên cứu: từ ngày 15/3/2010 đến ngày 15/8/2010

Ngày chấp thuận (cho phép): 09/4/2010

CHỦ TỊCH HỘI ĐỒNG

Vũ Song Hà

THƯ KÝ HỘI ĐỒNG

Nguyễn Thị Vịnh

IRB-CCIHP IORG number 0005789, FWA number 00014297
