# Supplementary material for: Impacts on Breastfeeding Practices of At-Scale Strategies That Combine Intensive Interpersonal Counseling, Mass Media, and Community Mobilization: Results of Cluster-Randomized Program Evaluations in Bangladesh and Viet Nam
Source: PLoS Med. 2016 Oct 25;13(10):e1002159. doi: 10.1371/journal.pmed.1002159 (PMC5079648; doi:10.1371/journal.pmed.1002159)
Supplement: S3 IRB — (PDF) [file pmed.1002159.s012.pdf]

INTERNATIONAL FOOD POLICY RESEARCH INSTITUTE
IFPRI

IRB #00007490
FWA #00005121

Project title: Evaluating the infant and young child feeding initiative, Alive and Thrive (A&T) in Bangladesh, Ethiopia, and Vietnam
Division: PHND
PI: Marie Ruel, Purnima Menon
Country of study: Bangladesh, Ethiopia, Vietnam
Date of initial IRB approval: April 28, 2010

Dear Marie and Purnima,

Our records confirm that the project "**Evaluating the infant and young child feeding initiative, Alive and Thrive (A&T) in Bangladesh, Ethiopia, and Vietnam**" was approved by the IFPRI IRB on April 28, 2010. This project was reviewed and approved at a time when the IFPRI IRB issued approvals digitally within the full-length application document rather than the separate approval letter that is our current practice; this letter serves as a record of approval in lieu of the full, signed digital application document. All approved application materials, consent and survey documents, and local ethical approvals are on file with the IFPRI IRB. We are happy to provide additional documentation upon request.

Sincerely,
Eduardo Maruyama
IRB Chair

IFPRI-IRB@cgiar.org
