# Supplementary material for: Impacts on Breastfeeding Practices of At-Scale Strategies That Combine Intensive Interpersonal Counseling, Mass Media, and Community Mobilization: Results of Cluster-Randomized Program Evaluations in Bangladesh and Viet Nam
Source: PLoS Med. 2016 Oct 25;13(10):e1002159. doi: 10.1371/journal.pmed.1002159 (PMC5079648; doi:10.1371/journal.pmed.1002159)
Supplement: S1 Table — (DOCX) [file pmed.1002159.s013.docx]

**S1 Table: Key components of the A&T impact evaluation surveys**

| **Type of data** | **Questionnaire modules/questions** | **How used** |
| --- | --- | --- |
| Impact indicators | Child anthropometry  IYCF practices, including variables to measure World Health Organization-recommended IYCF indicators | Change between baseline and endline used to assess trends in impact indicators. |
| Process data – client outcomes | Maternal IYCF knowledge, beliefs, intentions, and self- efficacy  Awareness, trial, and adoption of key recommended practices | Used to assess if IYCF knowledge shifted over the project period and whether awareness of key recommended behaviors improved. |
| Process data – client access, exposure, and utilization of services | Antenatal and postnatal care.  Exposure and use of A&T services  Exposure to media messages | Used to assess if exposure to program channels was as expected. Allowed assessment of impact trends by levels of exposure to interventions. |
| Influencing/underlying factors – child level | Appetite  Child illness and health | Used to assess if child-level factors modify influence of exposure to interventions. |
| Influencing/underlying factors – maternal level | Education  Time constraints/time stress  Physical and mental well-being  Status of women and maternal decision making power in the household | Used to control for underlying maternal factors and analyze the influence of mothers’ constraints on their ability to benefit from A&T interventions. |
| Influencing/underlying factors – household level | Household socioeconomic status (constructed from reported assets)  Household economic shocks  Household food security | Used to assess if the underlying factors shifted over the project period. In addition, it is used for controlling the impact of SES, economic shocks, and food security on IYCF impact indicators. |
