# Supplementary material for: Impacts on Breastfeeding Practices of At-Scale Strategies That Combine Intensive Interpersonal Counseling, Mass Media, and Community Mobilization: Results of Cluster-Randomized Program Evaluations in Bangladesh and Viet Nam
Source: PLoS Med. 2016 Oct 25;13(10):e1002159. doi: 10.1371/journal.pmed.1002159 (PMC5079648; doi:10.1371/journal.pmed.1002159)
Supplement: S1 Text — (DOCX) [file pmed.1002159.s014.docx]

**S1 Text: Examining Social Desirability Bias**

Social desirability is the tendency of respondents to answer questions in a manner that is viewed favorably by others. Social desirability can interfere with the interpretation of average tendencies as well as individual differences. Recognizing the potential role of social desirability in influencing reporting of nutrition behaviors such as breastfeeding (BF) practices, especially among beneficiaries of recent intensive counselling and mass media on BF practices, we examined potential bias from social desirability in our A&T impact analysis. A brief set of items was designed to assess the extent to which respondents were likely to report behaviors based on their desire for social approval, i.e., “social desirability”.

Previous efforts to measure and quantify social desirability were reviewed, and a subset of items was adapted from Reynolds and Gerbasi [[1](#_ENREF_1)]. We administered this subset of five questions to assess social desirability bias based on a review of the most commonly used measures, including the Marlowe-Crowne scale.  Based on internal review of scale items and local pretesting, a set of 5 items were included in the questionnaires and administered together with the primary measures of the study at endline.  The social desirability score (SDS) was created by adding up the number of socially desirable answers, out of the five questions in the module. Therefore, the SDS ranges from 0-5, with a SDS of 0-2 being considered as a low score, 3 as medium score, 4 as high, and 5 as very high. The SDS had a Cronbach’s alpha of 0.66, signifying that the items had moderate internal consistency. The key assumption is that respondents who answer in a socially desirable manner on the scale may also respond to other questions (e.g., those related to IYCF practices) in a manner they think is socially desirable.

We conducted three types of analyses using this scale. First, we calculated the mean SDS by program group to check for differences in social desirability between the two A&T study groups. Second, we tabulated the core BF practices by SDS to assess whether reported practice varied by score on the SDS. Third, we ran a set of regressions with each of the core BF practices as dependent variables to test the interaction between the SDS and the A&T program variable (i.e., intensive vs. non-intensive); this allowed us to assess whether or not social desirability differentially affected reported BF practices among the A&T-intensive group, and hence the overall impact estimates comparing changes over time in intensive and non-intensive groups. The third analysis was critically important because presence of social desirability does not necessary result in bias. If, for example, both comparison groups are affected equally, social desirability results in an overall over-estimate of prevalence in both groups, but does not bias the comparison between the two groups. Bias results when the effect of social desirability on a given outcome is differential between the two comparison groups.

In Bangladesh, the mean SDS was 2.91 ±1.14 for intensive vs 2.82 ±1.16 for non-intensive areas (p=0.015 for difference between the two groups). There was no evidence of an interaction between SDS and the A&T program variable for EBF (**S2 Figure**) and EIBF (**S3 Figure**) and therefore no bias due to social desirability.

In Vietnam, the mean SDS was higher, 3.65 ±1.05 for intensive vs 3.55 ±1.07 for non-intensive areas (p=0.002 for difference between the two groups). There was a tendency for women with high SDS to have higher EBF (**S2 Figure**) and IEBF (**S3 Figure**). There was no evidence of an interaction between SDS and the A&T program variable for EIBF, but there was evidence of an interaction for EBF. As SDS increased, EBF increased differentially in the intensive and non-intensive groups, with a greater increase in the intensive group. The difference in EBF prevalence at endline between the intensive and non-intensive groups when SDS was low was estimated as 16.65 (p=0.024). That is, the intensive group had significantly higher EBF prevalence at endline than the non-intensive group among women with low SDS. This difference of 16.65 was used to adjust EBF prevalence at endline for the intensive group overall and then to adjust the difference-in-difference estimate (DDE), simulating what would have been observed if social desirability had been non-differential. The DDE after adjustment for social desirability bias was 15.2 pp (p=0.008); the DDE not adjusted for social desirability bias was 27.9 pp (p<0.0001).

1. Reynolds WM. Development of reliable and valid short forms of the Marlowe-Crowne Social Desirability Scale. Journal of Clinical Psychology. 1982;38:119-25.
