# Supplementary material for: Impacts on Breastfeeding Practices of At-Scale Strategies That Combine Intensive Interpersonal Counseling, Mass Media, and Community Mobilization: Results of Cluster-Randomized Program Evaluations in Bangladesh and Viet Nam
Source: PLoS Med. 2016 Oct 25;13(10):e1002159. doi: 10.1371/journal.pmed.1002159 (PMC5079648; doi:10.1371/journal.pmed.1002159)
Supplement: S2 Text — (DOCX) [file pmed.1002159.s015.docx]

| 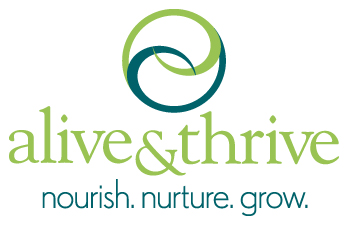 |
| --- |
| **S2 Text**  **Survey Protocol for Impact Evaluation & Statistical Analysis** |
| **BANGLADESH** |
|  |
| Purnima Menon  Kuntal Saha  Adiba Khaled  Rahul Rawat |
|  |

**Table of Contents**

[INTRODUCTION 1](#_Toc456972725)

[OBJECTIVES 1](#_Toc456972726)

[THE INTERVENTION 2](#_Toc456972727)

[Description of the Alive & Thrive Program in Bangladesh 2](#_Toc456972728)

[Conceptual framework 4](#_Toc456972729)

[EVALUATION DESIGN 4](#_Toc456972730)

[Sample size and power calculations 6](#_Toc456972731)

[Data collection methods 7](#_Toc456972732)

[STATISTICAL ANALYSIS PLAN 10](#_Toc456972733)

[Overall approach 10](#_Toc456972734)

[Impact indicators 11](#_Toc456972735)

[Detailed statistical analysis approach 11](#_Toc456972736)

[PUBLICATION PLANS 13](#_Toc456972737)

[ANNEXES 14](#_Toc456972738)

[Annex 1. Description of modules in household questionnaire 14](#_Toc456972739)

[Annex 2. Randomization of upazila for Alive & Thrive baseline study in BRAC EHP areas 16](#_Toc456972740)

# INTRODUCTION

Despite significant improvement in the nutrition scenario over the past decades, the prevalence of child undernutrition in Bangladesh remains alarmingly high and infant and young child feeding practices, especially complementary feeding, are poor [[1](#_ENREF_1)]. The evidence available at the beginning of the A&T initiative in 2009 indicated that inadequate IYCF practices were the most important proximal determinants that contribute to the high burden of child undernutrition in Bangladesh and that nutrition education and behavior change communication were associated with improved IYCF practices*.* A recent study in Bangladesh had suggested that appropriate IYCF practices recommended by the WHO and UNICEF resulted in better growth of rural Bangladeshi infants and young children of 0-24 months of age [[2](#_ENREF_2)]. In Bangladesh, intensive nutrition education significantly improved the status of moderately undernourished children with or without supplementary feeding [[3](#_ENREF_3)]. An earlier study in Bangladesh also suggested that behavior change communication through trained counselors to promote and support breastfeeding was very successful – 70 percent of the mothers in the program areas breastfed their infants exclusively for 5 months of age compared to only 6 percent in the comparison areas [[4](#_ENREF_4)]. Although these were well-designed studies, these were efficacy studies. Evidence on the impact of large-scale programs to improve IYCF and nutrition in Bangladesh is less promising [[5](#_ENREF_5),[6](#_ENREF_6)].

Scaling up interventions to improve key direct determinants of undernutrition in Bangladesh, such as IYCF practices, are essential to accelerate progress toward reduction in undernutrition. Alive & Thrive’s portfolio of activities in this context has significant potential for impact. Rigorous impact evaluations are also essential to make contributions to the policy dialogue in Bangladesh and A&T’s impact evaluation fills a significant knowledge gap by examining impact at scale, using a rigorous evaluation design.

Alive & Thrive (A&T) is an initiative funded by the Bill & Melinda Gates Foundation to reduce undernutrition and death caused by suboptimal IYCF practices in three countries (Viet Nam, Bangladesh, and Ethiopia) over a period of six years (2009-2014). Alive & Thrive is led by FHI360 and is implemented through a partnership with Save the Children, GMMB, the International Food Policy Research Institute (IFPRI), and the University of California, Davis. In Bangladesh, this initiative is implemented in close partnership with BRAC and several other partners. A&T’s Bangladesh strategy is designed to support improvements in Infant and Young Child Feeding (IYCF) in three key ways: 1) improving policy and regulatory environments; 2) shaping IYCF demand and practice; and 3) increasing supply, demand, and use of high quality complementary foods. In order to achieve this, the A&T Bangladesh program has been divided into three main focus areas namely advocacy, community, and the private sector. In addition, a communications component is integrated into each of these focus areas to support their activities. The community-based model implemented by BRAC is a core initiative of the community model to provide quality nutrition counseling to women and families through home visits, to mobilize diverse members of the community and to bring media spots to media-dark areas.

# OBJECTIVES

The objectives of this impact evaluation are primarily to assess the impact of the package of A&T interventions delivered through the BRAC community-based platform that combines frontline health worker interventions and social mobilization. Using a pre-post cluster-randomized design with a comparison group (see below), our objectives are

1. To assess the impact of the BRAC-platform delivered interventions on IYCF practices among children 0-23.9 months of age;
2. To assess the impact of the BRAC-platform delivered interventions on stunting among children 24-47.9 months.

Having comparison areas that also receive other A&T interventions, such as the media campaigns, also allows us to capture shifts in IYCF practices, knowledge, norms, and perceptions over time that is due to those components alone. We will therefore also explore the use of data from the comparison areas, to generate evidence on the role of the media activities to shape demand and practice.

The impact evaluation is set up to address the following specific hypotheses:

1. Age-appropriate IYCF practices (EBF for 0-5.9 months or breastfeeding + age-appropriate complementary feeding for 6-23.9 months) will be better among children living in areas covered by the A&T intensive interventions (counseling, social mobilization and mass media) than those living in areas without the intensive interventions (only mass media).
2. Children 24-47.9 months of age in areas covered by the A&T intensive interventions will have lower levels of stunting than children 24-47.9 months of in areas with just the mass media intervention.

In addition to these objectives related to the community-based interventions, our design also allows us to assess the effects of the mass media campaign through the use of pre- and post-data collection at baseline and endline and the use of data on exposure to the mass media campaigns. We will use the extensive survey data to control for potential confounding in such analyses.

# THE INTERVENTION

## Description of the Alive & Thrive Program in Bangladesh

A&T’s mandate is to facilitate change for improved IYCF practices at scale; to document how interventions are delivered, how much impact can be achieved and at what cost, and to disseminate the evidence and lessons learned so that others can adapt and replicate the cost-effective components.

The goal of the A&T initiative is to reduce avoidable death and disability due to suboptimal IYCF. The specific objectives include an increase in exclusive breastfeeding (EBF) until 6 months of age and reduce stunting by delivering IYCF interventions to caregivers and families of children 0-24 months of age. A&T’s overarching model is based on the assumptions that sustainable improvements in breastfeeding and complementary feeding is achievable through strategies that improve the policy and regulatory environment to support IYCF interventions and practices.

The major goals of the A&T initiative in Bangladesh were to achieve the following in project areas:

1. Increase exclusive breastfeeding (EBF) by 49 percent (from 43 percent to 65 percent) among infants 0-5.9 months of age.
2. Reduce stunting by 10 percent (from 43 percent to 39 percent) among children of age 24-47.9 months.
3. Reduce anemia by 10 percent (from 50 to 45 percent) among children 6-23.9 months of age.

In Bangladesh, A&T’s comprehensive delivery model in Bangladesh largely consisted of a community component implemented through BRAC’s Essential Health Program (EHC) supported by a national advocacy component and a mass media communication strategy [[7](#_ENREF_7)]. A&T also worked in partnership with other local and international NGOs to intensify and scale up activities on infant and young child feeding (IYCF) at the national level as well as to guide the Government of Bangladesh (GOB) in mainstreaming nutrition into the five-year health sector plan. The A&T model also included efforts to actively engage private sector entities to broaden sources of investment for IYCF-related services. Advocacy component of the A&T focused on journalists, the press/media, medical and education sector leaders, and donors funding the next health sector strategy.

BRAC’s EHC program operates in several rural sub-districts *(upazilas)* of Bangladesh. The management structure of the A&T interventions, as delivered through through EHC is illustrated in Figure 1**.** Through the EHC platform, BRAC provides affordable and accessible health services to the poor through frontline workers (FLW): *Shasthya Shebika* (SS, community health volunteers) and their supervisors, the *Shasthya Kormi* (SK, community health workers). In EHC sub-districts, several cadres of workers help shape IYCF demand and practice. The SS and the SK were the front line service providers for A&T in 50 sub-districts with EHC programs who delivered age-appropriate IYCF counseling and support services during home visits, antenatal and postnatal sessions, and health forums. IYCF Promoters (PK) recruited for A&T interventions supported the IYCF work of the SS and SK. All of these FLWs were women. Mobile phones and monthly meetings were used to network these teams of workers within sub-districts and unions. All SS, SK and PK were expected to perform specific tasks during the household visits (Table 1) and were expected to give key messages for infants and young children of different age groups (Table 2).

Typically, SS visit about 250-300 households a month that have children aged 0-23.9 months to provide advice and services, attend monthly refresher training for a day once every month, and sell medicines. Each SK supervises 10-15 SS, prepares monthly registers for the SS, conducts field visits 6 days a week, attends monthly one-day refresher training and does performance review along with the SS, and comes to office one day each month to complete monthly report. The SK is also expected to visit all the households in her catchment area every 6 months. SK in sub-districts involved with BRAC’s EHC program receive an additional monthly honorarium for spending two extra hours per day on IYCF-related activities.

The IYCF promoters (PK) were expected to prepare household lists including children 0-23.9 months of age and pregnant women. Each PK was expected to start working from the residence of SS according to daily work schedule. The catchment area for PK was defined such that: (i) there were not more than 350 children 0-23.9 months of age; (ii) there were not more than 2500 households; and (iii) she could visit children 0-23.9 months of age once in two months.

To build an enabling environment for improved IYCF practices at the community, A&T program organizers addressed community constraints through awareness-building activities such as meetings and forums with husbands, religious leaders, MNCH committee members, pharmacists and shopkeepers, doctors, local council chairmen and members. A&T recruited 25 IYCF monitors to follow the performance of the SS, SK, and IYCF promoters. The monitors conducted a check on sampled households each month; the results determine incentives for each SS. Monitors were also expected to identify areas requiring strengthening. One A&T program organizer for each sub-district was recruited to supervise and monitor all IYCF activities, including home visits and counseling and also generation of community support. The existing upazila, district and regional managers were responsible for coordination among different BRAC programs and for liaison with other NGOs, private providers, and local government bodies and health staff.

Figure 1. Operational structure for Alive & Thrive in the BRAC Essential Health Care platform

Table 1. Responsibilities of SS, SK and IYCF Promoters during household visits in Alive & Thrive Program areas^[[1]](#footnote-1)^

| SS | SK | IYCF Promoter (PK) |
| --- | --- | --- |
| - Enters in her Register all identified pregnant women and children 0-24 months of age in her catchment area. - Immediately informs IYCF Promoters about any new births. - Advices mothers and the families to put the baby to the mother’s breast in one hour of birth. - Confirms giving the baby colostrum. - Encourages mothers and the families exclusive breast feeding (EBF) to the babies 0-6 months of age. - Identifies mothers having problems with EBF and solves, if possible; immediately informs IYCF Promoters about this and follows up if necessary. - Refers mothers having problems with feeding her child to appropriate places if needed for the problems. - Encourages mothers and the families regarding feeding children 7-24 months of age the family foods in addition to breast milk. - Identifies mothers having problems with feeding children 7-24 months of age the family foods in addition to breast milk, solves if possible; immediately informs IYCF Promoters about this and follows up if necessary. | - Conducts health forum. - Provides healthcare during household visits and ensures the following activities: - Follows up SS. - Encourages family planning methods for eligible couples. - Observes DOTS of suspected TB patients. - Pregnancy identification. - Encourages and provides ANC and PNC services to the pregnant women. - Encourages vaccination to the target children and pregnant women. - Registers new births. - Encourages installation and proper use of sanitary latrines. - Ensures use of safe drinking water. - Gives messages regarding importance of hand washing with soap for children and all family members. - Responsibilities during household visits - Registers 0-24 month children identified by SS. - Advices mothers and the families to put the baby to the mother’s breast in one hour of birth. - Advices mothers and the families to feed colostrums to the newborns. - Encourages mothers and the families EBF to the babies 0-6 months of age. - Identifies mothers having problems with EBF and solves, if possible; immediately informs IYCF Promoters about this and follows up if necessary. - Refers mothers having problems with feeding her child to appropriate places if needed for the problems. - Encourages mothers and the families regarding feeding children 7-24 months of age the family foods in addition to breast milk. - Identifies mothers having problems with feeding children 7-24 months of age the family foods in addition to breast milk, solves if possible; immediately informs IYCF Promoters about this and follows up if necessary. | - The household visit schedule of the IYCF Promoter – - 1st visit: within 48 hours of birth. - 2nd visit: within 7-10 days after birth. - 3rd visit: 2 month 1 day – 2 months 29 days. - 4th visit: 5 months 1 day – 5 months 29 days. - 5th visit: 6 months 1 day – 6 months 15 days. - 6th visit: 8 months 16 days – 9 months 15 days. - 7th visit: 12 months 1 day – 16 months 29 days. - 8th visit: 18 months 1 day – 23 months 29 days. - Visits households according to the schedule and - - Enlists children 0-24 months of age. - Encourages mothers and the families exclusive breast feeding (EBF) to the babies 0-6 months of age. - Encourages mothers and the families regarding feeding children 7-24 months of age the family foods in addition to breast milk. - Demonstrate appropriate techniques of feeding breast milk and other family foods to the mothers. - Problem solving related to breast feeding, feeding other family foods and follow up – - Holds forums with mothers regarding health and nutrition education. - If needed, works out of working hours for emergency situation. - Attends training, refresher training and meetings as needed. - Completes daily report forms, prepares monthly report of her catchment area and submits monthly reports to POs. |

Table 2. Key tasks for different age groups given by SS, SK and IYCF Promoters (PK) at household visits in Alive & Thrive program areas

| **0-5.9 months of age (0-180 days)** | **6 – 23.9 months of age** |
| --- | --- |
| - Demonstrate correct position and attachment of breastfeeding. - Follows up, if necessary, whether the infants are exclusively breastfed up to 6 months of age. - At 5.5 months of age, discuss with mothers and the families the importance of feeding family foods in addition to breast milk. - Prepares mothers and the family mentally to introduce family foods in addition to breast milk when the infants complete 6 months of age. | ***When the babies complete 6 months of age:***   - Discuss with mothers and the families the importance, quantity and preparation of family foods in addition to breast milk according to the age of the children. - Encourage mothers and the families to feed their children animal source foods (fish/meat/egg), milk products, fried/oily foods, colored vegetables, fruits and adding oil in foods of children. - Discuss with mothers and the families the quantity and density of daily foods of children.   ***When the babies complete 9 months****:*   - Discuss with mothers and the families that they should encourage their babies to eat by themselves. - Discuss feeding a sick child (frequent feeding, feeding child the foods that she/he likes). - Discuss importance of continuing breastfeeding up to 24 months of age in addition to family foods. |

##

## Conceptual framework

The conceptual framework for the Alive & Thrive strategy in Bangladesh is shown in Figure 2. The diagram illustrates all major components of A&T’s strategic mix of interventions in Bangladesh. This framework highlights that behavior change through interpersonal communication between mothers/caregivers and properly trained FLW can create awareness and skills among mothers/caregivers to take proper care of their children. Family support is also considered to be an essential element for ensuring proper IYCF practices. Therefore, other family members, particularly fathers and grandmothers, have been targeted through A&T’s social mobilization and media interventions, to help build support for mothers/caregivers in their homes. Along with behavior change communications, social mobilization, therefore, plays a significant role in building awareness among other household members, such as fathers, in-laws, and adolescents, as well as important people in the community, such as school teachers, religious leaders, and government health professionals. These community members are thought to play an important role in creating a favorable environment for the mothers/caregivers for proper IYCF practices.

Communications via media is another major component of A&T Bangladesh. Behavior change communications using media is intended to help support the interpersonal communication interventions to shape overall behavior change. Finally, the role of advocacy to formulate and/or implement favorable policy to create enabling policy environment for IYCF is crucial and is well-recognized in A&T’s strategy in Bangladesh. A&T’s mandate, in sum, is to improve IYCF practices using multiple platforms through improving mothers/caregiver’s knowledge and skills regarding IYCF practices.

Figure 2. Conceptual framework of Alive & Thrive program in Bangladesh


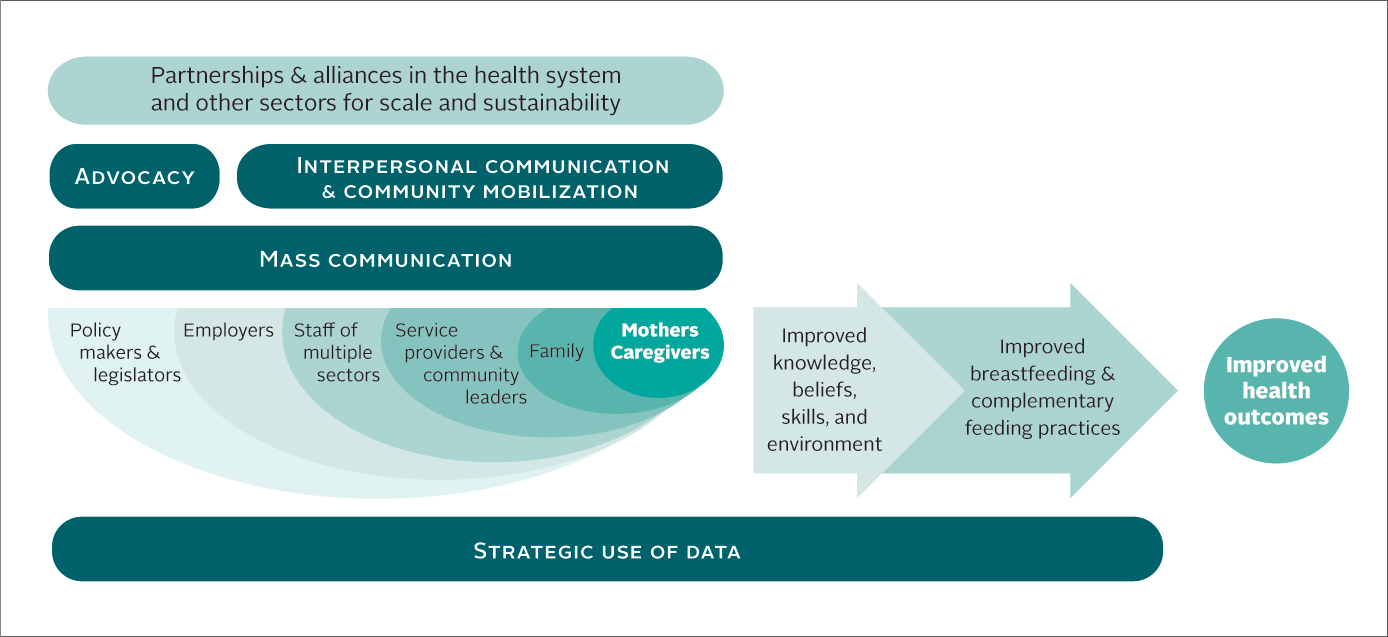


# EVALUATION DESIGN

The impact evaluation of community component A&T interventions delivered through the EHC program uses a cluster-randomized design with repeated cross-sectional surveys at the baseline and endline (Figure 3). For the impact evaluation of A&T’s community-based rural platform, 20 rural subdistricts (*upazilas*) that were part of BRAC’s Essential Health Care (EHC) program platform for A&T were purposively chosen. The objective of this impact evaluation model is to capture the synergistic impact of A&T’s community component along with media communications and private-sector activities, such as the promotion and integration of micronutrient powders.

The impact evaluation is designed to capture impact on all key A&T indicators (stunting, infant and young child feeding (IYCF), and anemia). Using a repeated cross-sectional survey design, impact on stunting will be assessed in children 24-48 months old. The impact on other IYCF practices and anemia will be assessed in children 6-23.9 months old; and the impact on exclusive breastfeeding (EBF) will be assessed in children 0-5.9 months old. A double difference impact analysis as well as analysis by exposure level to interventions will be employed to determine the impact of A&T interventions.

The baseline survey was in April to July 2010, and an endline survey is planned for April to July 2014.

The main outcomes being examined are changes in mean height-for-age Z-score (HAZ) and changes in the prevalence of childhood stunting between baseline and the post-intervention survey. Other core impact indicators for A&T in Bangladesh include changes in age-appropriate WHO-recommended IYCF practices among children under 2 years of age (EBF for children 0-5.9 months old and breastfeeding + age-appropriate complementary feeding for children 6-23.9 months old) and anemia among children 6-23.9 months of age.

In Bangladesh, 100 sub-districts, across five of six divisions, were selected by BRAC as possible A&T intensive areas based on high poverty and stunting levels, and sub-districts that were not included in the government National Nutrition Program. This list was narrowed to 78 based on geographic proximity, size, and other operational aspects to ensure homogeneity across the sample. Within each division, four sub-districts were then randomly selected for inclusion in the evaluation sample using a computer program, for a total of 20 sub-districts. Sub-districts within each division were then randomly assigned, using a computer program, to either the *intensive* (10 sub-districts) or *non-intensive* (10 sub-districts) intervention. The randomization process was carried out in the presence of BRAC and A&T staff and the program evaluators in BRAC’s headquarters in Dhaka.

The baseline survey was conducted in 20 rural *upazilas* that are part of BRAC’s Essential Health Care (EHC) program platform for A&T. The baseline survey had three components—(i) household survey, (ii) community survey, and (iii) staff survey.

**Figure 3. Impact Evaluation Design**

**
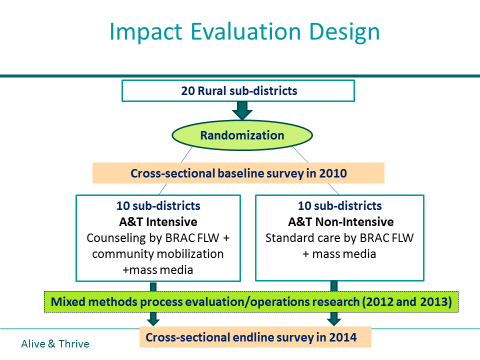
**

## Sample size and power calculations

Sample sizes were estimated based on detectable differences between A&T and non-A&T *upazilas* for three separate sets of indicators: 1) exclusive breastfeeding (EBF) rates, 2) complementary feeding practices, and 3) stunting. Our sample size estimates accounted for randomization at the cluster (i.e., *upazila*) level rather than at the individual level. Surveys are repeated cross-sectional, surveying different households/children from the same communities.

The estimated sample sizes for each treatment arm, calculated prior to the baseline survey, was 500 infants 0-5.9 months old for EBF, 500 children 6-23.9 months old for complementary feeding-related practices, and 1,100 children 24-47.9 months old for stunting. Thus, the total proposed sample size was 4,200 children 0-47.9 months old. This sample size was estimated to allow us to detect expected differences in the outcomes outlined below, in a total of 20 clusters with an inter-cluster correlation (ICC) of 0.005, a significance level of 0.05, and power of 80 percent.

A sample size of 500 children aged 0-5.9 months of age was deemed sufficient to detect an increase in EBF rates by approximately 49 percent (from 43 percent to 64 percent); a sample size of 500 children aged 6-23.9 months of age is sufficient to detect an increase in several complementary feeding practices by at least 25 percent; a sample size of 1,100 children aged 24-47.9 months of age is sufficient to detect a reduction in stunting by 15 percent (from 43 percent to 36.5 percent).

Detailed sample size calculations are presented in **Table 3**.

**Table 3: Sample size calculation for household survey**

|  | **Baseline sample size (same proposed for endline)** | **Baseline (%)** | **Detectable difference with sample size proposed for endline survey**** | **Intra-class correlation at baseline (estimated at upazila level)** | **Minimum sample size for detectable differences** |
| --- | --- | --- | --- | --- | --- |
| Stunting (24-47.9m) |  | 50.9 | 10 pp | 0.02 | 920 (detecting a 9 pp difference will require double this sample size) |
| HAZ (24-47.9m) |  | -2.0±1.2 | 0.35 | 0.05 | 519 |
| EBF (0-5.9m) |  | 49.9 | 20.0 | 0.12 | 390 |
| Minimum acceptable diet (6-23.9m) |  | 14.8 | 10 | 0.04 | 323 |
| Anemia among children  6-23.9 mo |  | 74.7 |  | 0.00 | 284 |

**Based on at least maintaining levels seen in the PE 2013 survey (where data available; data on stunting and anemia not available in PE 2013).*

***Detectable differences are estimated for endline-only comparisons between A&T-intensive and A&T-non intensive; double difference estimates will ensure greater statistical power.*

For the FLW survey at endline, we will again interview BRAC frontline workers delivering services in the 200 villages covered in the sampling for the evaluation. We estimate that the interviews would cover up to 300 SS, 100 SK, and 100 PK.

**Sampling**

The sampling at endline will be identical to the sampling procedures followed at baseline and the same communities will be surveyed at both points in time:

- From each *upazila*, five unions will be randomly selected.
- Then, two villages will be selected randomly from each of the selected unions, to yield a total of 200 villages from which survey households will be selected. Only households with children 0-48 months of age were selected at baseline, and therefore, the same age group will be measured in the endline too.
- The baseline survey included a household listing during which a census of households in the 200 villages selected for the survey was conducted. This will now be repeated for the endline as well, following the same procedures as at baseline, including demarcations of villages into enumeration areas, if required.

## Data collection methods

The survey comprises of five main components:

- Household listing and census
- Household survey
- Anthropometric measurements of children and mothers and hemoglobin assessments
- Frontline worker surveys
- Community questionnaires

***Census form***: The census form will capture information captured at baseline, and will include administrative information of the location of the household, information on the household composition, and literacy and main occupation of all household members. The questionnaire will include, as at baseline, some basic household-level information as well as information on exposure to BRAC’s health program. In addition, the census questionnaire asked if the household had a child 0-48 months of age, to develop the on-site sampling list.

***Household questionnaire***

The household questionnaire will be administered to the mother of the index child chosen for the survey. In addition to the questionnaire-based data collection, anthropometric measurements (height and weight) will be taken for mother and index child in each household. The endline household questionnaire is being developed by using the baseline evaluation questionnaires as a base, and adapting it to capture key program activities, particularly with regards to the use of A&T community services and exposure to mass media. Table 4 below presents an overview of how the data collected in the household questionnaire is used in analysis and interpretation of impact results. We will explore the possibility of capturing exposure by fathers and grandmothers to the interventions but will need to explore this in light of the survey budgets.

**Table 4. Impact, process, and underlying factors captured by the baseline and endline household questionnaire**

| **Type of data** | **Questionnaire modules/questions** | **How used** |
| --- | --- | --- |
| Impact indicators | Child anthropometry  WHO-recommended IYCF indicators | Change between baseline and endline used to assess trends in impact indicators |
| Process data – client outcomes | Maternal IYCF knowledge  Awareness, trial, and adoption of key recommended practices  Behavioral determinants | Helps assess if IYCF knowledge shifted over project period, whether awareness of key recommended behaviors improved |
| Process data – client access, exposure, and utilization of services | Exposure to FLW counseling services and mass media  Utilization and coverage  Prenatal and postnatal care | Helps assess if exposure to intended program channels is as expected. Will also allow assessment of impact trends by levels of exposure to interventions |
| Influencing/underlying factors – child level | Child illness and health  Child development/milestones  Hygiene and hand washing | Helps assess if child-level factors modify influence of exposure to interventions. Child development milestones also function as an outcome/impact indicator in relation to IYCF practices and micronutrient powders. |
| Influencing/underlying factors –maternal level | Education, working condition, time constrains and child care arrangement.  Physical and mental well-being  Women’s status and maternal decision making power at the household | Maternal well-being is a prerequisite for intervention success. We would use these data to control for underlying mother factors and analyze the influence of mother constraints on ability to benefit from the A&T interventions. |
| Influencing/underlying factors – household level | Household SES (construction of SES scale from assets)  Household economic shocks  Household food security | Economic shocks could seriously undermine project impact. Additionally, improvements in SES over time could attribute greater impact to the project than is true. Therefore, data on SES, shocks, and food security are important both to control for these influences in analyses of change over time, as well as to examine the influence of household-level constraints on the ability to benefit from A&T interventions . |

***Frontline health worker questionnaire***

Gathering data on FLWs at baseline and endline enables us to document changes in FLW performance over time and contributes to an understanding of the impact pathways through which A&T objectives are met. Since FLWs are a critical factor in the overall impact and process pathways, it is essential to capture issues related to their performance as part of the overall evaluation. The literature on FLWs and their work context is slim in the field of nutrition programs and nutrition program evaluations. Based on the literature, available validated scales, and our past experiences studying this issue in evaluations, we have included in the A&T evaluation questionnaires the following domains (Table 5) that relate to frontline health workers and their performance, particularly in relation to IYCF counseling. If deemed necessary, we could also interview village doctors (who were interviewed at baseline).

**Table 5. Overview of the domains covered in the A&T frontline health worker questionnaires**

| **Domains** | **Rationale for inclusion** | **How used in evaluation** |
| --- | --- | --- |
| Technical knowledge and skills related to IYCF | A basic prerequisite for delivering the franchise model successfully is knowledge of FLWs about the core topics to be covered in the franchise model. We included the same IYCF knowledge assessments for FLWs and mothers in our questionnaires. Empirical evidence indicates that the amount of correct knowledge that is shared by FHWs and beneficiaries influences the probability that beneficiaries will try new behaviors. | Document improvements/shifts in technical knowledge and skills due to A&T inputs |
| Training exposure | In order to effectively deliver the IYCF service delivery package, FLWs require training to develop their competency in performing required duties. In addition, training is known to be a motivating influence on FHWs. | Document exposure of FLWs to training and impact of training |
| Media exposure | Mass media advertisements have potential to influence FHWs’ knowledge, work and behaviors. | Evaluate impact of mass media |
| Work load and time commitments | Information on workload and time commitments are needed for three purposes: 1) to understand demands made by A&T on workload and time commitments; 2) to understand how perceptions of workload, in addition to actual workload, influence FLW motivation and performance; and 3) to use data on time commitments, and shifts in time commitments and activities due to the interventions in the costing studies. | Compare baseline and endline. Used in costing component to estimate changes in time allocations due to A&T |
| Motivation and job satisfaction | A number of factors influence FLW motivation. These include factors that are extrinsic to the FHW as well as intrinsic motivators. Motivation is strongly related to job performance; therefore, capturing perceptions related to motivation and motivators is important both at the baseline and endline as well as in the interim (process evaluation). Job satisfaction is also an influence on job performance, and therefore important to capture. | Establish baseline; document shifts in motivation/job satisfaction due to A&T inputs such as training, incentives, supportive supervision. |
| Self-efficacy and confidence | Self-efficacy and confidence is a basic driver of job motivation and job performance. | Establish baseline; document improvements/shifts due to A&T inputs and assess role of confidence/self-efficacy in performance of FLWs |
| Supervisory support | Supportive supervision is documented to be critical in FLW performance, motivation, and training. | Establish baseline; document improvements/shifts due to A&T inputs and assess/document role of supervisory support in performance of FLWs implementing A&T interventions |
| Basic demographics | Demographic characteristics may have influences on worker performance and effectiveness. | Control for basic demographics in analyses of factors. |

***Community questionnaire***

The purpose of the community questionnaire is to gather data on underlying factors at the community level, and to compare the different clusters over time. The community questionnaire also captures the causes of undernutrition that are embedded within the larger societal and environment factors. The community questionnaire was designed to provide information on the following aspects of each village in the sampling area:

- General characteristics of the village: population, percentage of minority and poor household, main source of income in peak and low seasons
- Infrastructure of the village: access to main road, electric, gas, and irrigation systems
- Distance from the nearest major town, type of transportation used to reach this town
- Natural disasters occurring in the village during the last year
- Presence of other organizations or support groups

**Ethical approval**

The evaluation has been approved by IFPRI’s Institutional Review Board, and the Bangladesh Medical Research Council in Bangladesh.

**Timeline for endline survey**

The survey timeline is depicted in the table below. As the baseline data collection was conducted starting on April 8, 2010, the endline data collection will also start that same week in 2014. The data collection schedule will be identical to that at baseline, to avoid seasonality effects for the same upazilas as best as possible. Data collection is scheduled to end by the end of June, 2014.

# STATISTICAL ANALYSIS PLAN

## Overall approach

The overall approach to the A&T impact analysis involves two major steps:

1. Accurately **estimate the impact** of the A&T-intensive interventions on IYCF practices and anthropometric outcomes
2. **Enhance plausibility** of impact estimates through:
   1. **Examining social desirability bias** for IYCF practices, especially for those practices (EBF) that are susceptible to systematic reporting bias
   2. Assessment of **biological plausibility** of changes in outcomes based on age of initial exposure and duration of exposure
   3. ***Impact pathway analysis*** that documents exposure to A&T interventions
   4. Analysis of ***trends in underlying determinants*** of IYCF practices and child growth, to rule out alternative reasons for trends over time

The overall analysis will examine and adjust, as needed, both for time invariant characteristics that can have impact on outcomes (i.e., geographic clustering) and time variant determinants where important changes were seen over time (e.g., women’s work patterns, food security, etc.).

The impact on exclusive breastfeeding (EBF) will be assessed in children 0-5.9 months old. The impact on other IYCF practices and anemia will be assessed in children 6-23.9 months old and the impact on stunting will be assessed in children 24-47.9 months old.

## Impact indicators

A&T’s core impact indicators are the 8 WHO-recommended IYCF indicators and child stunting. We will, however, examine impacts on all anthropometric indices – height-for-age, weight-for-age and weight-for-height. The indicators are depicted in Table 6 below:

**Table 6. Impact indicators**

| **Indicator** | **Age group (mo)** | **Remarks** |
| --- | --- | --- |
| ***Breastfeeding practices*** |  |  |
| Early initiation of breastfeeding | 0-23.9 |  |
| Exclusive breastfeeding under 6 months | 0-5.9 | We will also examine questions included in the endline survey that address social desirability bias |
| Continued breastfeeding at 1 year | 12-15.9 |  |
| ***Complementary feeding*** |  |  |
| Introduction of solid, semi-solid food, or soft food | 6-8.9 |  |
| Minimum diet diversity | 6-23.9 |  |
| Minimum meal frequency | 6-23.9 |  |
| Minimum acceptable diet | 6-23.9 |  |
| Consumption of iron-rich food | 6-23.9 |  |
| ***Anthropometry*** |  |  |
| HAZ | 24-47.9.9 | We will also examine impacts on HAZ among potentially fully exposed children (24-35.9 mo.) |
| Stunting | 24-47.9.9 | We will also examine impacts on HAZ among potentially fully exposed children (24-35.9 mo.) |

## Detailed statistical analysis approach

The statistical analyses will include intent-to-treat analyses, unadjusted and adjusted, for primary impact indicators. Several other analyses related to establishing plausibility of impacts will also be undertaken.

1. **Impact estimates**

Impact estimates in Bangladesh will be double difference estimates of changes in key impact indicators. This takes into account changes over time between baseline and endline in the intervention and comparison groups, and differences between groups at these times. The impact estimates will yield (1) **percentage point and percentage changes** for IYCF practices and stunting, wasting, and underweight, and anemia (Bangladesh only); and (2) change in **mean Z scores** for height for age, weight for age, weight for height, and hemoglobin (Bangladesh only).

Analyses will be done separately for each critical age group, e.g., exclusive breastfeeding will be assessed only in children 0-5.9 months old, complementary feeding in children 6-23 months old and anthropometry impacts in children 24 months and above. These reflect the different sample age groups for the impact evaluation. All analyses are in Stata.

Analyses will focus on the following:

- ***Pure* *Intent-to-treat*** analyses based on the original evaluation design and original age groups, using child-level data and fixed-effects analysis to achieve double difference estimates. The fixed-effects analysis will account for village, the smallest level repeated between baseline and endline. Further adjustment for geographic clustering at provincial/divisional, district and commune/subdistrict level will be done using random- or fixed-effects to adjust for clustering if needed; the need for this is not likely because of the accounting of variation among villages.
- ***Pure intent to treat in high potential for exposure age groups* –** this analysis will focus on the stunting indicator and capture impact estimates in an age group of children who are mostly likely, because of their age, to have been exposed to A&T’s interventions early in infancy and for the longest duration. This is the 24-35 month age group in both Bangladesh and Vietnam. We will examine the pattern of HAZ across the full 0-59 month age range, and look at impacts on stunting beginning at the age when HAZ begins leveling off i.e. possibly beginning at 18 months of age. We will use fixed-effects regression models to adjust for village clustering as above.
- ***Adjusted intent-to-treat* analysis** using data from the original ITT age groups but now adjusting for child age, child sex and other variables that might be different between the A&T-intensive and A&T-non-intensive age groups. We will use random- or fixed-effects regression models to adjust for geographic clustering.

1. **Plausibility analysis 1 – Examining social desirability bias**

Recognizing the potential role of social desirability bias in influencing reporting of nutrition behaviors, we plan to examine this within the A&T impact analysis in the following ways:

- Examine results of a diverse set of “trick” questions in the surveys that try and capture ways of triangulating findings on EBF and diet diversity (given both of these rely on the 24-hr recall of foods fed to the kids and the mothers might either under or over report in the recall instrument, depending on the child’s age group).  Estimates of IYCF indicators separately for groups that appear to be “fixing” their 24 hour recall responses. Examples include:
  - Asking elsewhere in the questionnaire (not the IYCF module) about availability of formula, feeding bottles, special bowl for the child, etc. “Child-specific” assets have been added into the household asset module, for instance, and we ask about books, toys and special feeding materials for the child.
  - In Bangladesh, include formula, tinned milk powder, and baby bottles in the consumption expenditure module
  - Ask to see all the things they use when they are feeding the babies, maybe – all bowls, plates, cups, bottles – and note this in a short observation
- Triangulate the 24-hr recall data from interviews with mothers by also examining data from the grandmother and father what foods the child ate in the last 24 hours. Triangulate in the analysis the concordance here.
- Examine data on stool frequency and consistency and urine frequency in the illness module (likely not very reliable because bigger threat to EBF is water in most places)
- Examine data from questions on social desirability tendencies

*Note: there is currently no way to correct for any situations where social desirability bias appears to be strong from the above assessment approach but we will report briefly the findings from these analyses as part of any impact papers.*

1. **Plausibility analysis 2 - based on biological plausibility and implementation plausibility (exposure to A&T interventions)**
   1. Examine **anthropometry outcomes by child age (**biological plausibility**) and duration of exposure** (intent to treat analysis for implementation plausibility).

- **Exposure variables** will include the following
  - FLW contact (use the question regarding # of contacts within the last 6 months))
  - Mass media exposure (use both the question of recall of ever having seen specific ads, with recall of at least x number of messages in the ad, or select messages )
  - Franchise contact (VN)
  - **MNP purchase and consumption variables (Bangladesh only)**
    - 1. Every purchase
      2. Total number in the last 6 months
      3. Consumption in last 7 days
- **Plausibility analysis based on impact pathways**
  - Is exposure to A&T interventions greater in A&T-intensive areas?
  - Are IYCF and nutrition knowledge, especially of key messages promoted by the program (“content tracers”) greater in A&T-intensive areas?
  - Are other behavioral determinants more conducive to behavior change in A&T-intensive areas?
  - Is intensity of intervention exposure associated with IYCF? practice/anthropometry outcomes, controlling for confounding factors?
- **Plausibility analysis based on underlying determinants of IYCF/anthropometry:**
  - Are there any systematic differences in underlying determinants of IYCF practices and anthropometric outcomes over time or across groups, such that *differential changes* in those underlying determinants over time offer alternative explanations for changes in IYCF or anthropometric outcomes? For example:
    - In Bangladesh, have there been changes in food security over time, and across groups, that might drive some of the changes in diet diversity?
    - In Vietnam, have there been differential changes in women’s work patterns over time and across groups, in ways that might shape EBF practices differently?
  - This analysis – assessing changes and then adjusting statistically for those underlying differences where changes are seen – will adjust for *time-variant* factors that changed over time.   The geographic clustering in the DID model only adjusts for time *invariant* changes over time.

# PUBLICATION PLANS

Research manuscripts related to the results of the evaluations in Bangladesh and Vietnam will be published separate for each set of major outcomes/sampling groups. Specifically, the following have been planned, in discussion with the implementing organizations and the funding agency:

1. One manuscript on exclusive breastfeeding and related secondary outcomes, combining findings from Bangladesh and Vietnam
2. One manuscript on complementary feeding, child anthropometric outcomes and related secondary outcomes, for Bangladesh.
3. One manuscript on complementary feeding, child anthropometric outcomes and related secondary outcomes, for Vietnam

# ANNEXES

# Annex 1. Description of modules in household questionnaire

| Module Name | Type of Data Collected |
| --- | --- |
| Household composition | - Basic demographic and socioeconomic data of the household members (name, relation to respondent mother, household head, sex, age, marital status, occupation, current school attendance, highest class has completed) |
| IYCF practices | - Data on core IYCF indicators based on WHO recommendation - Feeding prelacteal (immediately after birth or 3 days after birth) - Feeding colostrums - Feeding problems and care seeking |
| Child health history, feeding during illness and child appetite | - Illnesses during the previous two weeks (fever, cough/cold, fast breathing, diarrhea) - Feeding when child had diarrhea and recovered from illness - Child appetite |
| Pregnancy and postnatal care | - Antenatal care seeking - Using micronutrient supplement during pregnancy - Nutrition/IYCF counseling during ANC - Place of birth, Mode of delivery - Child birth weight - Support during delivery - BF advice/help during or immediately after delivery - Intention to use baby formula |
| Mother’s IYCF knowledge, beliefs andattitudes | - BF: Initiation, Exclusivity and Continuation - CF: Timing of introduction, Frequency, Quantity, Dietary diversity - Responsive feeding (Encourage children to eat) |
| Use of A&T community component program services by mother, father, grandmother (funds permitting) | - Exposure to home visits and counseling services:   - Awareness of and exposure to FLWs   - Received home visits   - Counseled during home visits   - Satisfaction with nutrition counseling services - Exposure to social mobilization |
| Use of micronutrient powder interventions | - Exposure to micronutrient powder interventions, including awareness, purchase and use of MNPs |
| Awareness, trial and adoption of sentinel practices | - Expose to information about BF, CF practices - Trial, adoption and influence factors |
| Child development | - Motor development scale - Language development scale - Early childhood development |
| Mass media | - Expose to TV, radio, - Viewing of advertisements/ information on IYCF - Specific TVC questions (aided recall)   - If viewed   - The most channels usually watch   - Main messages   - Activities follow up after watching the ads   - Opinion about messages and the ads |
| Behavioral Determinants | - Intention to adopt IYCF practices - Belief - Social Norms - Self-efficacy |
| Woman’s working condition, time constrains and decision making power | - Working condition - Time back to work and its influence on decision of BF - Self-efficacy about child feeding and workload - Perception about how people live within a household - Decision making power (purchasing power, using money) - Community supports - Social networks for health and IYCF information |
| Maternal physical and mental health | - Maternal stress (SRQ 20) |
| Household socio-economic status | - Household construction - List of assets |
| Household food security and diversity | - HFIAS - HDDS questions |
| Economic shocks | - Economic shocks have happened in the past 12 months that might have had an effect on your household |
| Anthropometry and anemia | - Height of both mother and child - Weight of both mother and child - Hemoglobin of both mother and child |
| Household consumption and expenditure | - The consumption and expenditure module will be done for households with children under two years of age as it is dominantly needed to assess expenditures on special foods for children and MNPs |

# Annex 2. Randomization of upazila for Alive & Thrive baseline study in BRAC EHP areas

| **District** | **Name of *upazilas*** | **Randomized to** |
| --- | --- | --- |
| ***Dhaka Division*** | | |
|  | Hossainpur | A&T |
|  | Pakundia | A&T |
|  | Sonargaon | No A&T |
|  | Araihazar | No A&T |
| ***Chittagong Division*** | | |
|  | Sonagaji | No A&T |
|  | Dagonbhuian | No A&T |
|  | Sonaimuri | A&T |
|  | Kompaniganj | A&T |
| ***Rajshahi Division*** | | |
|  | Nandigram | A&T |
|  | Kahalu | A&T |
|  | Parbatipur | No A&T |
|  | Chirirbandor | No A&T |
| ***Khulna-Barisal Division*** | |  |
|  | Damurhuda | A&T |
|  | Alamdanga | A&T |
|  | Bheramara | No A&T |
|  | Daulatpur | No A&T |
| ***Sylhet Division*** | | |
|  | Madhapur | A&T |
|  | Lakhai | A&T |
|  | Jointapur | No A&T |
|  | Goainghat | No A&T |

**References**

1. National Institute of Population Research and Training (NIPORT), Mitra and Associates, and ICF International. Bangladesh Demographic and Health Survey 2011. Dhaka, Bangladesh, and Calverton, Maryland, USA: 2013.

2. Saha KK, Frongillo EA, Alam DS, Arifeen SE, Persson LA, Rasmussen KM. Appropriate infant feeding practices result in better growth of infants and young children in rural Bangladesh. Am J Clin Nutr. 2008;87(6):1852-9. PubMed PMID: 18541577; PubMed Central PMCID: PMC2518656.

3. Roy SK, Fuchs GJ, Mahmud Z, Ara G, Islam S, Shafique S, et al. Intensive nutrition education with or without supplementary feeding improves the nutritional status of moderately-malnourished children in Bangladesh. J Health Popul Nutr. 2005;23(4):320-30. PubMed PMID: 16599102.

4. Haider R, Kabir I, Huttly SR, Ashworth A. Training peer counselors to promote and support exclusive breastfeeding in Bangladesh. J Hum Lact. 2002;18(1):7-12. PubMed PMID: 11845742.

5. Hossain SM, Duffield A, Taylor A. An evaluation of the impact of a US$60 million nutrition programme in Bangladesh. Health Policy Plan. 2005;20(1):35-40. doi: 10.1093/heapol/czi004. PubMed PMID: 15689428.

6. White H. Comment on contributions regarding the impact of the Bangladesh Integration Nutrition Project. 2005. doi: 10.1093/heapol/czi061.

7. Baker J, Sanghvi T, Hajeebhoy N, Martin L, Lapping K. Using an evidence-based approach to design large-scale programs to improve infant and young child feeding. Food and nutrition bulletin. 2013;34(3 Suppl):S146-55. PubMed PMID: 24261073.

1. Source: Field Operation Guideline. Alive & Thrive Project, BHP. May 2010 [↑](#footnote-ref-1)
