# Supplementary material for: Impacts on Breastfeeding Practices of At-Scale Strategies That Combine Intensive Interpersonal Counseling, Mass Media, and Community Mobilization: Results of Cluster-Randomized Program Evaluations in Bangladesh and Viet Nam
Source: PLoS Med. 2016 Oct 25;13(10):e1002159. doi: 10.1371/journal.pmed.1002159 (PMC5079648; doi:10.1371/journal.pmed.1002159)
Supplement: S3 Text — (DOCX) [file pmed.1002159.s016.docx]

| 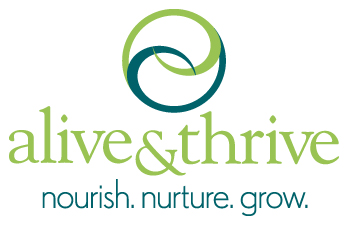  **S3 Text** |
| --- |
| **Survey Protocol for Impact Evaluation & Statistical Analysis** |
| **VIET NAM** |
|  |
| Phuong Nguyen  Purnima Menon  Lan Tran Mai  Rahul Rawat |
|  |

**Table of Contents**

[INTRODUCTION 3](#_Toc456971404)

[THE INTERVENTION 3](#_Toc456971405)

[Description of the Mat Troi Be Tho franchise model 3](#_Toc456971406)

[A&T mass media campaign 6](#_Toc456971407)

[EVALUATION OBJECTIVES 7](#_Toc456971408)

[EVALUATION METHODOLOGY 7](#_Toc456971409)

[Evaluation design 7](#_Toc456971410)

[Sample size and power calculation 9](#_Toc456971411)

[Data collection methods 10](#_Toc456971412)

[Training of personnel (CAPI training) 12](#_Toc456971413)

[Data management 12](#_Toc456971414)

[Data quality control 12](#_Toc456971415)

[STATISTICAL ANALYSIS PLAN 13](#_Toc456971416)

[Overall Approach 13](#_Toc456971417)

[Impact indicators 13](#_Toc456971418)

[Detailed Statistical Analysis Plan 14](#_Toc456971419)

[Estimation of impact of the A&T-intensive package of interventions, compared to the A&T non-intensive package, on IYCF practices 14](#_Toc456971420)

[Estimation of impact of the A&T-intensive package of interventions, compared to the A&T non-intensive package, on child growth outcomes 16](#_Toc456971421)

[ETHICAL APPROVAL 17](#_Toc456971422)

[PUBLICATION PLANS 17](#_Toc456971423)

[ANNEXES 18](#_Toc456971424)

[Annex 1. Description of modules in household questionnaire 18](#_Toc456971425)

[Annex 2. List of studied communes from 4 provinces 20](#_Toc456971426)

# INTRODUCTION

Alive & Thrive (A&T) is an initiative funded by the Bill & Melinda Gates Foundation to reduce undernutrition and death caused by suboptimal IYCF practices in three countries (Viet Nam, Bangladesh, and Ethiopia) over a period of six years (2009-2014). Alive & Thrive is led by FHI360 and is implemented through a partnership with Save the Children, GMMB, the International Food Policy Research Institute (IFPRI), and the University of California, Davis. In Vietnam, A&T is being implemented through a joint partnership with Save the Children, GMMB, the International Food Policy Research Institute (IFPRI), and the University of California, Davis. In Viet Nam, this initiative is implemented in close partnership with the Ministry of Health (MoH), the National Institute of Nutrition (NIN), provincial authorities, and other UN collaborators.

Scaling up interventions to improve key direct determinants of undernutrition in Vietnam, such as IYCF practices, are essential to accelerate progress toward reduction in undernutrition. Alive & Thrive’s portfolio of activities in this context has significant potential for impact. Rigorous impact evaluations are also essential to make contributions to the policy dialogue in Vietnam and elsewhere and A&T’s impact evaluation fills a significant knowledge gap by examining impact at scale, using a rigorous evaluation design.

# THE INTERVENTION

In Vietnam, A&T implemented three main activities: 1) an IYCF social franchise system at government health clinics, 2) a national mass media campaign, and 3) an advocacy strategy to support breastfeeding and complementary feeding. A total of 781 government health facilities at the province, district, and commune levels were supported to apply social franchising principles to deliver facility-based individual and group counseling. All facilities were required to meet minimum criteria including a standardized counseling room, trained staff, and availability of job aids and client materials. The program aimed to deliver 9 to 15 counseling contacts to each mother-child pair from the last trimester of pregnancy through the child’s first 2 years of life, including 8 breastfeeding contacts until the end of the first six months of life. Referrals, community mobilization activities, promotional print materials, and TV advertising were used to generate demand for preventive IYCF counseling services, a concept new to most families. In addition to counseling, there was a mass media component, consisting of a nationally broadcast campaign using TV and the digital space (internet and mobile phone applications). In A&T-intensive intervention areas, the mass media campaign also included additional public announcements through billboards and LCD screens. Advocacy at the national and provincial levels targeted the extension of paid maternity leave to 6 months, strengthening of the code of marketing of breast milk substitutes and improving provincial planning for IYCF and nutrition actions.

## Description of the Mat Troi Be Tho franchise model

A&T launched an innovative social franchise (SF) model – Mat Troi Be Tho (MTBT), (“The Little Sun” in English) – to provide high-quality nutrition consultation services to women and families at health facilities at all levels. The franchise activities were implemented in 15 of the 63 provinces in the country Implemented in cooperation with the Vietnamese government and select private clinics, franchises delivered a package of focused IYCF counseling services to pregnant women, lactating mothers, and their families, based on a franchise service package. Focused training and capacity building for healthcare workers have been undertaken to enable the health system to provide franchise services. Individualized services have been supported through mass media campaigns aimed at generating demand for franchise services and promoting optimal IYCF practices.

With aims to shape, create, and support the demand for IYCF services, A&T Viet Nam has developed the Mat Troi Be Tho franchise model with the following objectives:

-
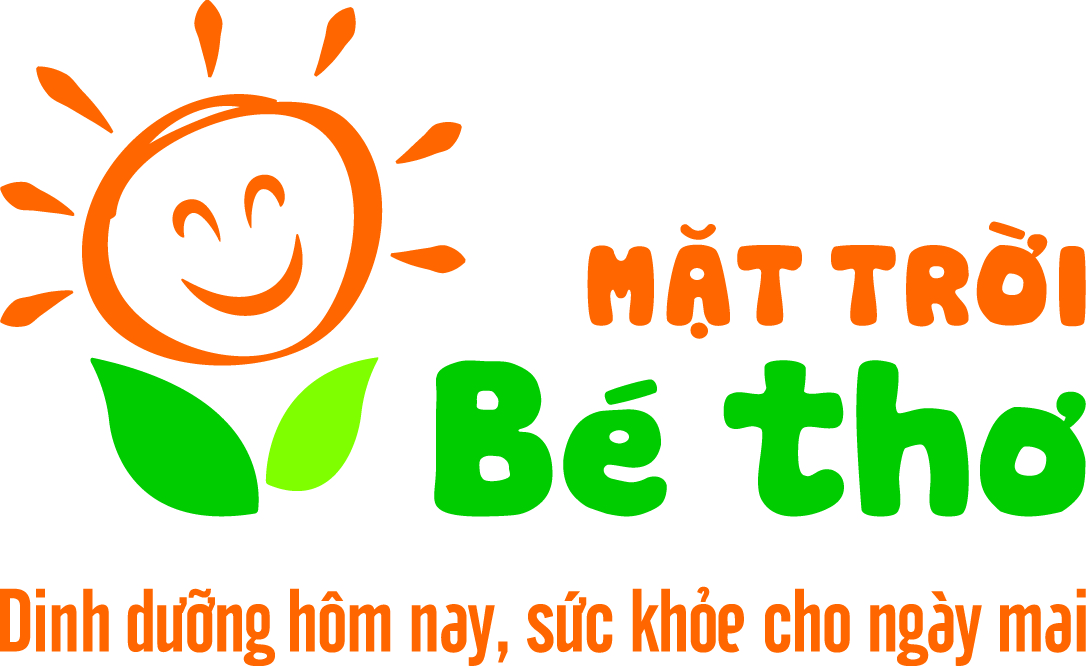
Provide good quality and relevant training to health workers who are in a position to encourage and support mothers to practice optimal IYCF;
- Standardize services and monitor them to ensure that counseling is uniform and of good quality;
- Respond to consumer demands for quality services and parents’ aspirations for their children; and
- Build on an existing, functional healthcare infrastructure and decentralized services to ensure sustainability.

Social franchising supports several objectives, targets, and strategies outlined in the government of Viet Nam’s National Nutrition Strategy, including integrating nutrition activities into primary healthcare, improving essential services, and upgrading the training of healthcare providers.

A&T Viet Nam, in partnership with NIN, functions as the National Franchisor with overall operational oversight and authority (Figure 1). Provincial health departments are selected as sub-franchisors. The IYCF franchise is designed to operate via varying levels and types of health facilities in Viet Nam. At the provincial level, franchise services will be made available through Reproductive Health Centers and public provincial hospitals. At the district level, services will be available at district hospitals and through maternity homes. The most comprehensive package of services will be offered at the commune level via commune health centers (CHCs). To be certified as a franchise facility, three criteria must be met: (1) upgraded room for providing IYCF counseling; (2) health staff and community workers trained in IYCF; and (3) availability of IYCF job aids and client materials. NIN is responsible for accrediting each franchise facility and awarding the Mat Troi Be Tho franchise brand for display at the facility. The IYCF franchise structure is illustrated in Figure 1.

The components offered will vary according to the type of facility. Services will be standardized but depending on the type of facility, some may not offer all IYCF services. The IYCF service delivery package (Table 1) is designed to encourage and enable women to practice optimal feeding for their infants from the time they are born until two years of age. The IYCF service delivery package is divided into five major components: 1) Exclusive breastfeeding (EBF) promotion, 2) EBF support, 3) EBF management, 4) CF education, and 5) CF management.

Figure 1. Mat Troi Be Tho franchise structure

**
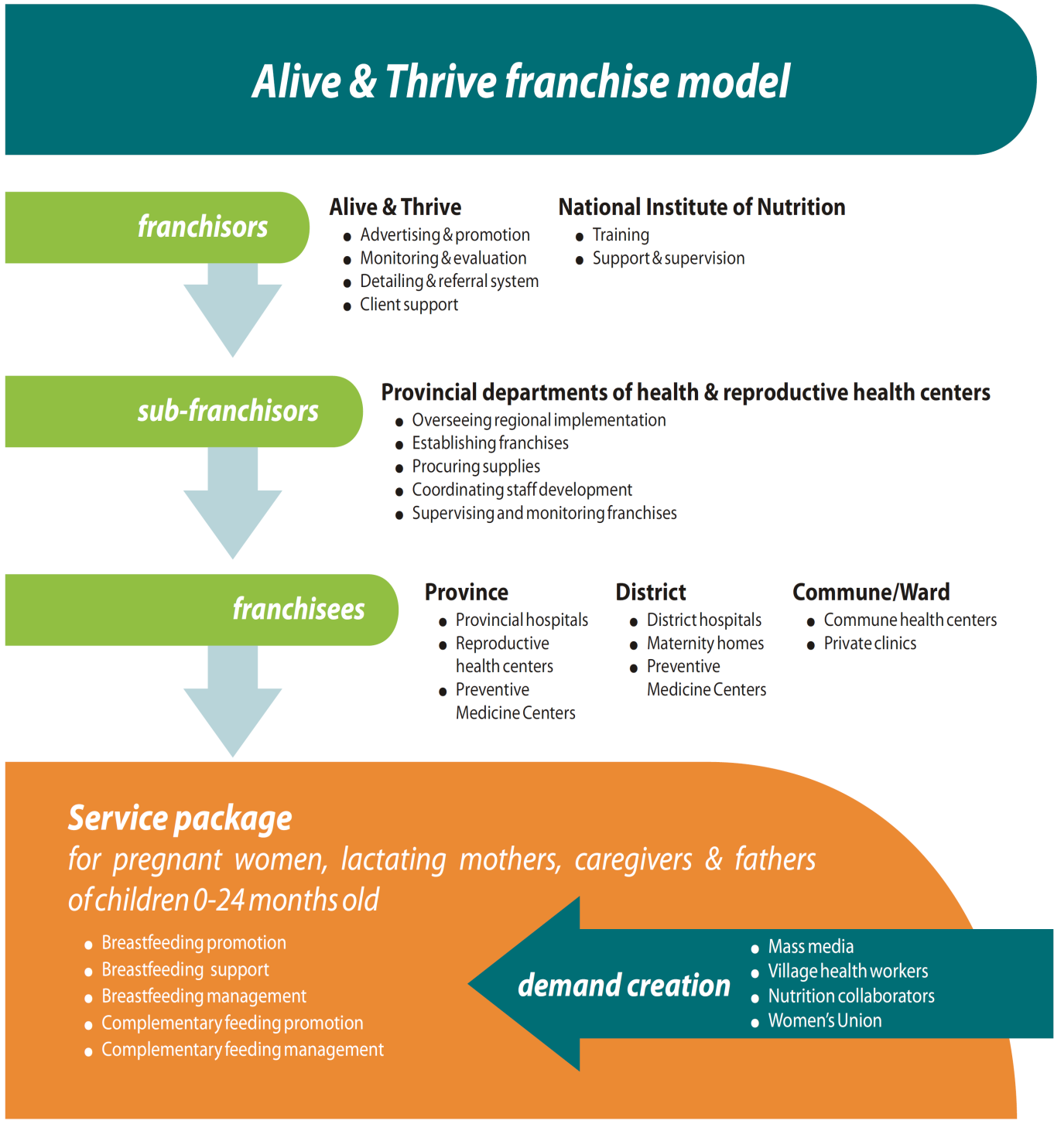
**

Table 1. Full IYCF package

| **Service Component** | **Method of Delivery** | **Timing** |
| --- | --- | --- |
| **Exclusive Breastfeeding Promotion** | 1 Group counseling/class  2 Individual counseling sessions | 3^rd^ trimester of pregnancy  3^rd^ trimester of pregnancy |
| **Exclusive Breastfeeding Support** | Personalized support | Time of delivery |
| **Exclusive Breastfeeding Management** | 2 Individual counseling sessions  2 Group counseling sessions/classes | 1 week postpartum  2 weeks postpartum  1–6 months postpartum |
| **Complementary Feeding Education** | 1 Individual counseling session | 4–6 months |
| **Complementary Feeding Management** | Combination of individual and group counseling sessions totaling 6 | One at 6–8 months  One at 9–12 months  Four at 12–24 months |

## A&T mass media campaign

 Alive & Thrive has launched mass media campaigns aimed at generating demand for franchise services and promoting optimal IYCF practices. These campaigns utilize multiple communication channels (Figure 2) including print materials and information communication technology to provide e- and tele-counseling services.

TV spots were broadcasted to achieve 80% national coverage. From September 2011 to August 2013, A&T launched 18 media bursts with 2,461 TV spots on 18 national and provincial channels. TV spots were complemented with promotion on the internet, including online TV spots on 24 popular websites in Viet Nam. In August 2011, A&T and NIN launched the Mặt Trời Bé Thơ website (www.mattroibetho.vn), a resource featuring educational short films and audio clips about IYCF. The website attracted 14,000,000 visits from August 2011 to August 2014. In addition to the TV and Internet campaign, A&T used posters, leaflets, booklets, village loudspeaker systems, and mobile technologies to expose target audiences to key messages. In 2014, a media campaign was launched with 78 percent national coverage, reaching 8.6 million women ages 19-35 [1].

Figure 2. Alive&Thrive’s mass media and behavior change communication activities in Viet Nam


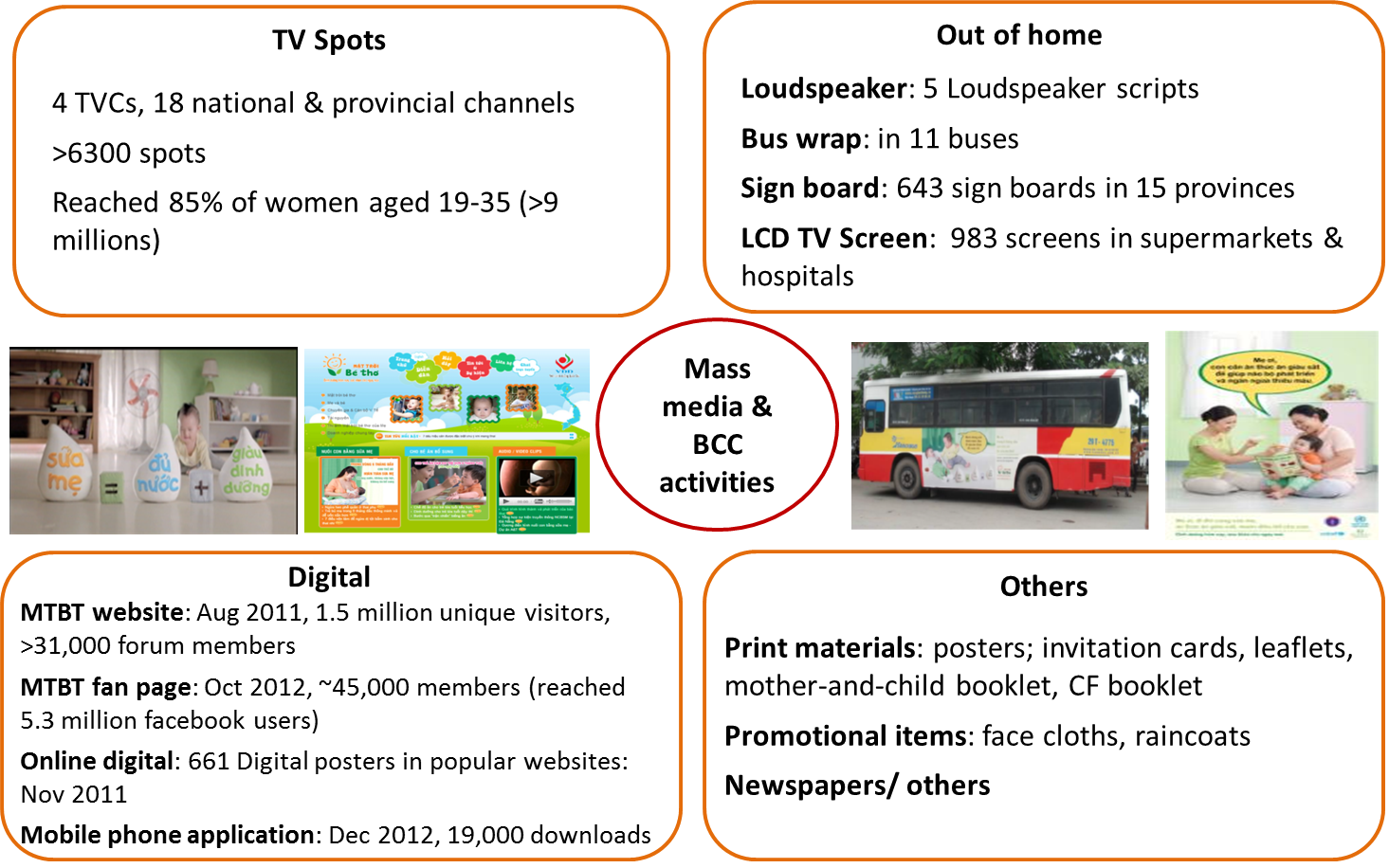


# EVALUATION OBJECTIVES

The impact evaluation is set up to address the following specific hypotheses:

1. Age-appropriate IYCF practices (EBF for 0-5.9 months or breastfeeding + age-appropriate complementary feeding for 6-23.9 months) will be better among children living in communes covered by the IYCF franchise services than those living in communes without the IYCF franchise services.
2. Children 2-5 years of age in communes covered by the IYCF franchise services will have lower levels of stunting than children 2-5 years of age in communes without the IYCF franchise services.

In addition to these objectives related to the IYCF franchise services, our design also allows us to assess the effects of the mass media campaign through the use of pre- and post-data collection at baseline and endline and the use of data on exposure to the mass media campaigns. We will use the extensive survey data to control for potential confounding in such analyses.

# EVALUATION METHODOLOGY

## Evaluation design

This impact evaluation used a cluster-randomized controlled design with repeated cross-sectional baseline and endline surveys (Figure 3) in the same communes within 4 provinces (Thai Nguyen, Thanh Hoa, Quang Ngai, and Vinh Long) [2], which are representative of the distinct ecological zones of the 15 provinces where A&T interventions were implemented (Figure 4). Forty communes were randomly assigned to either 1) A&T-intensive areas (A&T-I) (standard government health services, mass media (MM), and IYCF counseling at social franchises), or 2) A&T non-intensive areas (A&T-NI) (standard government health services and MM only). The mass media component was implemented in both A&T-I and A&T-NI. This rigorous impact evaluation design allowed us to detect the difference between the changes in key outcomes for intervention (A&T-I) and comparison (A&T-NI) groups between the baseline and endline surveys, thus providing difference-in-differences (DID) impact estimates.

In Vietnam, 15 provinces were selected for program implementation based on stunting levels, absence of other large organization working in nutrition, population density and representation of the different ecological regions covered by the initiative. Four rural provinces, representing 4 distinct ecological zones, were then selected for inclusion in the evaluation sample, and within these provinces, 10 rural districts, and two to six communes per district were selected for the evaluation based on the presence of a health center that met the eligibility criteria for the A&T franchise model; this ensured homogeneity across the sample. Communes were randomly assigned to either the *intensive* (20 communes) or *non-intensive* (20 communes) intervention. The randomization process was carried out using a simple public lottery system in the presence of local, district, and provincial health authorities as well as the program evaluators.

Figure 3. Impact Evaluation Design

**40 Communes**

**20 communes** allocated to **A&T *intensive*** intervention (standard government health services, intensified inter-personal counseling, community mobilization, and mass media campaign)

**20 communes** allocated to **A&T *non-intensive*** intervention (standard government health services, and mass media campaign)

**Cross-sectional baseline survey in 2010**

**Cross-sectional impact survey in 2014**

Figure 4. A&T Vietnam Program areas^1^


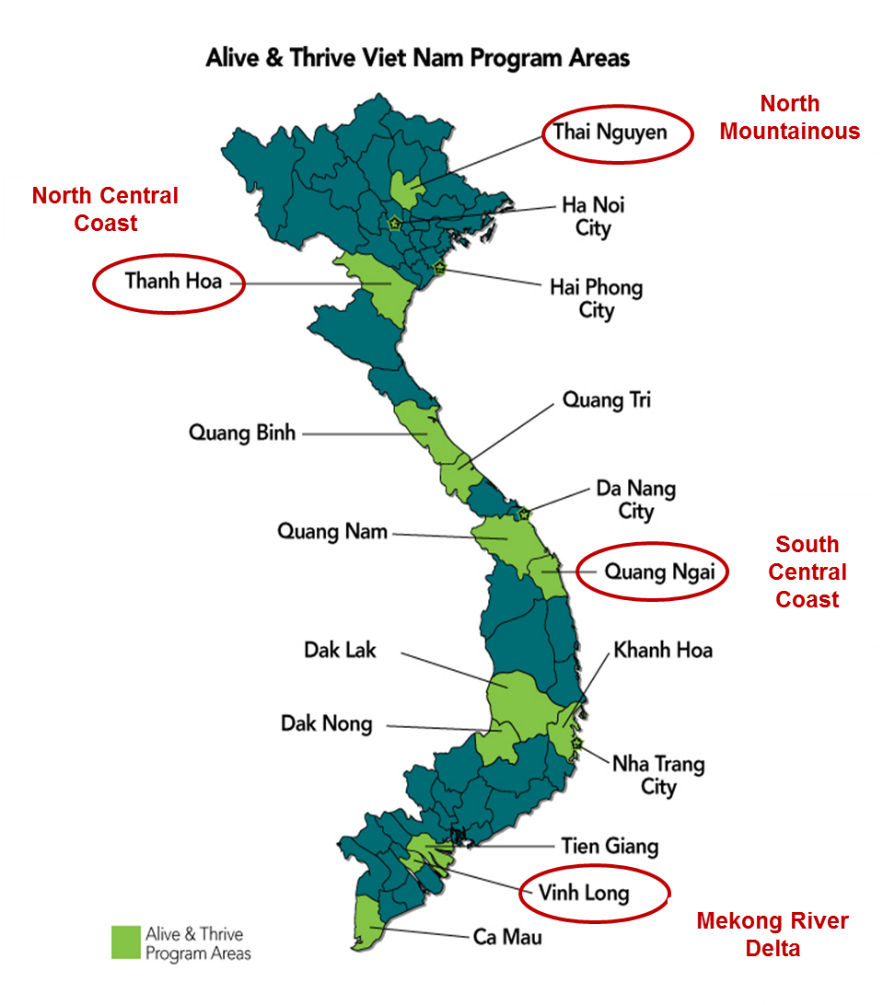


^1^Impact evaluation provinces indicated with red circles

## Sample size and power calculation

For the household survey, sample size was estimated based on the current rates of stunting, exclusive breastfeeding (EBF), and appropriate child feeding; the expected change in rates after intervention, power to detect those differences, and level of significance. As the intervention was randomized at the cluster level rather than the individual level, a clustering effect was also considered in the sample size and statistical power estimations. Each cluster was a group of communities served by a single commune health center (CHC). Detailed sample size and power calculations and actual sample sizes are presented in Table 2.

Based on these criteria, we chose a sample of mothers with children 0-5.9 months old for assessing impacts on EBF (500 in A&T-I and 500 in A&T-NI areas), mothers with children 6-23.9 months for assessing impacts on complementary feeding (500 in A&T-I and 500 in A&T-NI areas), and children 24-59.9 months (1000 children in each group) for assessing impacts on stunting as per the initial design. Even though anthropometric outcomes were measured in all three age groups, In addition, an oversample of all additional households with children aged 24-35.9 months was also included in the endline survey because slightly delayed implementation start-up meant that children in this age group would have even greater potential to have been fully exposed to the interventions than children 36 months and older.

Table 2. Sample size calculation survey

|  | **Baseline rate (%)** | **Detectable difference between groups at endline (percentage point (pp) or change in mean z-score)** | **Power** | **Intra-class correlation at baseline** | **Minimum sample size per group needed to detect differences with 80 percent power and alpha of 0.05** |
| --- | --- | --- | --- | --- | --- |
| Stunting (24-59.9m) | 24 | -6 pp* | 0.8 | 0.013 | 978 |
| HAZ (24-59.9m) | -0.9 ±1.2 | 0.2 | 0.8 | 0.026 | 993 |
| EBF (0-5.9m) | 18.4 | 10 pp | 0.8 | 0.037 | 420 |
| Minimum acceptable diet  (6-23.9m) | 57 | 10 pp | 0.8 | 0.037 | 700 |

For the CHC staff survey, 3 out of 5-6 staff members in each CHC were selected for interview. These included the CHC director, the midwife and the nurse in charge of nutrition activities. In A&T-I areas, these staff had been trained by A&T and participated in providing the MTBT counseling service. Thus in total, a maximum of 120 CHC staff were interviewed from the four provinces. In the franchise CHCs, 5 observations (3 individuals and 2 groups) were conducted of the first counseling sessions of the morning, and in non-franchise CHCs, 3 observations were conducted of the first counseling sessions of the morning. There was no specific IYCF counseling in A&T-NI areas, but there were some basic nutrition counseling for mothers during prenatal visit and some basic counseling on child feeding during health checkup for sick child.

## Data collection methods

Structured questionnaires were developed by IFPRI with inputs from A&T and translated into Vietnamese. Data was collected through four methods:

- Household questionnaire and anthropometric measurements of children and mothers
- Community questionnaire and facility assessment
- Observations of counseling sessions
- Frontline health worker (FHW) questionnaire

**Household questionnaire and anthropometric measurements of children and mothers**

The endline household questionnaire was developed using the baseline questionnaire and adapted to capture key program activities, particularly with regards to the use of A&T community services and exposure to mass media. The household questionnaire captured the main impact indicators (child anthropometry [3] and WHO-recommended IYCF indicators [4]); psychosocial/behavioral determinants (maternal IYCF knowledge, beliefs, self-efficacy and intentions); and client access and exposure to, and utilization of A&T services. It also captured influential underlying factors at the child level (child illness, developmental milestones, hygiene and hand washing), maternal characteristics (education, time constraints, and child care arrangement), as well as household characteristics (social economic status, economic shocks, and food security). The household questionnaire was administered to the mother of the index child chosen for the survey. A shorter version was administered to the father and grandmothers of the index child. In addition to the questionnaire-based data collection, anthropometric measurements (height and weight) were taken for mother and index child in each household.

Table 3 presents an overview of how the data collected in the household questionnaire was used in analysis and interpretation of impact results.

Table 3. Impact, process, and underlying factors captured by the endline household questionnaire

| **Type of data** | **Questionnaire modules/questions** | **How used** |
| --- | --- | --- |
| Impact indicators | Child anthropometry  WHO-recommended IYCF indicators | Change between baseline and endline used to assess trends in impact indicators. |
| Process data – client outcomes | Maternal IYCF knowledge, beliefs, intentions, and self- efficacy  Awareness, trial, and adoption of key recommended practices | Used to assess if IYCF knowledge shifted over the project period and whether awareness of key recommended behaviors improved. |
| Process data – client access, exposure, and utilization of services | Antenatal and postnatal care.  Exposure and use of MTBT services  Exposure to media messages | Used to assess if exposure to program channels was as expected. Allowed assessment of impact trends by levels of exposure to interventions. |
| Influencing/underlying factors – child level | Child illness and health  Child birth weight  Child age and gender | Used to assess if child-level factors modify influence of exposure to interventions. |
| Influencing/underlying factors – maternal level | Education  Time constraints  Physical and mental well-being  Status of women and maternal decision making power in the household | Used to control for underlying maternal factors and analyzed the influence of mothers’ constraints on their ability to benefit from A&T interventions. |
| Influencing/underlying factors – household level | Household SES (constructed from reported assets)  Household economic shocks  Household food security | Used to assess if the underlying factors shifted over the project period. In addition, it is used for controlling the impact of SES, economic shocks, and food security on IYCF impact indicators. |

**Community questionnaire and facility assessment**

The community questionnaire was designed to provide information on the following aspects of each commune:

- General characteristics of the commune: number of villages and population.
- Distance from the nearest major town.
- Natural disasters that occurred in the commune over the last year.
- Programs implemented in the commune over the last 3 years.
- Human resources at the CHC.
- Checklist of equipment at the CHC level franchise.

**Counseling observations**

Trained researchers will conduct observations of counseling sessions in order to assess the CHC health staffs’ competence and performance during IYCF counseling. The counseling observation checklist was developed based on the A&T/MTBT IYCF service delivery package guidelines. Each core franchise package component had its own specialized checklist that data collectors used to record how closely counselors followed the content as stipulated in the franchise guidelines. In addition to content coverage, data collectors used a checklist to assess counselors’ skills and counseling processes as a way to measure the quality of the counseling session.

**Frontline health worker questionnaire**

Data will be gathered on FHWs at baseline and endline to document changes in FHW performance over time and to understand of the impact pathways through which A&T objectives are met. Based on the literature, available validated scales, and our past experiences studying this issue in evaluations, we included in the A&T evaluation questionnaires the following eight domains that relate to FHWs and their performance, particularly in relation to delivery of the franchise IYCF service delivery package:

- Technical knowledge and skills related to IYCF
- Training exposure and needs assessment for future training
- Workload and time commitment (actual and perceived)
- Self-efficacy
- Job motivation and satisfaction
- Supervisory support
- Media exposure
- Basic demographic characteristics

## Training of personnel (CAPI training)

The data collection teams will be recruited and trained before traveling to the study provinces. Interviewer selection criteria includs 1) education and training background as well as experience in health-related fields; 2) understanding of and fluency in the local language; 3) ability to use computers/ tablets; 4) ability to observe and take notes during communication; and 5) ability to meet the time commitment. All data collectors were trained and assessed again after training to ensure that they met selection criteria.

The training course will be similar to the training offered during the baseline and process evaluation study. It consisted of instructions on the usage of the CSPro CAPI program on tablets, instructions regarding interviewing techniques and field procedures, a detailed review of items on the questionnaires, mock interviews between participants in the classroom, and practice interviews.

## Data management

Fieldwork and data processing activities will overlap. At the end of each day of fieldwork supervisors will copy and back up all the data files. These files will be merged into one file for each respondent type and then transferred directly from the field to ISMS headquarters. Once the fieldwork is completed in a province, the research team leader or assistant will merge all the data files for the province and export the fully labeled data to Stata. Variable and value labels will be checked against the questionnaires, and open-ended responses will be translated into English for data analysis. Datasets will be checked and cleaned by ISMS and sent to IFPRI headquarters for further review of range checks, logical consistency, missing values, and other data issues. All data validation and cleaning will be done using Stata 13 [5].

## Data quality control

The CSPro CAPI package provides a built-in method of quality control in the field via pre-programmed interactive checks that check for errors or missing data and manage the flow of the interview in real time. To ensure smooth functioning of the program in the field, the program will be piloted three times: once in the ISMS office and twice in a CHC in the Dong Anh district. The questionnaires and program will be revised as needed after piloting.

Additional quality control will be offered by field supervisors who will be responsible for checking the questionnaires immediately after submission by the interviewers. Field supervisors will do so by running the CSPro batch file, which identifies and reports the structure, value, and consistency errors in questionnaire data. The file enables field workers to produce a summary report of errors and change data values where necessary. Errors will be corrected on the spot by asking the respondent again. All files will be backed up to prevent data loss.

# STATISTICAL ANALYSIS PLAN

## Overall Approach

The overall approach to the A&T impact analysis involves two major steps:

1. Accurately **estimate the impact** of the A&T-intensive interventions on IYCF practices and anthropometric outcomes
2. **Enhance plausibility** of impact estimates through:
   1. **Examining social desirability bias** for IYCF practices, especially for those practices (EBF) that are susceptible to systematic reporting bias
   2. Assessment of **biological plausibility** of changes in outcomes based on age of initial exposure and duration of exposure
   3. ***Impact pathway analysis*** that documents exposure to A&T interventions
   4. Analysis of ***trends in underlying determinants*** of IYCF practices and child growth, to rule out alternative reasons for trends over time

The overall analysis will examine and adjust, as needed, both for time invariant characteristics that can have impact on outcomes (i.e., geographic clustering) and time variant determinants where important changes were seen over time (e.g., women’s work patterns, food security, etc.).

The impact on exclusive breastfeeding (EBF) will be assessed in children 0-5.9 months old. The impact on other IYCF practices and anemia will be assessed in children 6-23.9 months old and the impact on stunting will be assessed in children 24-59.9 months old.

Data analysis will be carried out according to the impact evaluation statistical analysis plan^[[1]](#footnote-1)^ that was developed in July 2014 and shared with A&T HQ, the Gates Foundation and partners. The main impact analyses will be conducted to estimate the impact of the A&T-intensive interventions on IYCF practices and anthropometric outcomes. Further analyses will be carried out to strengthen evidence of impact attributable to the program by constructing internal comparison groups and adjusting for characteristics that may have an effect on outcomes and to identify factors that may have contributed to changes in child growth.

## Impact indicators

A&T’s core impact indicators are the 8 WHO-recommended IYCF indicators and child stunting. We will, however, examine impacts on all anthropometric indices - height-for-age, weight-for-age and weight-for-height. The indicators are depicted below:

**Table 4: Impact indicators**

| **Indicator** | **Age group (mo)** | **Remarks** |
| --- | --- | --- |
| ***Breastfeeding practices*** |  |  |
| Early initiation of breastfeeding | 0-23.9 |  |
| Exclusive breastfeeding under 6 months | 0-5.9 | We will also examine questions included in the endline survey that address social desirability bias |
| Continued breastfeeding at 1 year | 12-15.9 |  |
| ***Complementary feeding*** |  |  |
| Introduction of solid, semi-solid food, or soft food | 6-8.9 |  |
| Minimum diet diversity | 6-23.9 |  |
| Minimum meal frequency | 6-23.9 |  |
| Minimum acceptable diet | 6-23.9 |  |
| Consumption of iron-rich food | 6-23.9 |  |
| ***Anthropometry*** |  |  |
| HAZ | 24-59.9 | We will also examine impacts on HAZ among potentially fully exposed children (24-35.9 mo.) |
| Stunting | 24-59.9 | We will also examine impacts on HAZ among potentially fully exposed children (24-35.9 mo.) |

## Detailed Statistical Analysis Plan

Two broad sets of analyses will be conducted for this evaluation; these are:

1. Estimation of  **impact of the A&T-intensive package of interventions, compared to the A&T non-intensive package, on IYCF practices**
   1. Estimation of main **impact of A&T-intensive interventions on IYCF practices** using difference-in-difference estimates
   2. A series of additional analyses to assess the **plausibility** of identified impacts. These additional analyses included the following:

Plausibility analysis 1: Examining **social desirability bias** and other potential biases in reported IYCF practices, especially for those practices (i.e., EBF) that are presumed to be susceptible for systematic reporting bias.

Plausibility analysis 2: Analysis of **changes along program impact pathways**, particularly impact of A&T-intensive interventions on maternal psychosocial behavioral determinants related to IYCF practices.

Plausibility analysis 3: **Dose-response analysis** between program exposures and IYCF practices, thereby creating internal comparison groups to test program effects with greater degree of confidence.

1. Estimation of **impact of the A&T-intensive package of interventions, compared to the A&T non-intensive package, on child growth outcomes**
   1. Estimation of main **impact of A&T-intensive interventions on child growth outcomes** using difference-in-difference estimates
   2. A series of additional analyses to assess the plausibility of identified impacts. These additional analyses included the following:

Plausibility analysis 1: Analysis of change in stunting prevalence among children in **high potential-for-impact age group** (24-35.9 months).

Plausibility analysis 2: **Dose-response analysis** between program exposures and child growth outcomes.

Plausibility analysis 3: Analysis of **change in underlying determinants of stunting** over time

#### Estimation of impact of the A&T-intensive package of interventions, compared to the A&T non-intensive package, on IYCF practices

1. **Main difference-in-difference analyses to accurately estimate the impact of A&T interventions on IYCF practices**

The baseline and endline household datasets will be merged into a single dataset with a time period identifier. The difference-in-differences (DID) (or double-difference) method will be applied to assess the difference between the change in the outcomes for intervention (A&T- I) and comparison (A&T- NI) groups in the baseline and endline, first accounting for geographic clustering only (pure intent-to-treat (ITT), referred to henceforth as “pure ITT”), then additionally accounting for the child’s sex and age (“adjusted ITT”). The adjusted ITT for child age and sex are the preferred models for estimating impact on anthropometric outcomes. The impact estimates will yield (1) percentage point changes for IYCF practices and stunting, wasting, and underweight; and (2) change in mean Z-scores for height-for-age (HAZ), weight-for-age (WAZ), and weight-for-height (WHZ). Analyses will focus on the following:

- ***Pure* i*ntent-to-treat*** analyses based on the original evaluation design and original age groups, using child-level data and fixed-effect analysis to achieve double difference estimates. The fixed-effect analysis accounts for the commune as cluster. Further adjustment for geographic clustering will be done at district level.
- ***Adjusted intent-to-treat* analysis** using data from the original *intent-to-treat* age groups but now adjusting for child age and child sex. In future analyses, controls will be included for other variables where changes over time might be different between the A&T-intensive and A&T-non-intensive groups and variables that may have changed significantly over time.

For the analyses noted above, regression models will be run using the *diff* and *xtreg* commands in Stata 13, adjusting for geographic clustering at the commune and district-level. Both approaches to the regression modeling provided estimates of impact that capture changes over time and between groups. Results will be reported at P<0.05, P<0.01, and P<0.001 significance levels.

1. **Plausibility analysis of identified impacts on IYCF practices:**

***Plausibility analysis 1: Examining social desirability bias and other potential bias***

Recognizing the potential role of social desirability bias in influencing reporting of nutrition behaviors such as IYCF practices [6], we will examine this within the A&T impact analysis in the following ways:

- A brief set of questions has been specifically designed to assess the extent to which respondents were likely to report behaviors based on their desire for social approval, i.e., social desirability bias. This is an attempt to capture the tendency of respondents to answer questions in a manner that is socially desirable, so as to be viewed favorably by others. Social desirability bias can interfere with the interpretation of average tendencies as well as individual differences. Previous attempts have been made to measure and quantify the problem of social desirability bias. This literature was reviewed and a subset of items was adapted from Reynolds [7]. We administered this subset of five questions to assess social desirability bias based on a review of the most commonly used measures, including the Marlowe-Crowne scale. Based on internal review of scale questions and local pretesting, a set of 5 questions were included in the questionnaires and administered together with the primary measures of the study. Based on responses to the questions, a 5-point social desirability scale measure will be developed. The key assumption is that respondents who answer in a socially desirable manner on the scale may also be responding to other questions (e.g., those related to IYCF practices) in a manner they think is socially desirable.
- The social desirability score (SDS) will be created by adding up the number of socially desirable answers, out of the 5 questions in the module. Therefore, the SDS ranges from 0-5, with a SDS of 0-2 being considered as a low score, 3 as medium score, 4 as high and 5 as very high. Three types of analyses using this scale will be conducted: (1) Mean SDS by program group will be calculated to check for differences in social desirability bias between the two A&T study groups; (2) The EBF practices were based on categories of the SDS, to assess whether reported practice varied by score on the SDS; and (3) A set of regressions will be run with each of the core IYCF practices as dependent variables to test the interaction between the SDS and the A&T program variable, to assess whether or not social desirability bias differentially affected the impact of the A&T-intensive model on IYCF practices.

***Plausibility analysis 2: Analysis of changes along program impact pathways***

In this set of analyses, findings related to program implementation and exposure to A&T interventions at the household level will be assessed along the impact pathway by assessing the following using the available data from endline survey:

- Is exposure to A&T interventions greater in A&T-intensive areas?
- Are IYCF and nutrition knowledge of and beliefs about key behaviors promoted by the program (“content tracers”) greater in A&T-intensive areas?

***Plausibility analysis 3:*** ***Dose-response analysis between program exposure and IYCF practices***

We will examine the association between the exposure and intensity of exposure to the diverse intervention platforms associated with IYCF practices and anthropometric outcomes used by A&T.  For exposure, we will compare groups representing exposure (or not) to different program elements.  For intensity, we will use dose-response analyses between degrees of exposure to various program elements to IYCF practices at endline. A&T-intensive and non-intensive groups will be analyzed separately throughout most of the analysis, however, an overall model with the combined groups will also be used to assess any relevant overall effects.

#### Estimation of impact of the A&T-intensive package of interventions, compared to the A&T non-intensive package, on child growth outcomes

1. **Main difference-in-difference analyses to accurately estimate the impact of A&T interventions on child growth:**

We will use the same method as described in the main impact for IYCF indicators.

1. **Plausibility analysis of identified impacts on child growth:**

***Plausibility analysis 1: Analyses on high potential for exposure age group (24-35.9 months)***

In this analysis, anthropometric outcome outcomes will be examined by child age, using data on children in age groups deemed to be those where children were likely exposed to the A&T interventions from birth until 24 months of age, which were children in the age range of 24-35.9 months (over-sample).

***Plausibility analysis 2:*** ***Dose-response analysis between program exposure and child growth outcomes***

The exposure/dose-response analysis for stunting will be carried out choosing the age groups which are most likely to be exposed to the A&T interventions based on known information about the implementation timelines of A&T interventions, specifically interpersonal counseling. Thus, this analysis will combine the dose-response analysis with the idea of “potential to benefit”.

***Plausibility analysis 3: Analysis of change in determinants of stunting over time***

Analysis of trends in underlying determinants of child growth outcomes will be carried out to infer potential alternative explanations for outcomes. Trends in these underlying factors will be taken into consideration in examining the determinants of child growth over time.

- Are there any systematic differences in underlying determinants of IYCF practices and anthropometric outcomes over time or across groups, such that differential changes in those underlying determinants over time offer alternative explanations for changes in IYCF or anthropometric outcomes? For example: In Vietnam, have there been differential changes in food security over time, women’s work patterns over time and across groups, in ways that might shape EBF practices differently?
- This analysis – assessing changes and then adjusting statistically for those underlying differences where changes are seen – will adjust for *time-variant* factors that changed over time. The geographic clustering in the DID model only adjusts for time *invariant* changes over time.

# ETHICAL APPROVAL

Approval for the study has been obtained from the institutional review board at IFPRI, and the Vietnam Union of Science and Technology Association. All mothers of study children were provided with detailed information about the study in writing and verbally at recruitment. Written informed consent will be obtained from mothers/caregivers in Vietnam.

# PUBLICATION PLANS

Research manuscripts related to the results of the evaluations in Bangladesh and Vietnam will be published separate for each set of major outcomes/sampling groups. Specifically, the following have been planned, in discussion with the implementing organizations and the funding agency:

1. One manuscript on exclusive breastfeeding and related secondary outcomes, combining findings from Bangladesh and Vietnam
2. One manuscript on complementary feeding, child anthropometric outcomes and related secondary outcomes, for Bangladesh.
3. One manuscript on complementary feeding, child anthropometric outcomes and related secondary outcomes, for Vietnam

# ANNEXES

## Annex 1. Description of modules in household questionnaire

| Mother’s Modules | Module Name | Type of Data Collected |
| --- | --- | --- |
| Module 1 | Household composition | - Basic demographic and socioeconomic data of the household members (name, relation to respondent mother, household head, sex, age, marital status, occupation, current school attendance, highest class has completed) |
| Module 2 | IYCF practices | - Data on core IYCF indicators based on WHO recommendation - Feeding prelacteal (immediately after birth or 3 days after birth) - Feeding colostrums - Feeding problems and care seeking |
| Module 3 | Child health history, feeding during illness and child appetite | - Illnesses during the previous two weeks (fever, cough/cold, fast breathing, diarrhea) - Feeding when child had diarrhea and recovered from illness - Child appetite |
| Module 4 | Pregnancy and postnatal care | - Antenatal care seeking - Using micronutrient supplement during pregnancy - Nutrition/IYCF counseling during ANC - Place of birth, Mode of delivery - Child birth weight - Support during delivery - BF advice/help during or immediately after delivery - Intention to use baby formula |
| Module 5 | Mother’s IYCF knowledge attitudes | - BF: Initiation, Exclusivity and Continuation - CF: Timing of introduction, Frequency, Quantity, Dietary diversity - Encourage children to eat |
| Module 6 | Use of A&T community component program services | - Expose to nutrition counseling services:   - Awareness of and exposure to franchise services   - Receive invitation card of franchise   - Using franchise services and influence factors   - Using other nutrition counseling services   - Getting counseling information from counseling services   - Weight and height measurement during nutrition counseling services   - Payment for counseling services   - Expose to IEC materials and promotional items   - Satisfaction with nutrition counseling services - Attend to child feeding groups or meeting |
| Module 7 | Awareness, trial and adoption of sentinel practices | - Expose to information about BF, CF practices - Trial, adoption and influence factors |
| Module 8 | Child development | - Motor development scale - Language development scale - Early childhood development |
| Module 9 | Mass media | - Expose to TV, radio, commune loudspeaker and internet - Viewing of advertisements/ information on infant formula and child feeding - Specific TVC questions   - If viewed   - The most channels usually watch   - Main messages   - Activities follow up after watching the ads   - Opinion about messages and the ads |
| Module 10 | Behavioral Determinants | - Intention to adopt IYCF practices - Belief - Social Norms - Self-efficacy |
| Module 11 | Woman’s working condition, time constrains and decision making power | - Working condition - Time back to work and its influence on decision of BF - Self-efficacy about child feeding and workload - Perception about how people live within a household - Decision making power (purchasing power, using money) - Community supports - Social networks for health and IYCF information |
| Module 12 | Maternal physical and mental health | - Maternal stress (SRQ 20) |
| Module 13 | Household socio-economic status | - Household construction - List of assets |
| Module 14 | Household food security and diversity | - HFIAS - HDDS questions |
| Module 15 | Economic shocks | - Economic shocks have happened in the past 12 months that might have had an effect on your household |
| Module 16 | Anthropometry | - Height of both mother and child - Weight of both mother and child |

## Annex 2. List of studied communes from 4 provinces

| **Province** | **District/city** | **CHC in A&T Intensive Areas** | **CHC in A&T Non-Intensive Areas** |
| --- | --- | --- | --- |
| Thanh Hoa | Hau Loc | Thinh Loc | Loc Tan |
|  |  | Dong Loc | Chau Loc |
|  | Tinh Gia | Hai An | Tan Dan |
|  |  | Ngoc Linh | Thanh Son |
|  |  | Tan Truong | Truong Lam |
|  | Cam Thuy | Cam Phong | Cam Son |
|  |  | Cam Binh | Cam Ngoc |
| Thai Nguyen | Dong Hy | Trai Cau town | Song Cau town |
|  |  | Linh Son | Quang Son |
|  | Pho Yen | Phuc Tan | Dac Son |
|  |  | Bai Bong town | Bac Son town |
| Vinh Long | Vinh Long city | Tan Hoi | Tan Ngai |
|  | Tam Binh | Long Phu | My Loc |
|  | Vung Liem | Trung Hiep | Trung An |
|  |  | Hieu Thanh | Trung Hieu |
| Quang Ngai | Binh Son | Binh Minh | Binh My |
|  |  | Binh Dong | Binh Thanh |
|  |  | Binh Long | Binh Trung |
|  | Tu Nghia | Song Ve town | La Ha town |
|  |  | Nghia Phuong | Nghia Thuan |

**References**

1. A&T. BCC 2014 Update. 2014.

2. Menon P, Rawat R, Ruel M. Bringing rigor to evaluations of large-scale programs to improve infant and young child feeding and nutrition: The evaluation designs for the Alive & Thrive initiative. Food and nutrition bulletin. 2013;34(3):S195-S211.

3. WHO. The WHO Child Growth Standards. <http://www.who.int/childgrowth/standards/en/>. 2010.

4. WHO. Indicators for assessing infant and young child feeding practices. Part 2: Measurements. <http://whqlibdoc.who.int/publications/2010/9789241599290_eng.pdf:> World Health Organization: Geneva; 2008.

5. StataCorp. Stata Statistical Software: Release 11. College Station, Texas 77845 USA. Copyright 2009 StataCorp LP. 2009.

6. Hebert JR, Clemow L, Pbert L, Ockene IS, Ockene JK. Social desirability bias in dietary self-report may compromise the validity of dietary intake measures. International journal of epidemiology. 1995;24(2):389-98. PubMed PMID: 7635601.

7. Reynolds W. Development of reliable and valid short forms of the marlowe-crowne social desirability scale. Journal of Clinical Psychology. 1982;38(1):119-25.

1. # Impact Analysis Plan – Draft for Discussion. July 16, 2014.

   [↑](#footnote-ref-1)
