# Supplementary material for: Impacts on Breastfeeding Practices of At-Scale Strategies That Combine Intensive Interpersonal Counseling, Mass Media, and Community Mobilization: Results of Cluster-Randomized Program Evaluations in Bangladesh and Viet Nam
Source: PLoS Med. 2016 Oct 25;13(10):e1002159. doi: 10.1371/journal.pmed.1002159 (PMC5079648; doi:10.1371/journal.pmed.1002159)
Supplement: S4 Text — (DOCX) [file pmed.1002159.s017.docx]

# **
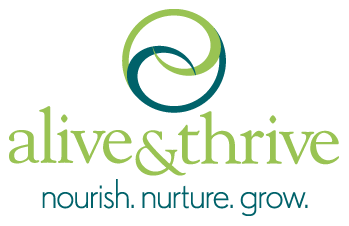
**

# **S4 Text**

# **Measurement, Learning and Evaluation**

# **Impact Analysis Plan^[[1]](#footnote-1)^,^[[2]](#footnote-2)^**

## **Overall approach**

The overall approach to the A&T impact analysis involves two major steps:

1. Accurately **estimate the impact** of the A&T-intensive interventions on IYCF practices and anthropometric outcomes
2. **Enhance plausibility** of impact estimates through:
   1. **Examining social desirability bias** for IYCF practices, especially for those practices (EBF) that are susceptible to systematic reporting bias
   2. Assessment of **biological plausibility** of changes in outcomes based on age of initial exposure and duration of exposure
   3. ***Impact pathway analysis*** that documents exposure to A&T interventions
   4. Analysis of ***trends in underlying determinants*** of IYCF practices and child growth, to rule out alternative reasons for trends over time

The overall analysis will examine and adjust, as needed, both for time invariant characteristics that can have impact on outcomes (i.e., geographic clustering) and time variant determinants where important changes were seen over time (e.g., women’s work patterns, food security, etc.).

## **Summary of impact evaluation designs and age groups for impact analysis of each indicator**

### **Bangladesh**

#### Overview of evaluation design

The evaluation design for the assessment of the impact of the A&T intervention by BRAC in Bangladesh uses a cluster-randomized design, with repeated cross-sectional surveys at baseline and endline. Twenty upazila were randomly assigned to either to A&T intervention (10 upazila) or comparison areas (10 upazila). This endline survey in 2014 will be conducted in these twenty impact evaluation upazila, 4 years after the baseline survey was conducted in 2010.

The impact on exclusive breastfeeding (EBF) will be assessed in children 0-5.9 months old. The impact on other IYCF practices will be assessed in children 6-23.9 months old and the impact on stunting will be assessed in children 24-47.9 months old.

#### Sample sizes

**Table 1: Sample sizes for Alive & Thrive Endline Survey 2014**

| **Levels** | **Respondents** | **Child age group** | **Sample size per study arm** | **Total sample size** | **Notes** |
| --- | --- | --- | --- | --- | --- |
| Household | Mothers | 0-5.9 months | 500 | 1000 |  |
|  |  | 6-23.9 months | 500 | 1000 |  |
|  |  | 24-47.9 months | 1100 | 2200 |  |
|  | | 12-17.9 months | 500 | 1000 | Follow-up children from 2013 who were 0-5.9 months |
| Total |  | | **2100** | **5200** |  |
|  | Fathers |  |  | 1300 |  |
|  | Grandmothers |  |  | 700 |  |
| FLW | SS/PS |  |  | 300 |  |
|  | PK |  |  | 100 |  |
|  | SK |  |  | 100 |  |
|  | Village doctors |  |  | 200 |  |
| **Total** |  |  |  | **1400** |  |
| Community | Key informants |  | 100 villages | 200 villages |  |

### **Vietnam**

#### Overview of evaluation design

This program evaluation uses a cluster-randomized controlled design with repeated cross-sectional baseline and endline surveys. A total of 40 CHCs in these four provinces were selected at baseline and randomly assigned to 1) standard Government Service and 2) IYCF franchise + Standard Government Service. The baseline survey was done in June to August 2010, and an endline survey is planned to be conducted in June to August 2014. This rigorous impact evaluation design will allow us to detect the difference between the change in the outcomes for intervention (A&T) and comparison (non-A&T) groups between the baseline and endline survey, thus providing difference-in-differences impact estimates.

The impact on exclusive breastfeeding (EBF) will be assessed in children 0-5.9 months old. The impact on other IYCF practices will be assessed in children 6-23.9 months old and the impact on stunting will be assessed in children 24-59.9 months old. Stunting effects will additionally be examined among children 24-35.9 months of age, considering these children resided in areas where they could have been exposed to franchise services from birth until 23.9 months of age.

#### Sample sizes

**Table 2: Sample size selected for household endline survey by child age group**

| **Level** | **Respondent** | **Child age group** | **Total sample size** | **Notes** |
| --- | --- | --- | --- | --- |
| Household | Mothers | 0-5.9 months | 1,000 |  |
|  |  | 6-11.9 months | 350 |  |
|  |  | 12-17.9 months | 800 | Follow-up children from 2013 who were 0-5.9 months |
|  |  | 18-23.9 months | 350 |  |
|  |  | 24-35.9 months | 2,000 | Oversample approximately 1300 children 24-35.9 m |
|  |  | 36-59.9 months | 1,300 |  |
|  | | **Total** | **5,800** |  |
| **FLW** | **CHC staff** |  | **120** |  |

## **Impact indicators**

A&T’s core impact indicators are the 8 WHO-recommended IYCF indicators and child stunting. We will, however, examine impacts on all anthropometric indices – height-for-age, weight-for-age and weight-for-height. The indicators are depicted below:

| **Indicator** | **Age group (mo)** | **Remarks** |
| --- | --- | --- |
| ***Anthropometry*** |  |  |
| HAZ | 24-59.9 | We will also examine impacts on HAZ among potentially fully exposed children (24-35.9 mo.) |
| Stunting | 24-59.9 | We will also examine impacts on HAZ among potentially fully exposed children (24-35.9 mo.) |
| ***IYCF practices*** |  |  |
| Early initiation of breastfeeding | 0-23.9 |  |
| Exclusive breastfeeding under 6 months | 0-5.9 | We will also examine questions included in the endline survey that address social desirability bias |
| Continued breastfeeding at 1 year | 12-15.9 |  |
| Introduction of solid, semi-solid food, or soft food | 6-8.9 |  |
| Minimum diet diversity | 6-23.9 |  |
| Minimum meal frequency | 6-23.9 |  |
| Minimum acceptable diet | 6-23.9 |  |
| Consumption of iron-rich food | 6-23.9 |  |

## **Detailed Analysis Plan: Bangladesh and Vietnam (cluster randomized evaluation designs)**

1. **Impact estimates**

Impact estimates in Bangladesh and Vietnam, the two countries with cluster-randomized evaluation designs, will be double difference estimates of changes in key impact indicators. This takes into account changes over time between baseline and endline in the intervention and comparison groups, and differences between groups at these times. The impact estimates will yield (1) **percentage point and percentage changes** for IYCF practices and stunting, wasting, and underweight; and (2) change in **mean Z scores** for height for age, weight for age, and weight for height.

Analyses will focus on the following:

- ***Pure* *Intent-to-treat*** analyses based on the original evaluation design and original age groups, using child-level data and fixed-effects analysis to achieve double difference estimates. The fixed-effects analysis will account for village, the smallest level repeated between baseline and endline. Further adjustment for geographic clustering at provincial/divisional, district and commune/subdistrict level will be done using random- or fixed-effects to adjust for clustering if needed; the need for this is not likely because of the accounting of variation among villages.
- ***Pure intent to treat in high potential for exposure age groups* –** this analysis will focus on the stunting indicator and capture impact estimates in an age group of children who are mostly likely, because of their age, to have been exposed to A&T’s interventions early in infancy and for the longest duration. This is the 24-35 month age group in both Bangladesh and Vietnam. We will examine the pattern of HAZ across the full 0-59 month age range, and look at impacts on stunting beginning at the age when HAZ begins leveling off i.e. possibly beginning at 18 months of age. We will use fixed-effects regression models to adjust for village clustering as above.
- ***Adjusted intent-to-treat* analysis** using data from the original ITT age groups but now adjusting for child age, child sex and other variables that might be different between the A&T-intensive and A&T-non-intensive age groups. We will use random- or fixed-effects regression models to adjust for geographic clustering.

1. **Plausibility analysis 1 – Examining social desirability bias**

Recognizing the potential role of social desirability bias in influencing reporting of nutrition behaviors, we plan to examine this within the A&T impact analysis in the following ways:

- Examine results of a diverse set of “trick” questions in the surveys that try and capture ways of triangulating findings on EBF and diet diversity (given both of these rely on the 24-hr recall of foods fed to the kids and the mothers might either under or over report in the recall instrument, depending on the child’s age group).  Estimates of IYCF indicators separately for groups that appear to be “fixing” their 24 hour recall responses. Examples include:
  - Asking elsewhere in the questionnaire (not the IYCF module) about availability of formula, feeding bottles, special bowl for the child, etc. “Child-specific” assets have been added into the household asset module, for instance, and we ask about books, toys and special feeding materials for the child.
  - In Bangladesh, include formula, tinned milk powder, and baby bottles in the consumption expenditure module
  - Ask to see all the things they use when they are feeding the babies, maybe – all bowls, plates, cups, bottles – and note this in a short observation
- Triangulate the 24-hr recall data from interviews with mothers by also examining data from the grandmother and father what foods the child ate in the last 24 hours. Triangulate in the analysis the concordance here.
- Examine data on stool frequency and consistency and urine frequency in the illness module (likely not very reliable because bigger threat to EBF is water in most places)
- Examine data from questions on social desirability tendencies

*Note: there is currently no way to correct for any situations where social desirability bias appears to be strong from the above assessment approach but we will report briefly the findings from these analyses as part of any impact papers.*

1. **Plausibility analysis 2 - based on biological plausibility and implementation plausibility (exposure to A&T interventions)**
   1. Examine **anthropometry outcomes by child age (**biological plausibility**) and duration of exposure** (intent to treat analysis for implementation plausibility).

- **Exposure variables** will include the following
  - FLW contact (use the question regarding # of contacts within the last 6 months))
  - Mass media exposure (use both the question of recall of ever having seen specific ads, with recall of at least x number of messages in the ad, or select messages )
  - Franchise contact (VN)
- **Plausibility analysis based on impact pathways**
  - Is exposure to A&T interventions greater in A&T-intensive areas?
  - Are IYCF and nutrition knowledge, especially of key messages promoted by the program (“content tracers”) greater in A&T-intensive areas?
  - Are other behavioral determinants more conducive to behavior change in A&T-intensive areas?
  - Is intensity of intervention exposure associated with IYCF? practice/anthropometry outcomes, controlling for confounding factors?
- **Plausibility analysis based on underlying determinants of IYCF/anthropometry:**
  - Are there any systematic differences in underlying determinants of IYCF practices and anthropometric outcomes over time or across groups, such that *differential changes* in those underlying determinants over time offer alternative explanations for changes in IYCF or anthropometric outcomes? For example:
    - In Bangladesh, have there been changes in food security over time, and across groups, that might drive some of the changes in diet diversity?
    - In Vietnam, have there been differential changes in women’s work patterns over time and across groups, in ways that might shape EBF practices differently?
  - This analysis – assessing changes and then adjusting statistically for those underlying differences where changes are seen – will adjust for *time-variant* factors that changed over time.   The geographic clustering in the DID model only adjusts for time *invariant* changes over time.

1. Prepared by Rahul Rawat and Purnima Menon, IFPRI. [↑](#footnote-ref-1)
2. Note on July 14, 2016: Parts of the impact analysis plan that specifically cover the breastfeeding analyses reported in PLoS Medine manuscript are highlighted. [↑](#footnote-ref-2)
